# Semi-synthetic terpenoids with differential adjuvant properties as sustainable replacements for shark squalene in vaccine emulsions

**DOI:** 10.1038/s41541-023-00608-y

**Published:** 2023-02-16

**Authors:** Karl J. Fisher, Robert Kinsey, Raodoh Mohamath, Tony Phan, Hong Liang, Mark T. Orr, William R. Lykins, Jeffrey A. Guderian, Julie Bakken, David Argilla, Gabi Ramer-Denisoff, Elise Larson, Yizhi Qi, Sandra Sivananthan, Karina Smolyar, Darrick Carter, Christopher J. Paddon, Christopher B. Fox

**Affiliations:** 1 Amyris, Inc., Emeryville, CA USA; 2 Access to Advanced Health Institute, formerly Infectious Disease Research Institute, Seattle, WA USA; 3 Infectious Disease Research Institute, Seattle, WA USA; 4 Department of Global Health, University of Washington, Seattle, WA USA; 5 Present Address: Neoleukin, Seattle, WA USA; 6 Present Address: Bristol-Myers Squibb, Seattle, WA USA; 7 Present Address: HDT Bio Corp., Seattle, WA USA; 8 Present Address: PAI Life Sciences Inc., Seattle, WA USA

**Keywords:** Adjuvants, Vaccines

## Abstract

Synthetic biology has allowed for the industrial production of supply-limited sesquiterpenoids such as the antimalarial drug artemisinin and β-farnesene. One of the only unmodified animal products used in medicine is squalene, a triterpenoid derived from shark liver oil, which when formulated into an emulsion is used as a vaccine adjuvant to enhance immune responses in licensed vaccines. However, overfishing is depleting deep-sea shark populations, leading to potential supply problems for squalene. We chemically generated over 20 squalene analogues from fermentation-derived β-farnesene and evaluated adjuvant activity of the emulsified compounds compared to shark squalene emulsion. By employing a desirability function approach that incorporated multiple immune readouts, we identified analogues with enhanced, equivalent, or decreased adjuvant activity compared to shark squalene emulsion. Availability of a library of structurally related analogues allowed elucidation of structure-function relationships. Thus, combining industrial synthetic biology with chemistry and immunology enabled generation of sustainable terpenoid-based vaccine adjuvants comparable to current shark squalene-based adjuvants while illuminating structural properties important for adjuvant activity.

## Introduction

Squalene ((6*E*,10*E*,14*E*,18*E*)-2,6,10,15,19,23-hexamethyltetracosa-2,6,10,14,18,22-hexaene) is a triterpene and belongs to the large natural product family of terpenoids of which there are over 55,000 known members^1^. Many terpenoids are produced at low concentrations in plants, making their industrial production economically infeasible, but squalene is unusual in that it occurs at high concentrations in shark liver oil from which it has traditionally been isolated^2^. Shark-derived squalene has been used as a vaccine adjuvant in hundreds of millions of influenza vaccine doses, demonstrating an excellent safety profile and vaccine dose-sparing capability^3^. Moreover, multiple late-stage COVID-19 vaccine candidates that include shark squalene-based formulations are expected to play an important role in the global pandemic response due—in part—to stability profiles that enable easier distribution logistics (i.e., refrigerated, not frozen) than the currently authorized mRNA vaccines^4^. Shark squalene is also a key component in vaccine candidates under clinical evaluation for other indications, including tuberculosis, malaria, schistosomiasis, and leishmaniasis^5^. Indeed, next to aluminum salts, shark squalene-based emulsions are the most widely employed vaccine adjuvant formulation in licensed inactivated or protein-based vaccines^3^. However, shark populations have been subject to overfishing, and the global abundance of oceanic sharks and rays has declined by 71% since 1970, resulting in calls for prohibitions and precautionary catch limits to avert population collapse^6^. A replacement for shark squalene with equivalent or better vaccine adjuvant properties when appropriately formulated, but sourced sustainably, would be desirable to alleviate the population pressure on sharks and secure the long-term future of this class of vaccine adjuvants.

Synthetic biology applied to metabolic engineering has resulted in industrial-scale production volumes of natural products that were hitherto either subject to supply constraints or unavailable in the quantities and purities required for commercial usage. An early example of the development and use of bioengineered yeast to produce a terpenoid at commercial scale was the semi-synthetic version of the antimalarial drug artemisinin, whereby yeast (*Saccharomyces cerevisiae*) was engineered to produce the sesquiterpenoid artemisinic acid by fermentation, which was then converted chemically to artemisinin^7,8^. Subsequently, *S. cerevisiae* was engineered to produce the sesquiterpene β-farnesene ((6*E*)-7,11-dimethyl-3-methylidenedodeca-1,6,10-triene) by fermentation at high yield and productivity^9^, leading to its commercial manufacture^10^. A major commercial product derived from β-farnesene by semi-synthesis is high-purity (92–94% with >99% saturated triterpene content) squalane, which provides a sustainable, renewable source of this emollient for the cosmetic market and displaces shark-derived squalane, manufactured by hydrogenation of shark-derived squalene^11^.

Despite the importance of shark squalene emulsion adjuvants in contemporary human vaccines, their mechanisms of action are not completely understood. For instance, it has been shown that shark squalene-based emulsions increase vaccine antigen uptake, enhance recruitment and activation of various immune cells at the injection site and the draining lymph node, and cause production of danger-associated molecular patterns that result in proinflammatory signaling cascades^12^; however, it is not clear how the structural properties of squalene relate to these mechanisms.

We leveraged the ready availability of fermentation-derived, isomerically pure β-farnesene to investigate the structure-activity relationship (SAR) of squalene analogues as vaccine adjuvant components. As the β-farnesene feedstock is derived from sugarcane by yeast fermentation^10^, the terpene moieties of these molecules are renewable and sustainably produced. Additional terpenoids were obtained from alternative non-animal sources, including plants or synthetic chemistry. Our initial investigation started with two kinds of farnesene dimers: dehydroisosqualene (DHIS) and farnesene thermal dimers (Table 1). DHIS is structurally similar to squalene with one extra double bond and some differences in the location and geometry of the double bonds in the center of the molecule. Similarly, farnesene thermal dimers each feature a farnesyl analogue and a geranyl sidechain (C10) group in common with squalene with different hydrocarbon groups connecting the two chains. Preliminary evaluation of these two materials formulated in oil-in-water emulsions encouraged further exploration of the structure-activity relationships of additional compounds similar to squalene. Specifically, we sought to assess the impact of (1) variations in the overall length of terpene analogues, (2) conformationally restricted versions of squalene to learn about the preferred geometry of the pharmacophore, (3) saturation of the double bonds in the squalene structure, (4) hydrophobicity (logP) and charge on the activity of a series of analogues, and (5) inserting chain-extending core substituents to increase the overall length of the molecule.

**Table 1:** Structural properties, source, and purity of terpenoid oils.

Compound name [Number]	Source	Structure^a^	MW^b^ (# carbons)	cLogP^a^	% Purity
Squalene [**1**]	shark		411 (C_30_)	12.9	99
Dehydroisosqualene (DHIS) [**2**]	semi-synthetic (yeast)		408 (C_30_)	12.5	90
Farnesene thermal dimers [**3**–**6**]	semi-synthetic (yeast)	Mixture of 4 isomers	408 (C_30_)	*ca*. 12.5	>95
C_20_ dimer [**7**]	synthetic		272 (C_20_)	*ca*. 8.4	92
C_25_ dimer [**8**]	semi-synthetic (yeast)	Mixture of 2 main isomers	340 (C_25_)	*ca*. 10.4	85
Nonaprenol (solanesol) [**9**]	plant		631 (C_45_)	17.2	≥93
Alcohols A [**10**]	semi-synthetic (yeast)	Racemic, mixture of 2 diastereoisomers	466 (C33)	12.1	>90
Squalane [**11**]	shark or semi-synthetic (yeast)		423 (C_30_)	15.8	92
Difarnesyl ether [**12**]	semi-synthetic (yeast)		426 (C30)	13.4	94
Alcohols C [**13**]	semi-synthetic (yeast)	Racemic, mixture of 2 diastereoisomers	480 (C34)	12.6	96
Aldehydes A [**14**]	semi-synthetic (yeast)	Racemic, mixture of 2 diastereoisomers	464 (C33)	12.0	96
Compounds 1 [**15**]	semi-synthetic (yeast)	Racemic, mixture of 2 diastereoisomers	522 (C34)	NA^c^	>90
Acids A [**16**]	semi-synthetic (yeast)	Racemic, mixture of 2 diastereoisomers	480 (C33)	NA	>93
Acids C [**17**]	semi-synthetic (yeast)	Racemic, mixture of 2 diastereoisomers	566 (C37)	NA	>93
Esters A [**18**]	semi-synthetic (yeast)	Racemic, mixture of 2 diastereoisomers	536 (C37)	14.0	93
Diols B [**19**]	semi-synthetic (yeast)	Racemic, mixture of 2 diastereoisomers	494 (C34)	10.6	94
Compounds 3 [**20**]	semi-synthetic (yeast)	Racemic, mixture, average m + n = 9.5	NA	9.2	>90
Diesters B [**22**]	semi-synthetic (yeast)	Racemic, mixture of 2 diastereoisomers	553 (C36)	12.0	99
Alcohol D [**23**]	semi-synthetic (yeast)		455 (C32)	11.8	82
Ether 2 [**24**]	semi-synthetic (yeast)		519 (C36)	13.7	95
Ether 3 [**25**]	semi-synthetic (yeast)		471 (C32)	11.2	99
Ether 4 [**26**]	semi-synthetic (yeast)		497 (C34)	11.4	98
Ether 5 [**27**]	semi-synthetic (yeast)		519 (C36)	13.3	96
Ether 6 [**28**]	semi-synthetic (yeast)		739 (C51)	19.4	96
Long-chain triglyceride (negative control)	grapeseed	Representative: comprises mixture of structures	806–818^d^	22.0–24.3^d^	NA

^a^Structures drawn and cLogP values calculated using ChemDraw software (v1.0).

^b^MW = molecular weight.

^c^NA = not applicable.

^d^Estimated range based on reported composition^38^.

We formulated terpenoids with emulsifiers and other excipients under high-pressure homogenization to generate oil-in-water nanoemulsions. Following assessment of emulsion physicochemical stability, the biological activity of the emulsions was evaluated by measuring innate immune responses in human whole blood stimulated with the emulsions as well as by characterizing adaptive immune responses in mice immunized with a split, inactivated influenza vaccine mixed with the emulsions. We incorporated multiple immunological readouts into a desirability function to provide an overall rank score for each compound compared to shark squalene. This work identifies non-shark-derived triterpenoids as vaccine adjuvant raw materials with promising stability and biological activity profiles, elucidates structure-function relationships for squalene-like terpenoids, and represents a major step forward from our previous reports of potential squalene alternatives for pharmaceutical use^13–15^.

## Results

### Compound synthesis and characterization

The structures of synthesized compounds are shown in Table 1. Synthesis procedures, characterization results, and International Union of Pure and Applied Chemistry (IUPAC) names for each compound are described in the Supplementary Information (Supplementary Methods and Supplementary Table 1). In general, purified compounds were characterized by gas chromatography with flame ionization detection (GC-FID), high-performance liquid chromatography (HPLC), 1H and 13 C nuclear magnetic resonance (NMR), and mass spectrometry (MS). All synthesized compounds achieved a purity of ≥90% as determined by GC-FID or HPLC-MS, with the exception of two compounds that were obtained at 82–85% purity (Table 1). Plant-derived solanesol was obtained from a commercial vendor at a purity of ≥93% as reported by the manufacturer using HPLC.

### Emulsion formulation and physicochemical stability

Terpenoid oils were formulated with excipients that comprise the previously reported composition known as stable emulsion (SE), consisting of a mixture of emulsifiers (dimyristoyl phosphatidylcholine (DMPC) and poloxamer 188), an antioxidant agent (α-tocopherol), a tonicity agent (glycerol), and a buffer system (25 mM ammonium phosphate, pH 5.8), and processed by high-pressure homogenization to generate 4% v/v oil-in-water emulsions^16^. A non-terpenoid SE based on a long-chain triglyceride oil (grapeseed) that was previously shown to have no immunostimulatory properties was included as a negative control^15,17^. An alternative surfactant composition based on the adjuvant emulsion known as MF59 was employed for some terpenoids (consisting of polysorbate 80, sorbitan trioleate, and citrate buffer^3^).

Emulsion droplet diameter and polydispersity index among all successfully formulated oils were highly similar to the shark squalene and long-chain triglyceride emulsion controls for SE and MF59-like preparations (Table 2 and Supplementary Figs. 1–2). For instance, droplet sizes of all successfully filtered SE formulations were 71–126 nm compared to 91 nm for shark squalene SE. Likewise, droplet sizes of MF59-like compositions were 151–199 nm compared to 144 nm for shark squalene MF59-like emulsion. Some terpenoid oils were not successfully formulated in the SE excipient composition: compounds 1 and ether 6 were too viscous to be formulated, and acids C and diols B resulted in emulsions with average droplet diameters >200 nm and high polydispersity index values, which prevented terminal filtration through a 0.22-µm polyethersulfone membrane. Where possible, alternative approaches were devised to circumvent these challenges. Thus, in the case of diols B, acceptable emulsification (droplet diameter <200 nm) was achieved by replacing the SE excipient composition described above with the MF59-like composition. Similarly, diesters B—which is structurally related to diols B—was formulated in the MF59-like excipient composition. In the case of ether 6, the oil viscosity was decreased by mixing 1:1 v:v with the long-chain triglyceride oil. Emulsions generally exhibited negative zeta potentials (Table 2).

**Table 2:** Emulsion physical characteristics.

Formulation	Droplet Diameter (Z-ave, nm)	Size Polydispersity Index (PdI)	Zeta Potential (mV)^a^
Shark squalene SE	90.9 + /− 1.4	0.043 + /− 0.018	−13.3 + /− 1.6
Shark squalane SE	102.6 + /− 0.8	0.037 + /− 0.022	−5.0 + /− 0.6
Squalane SE	109.9 + /− 1.4	0.045 + /− 0.015	−3.8 + /− 1.0
DHIS SE	81.6 + /− 1.7	0.038 + /− 0.016	−15.1 + /− 3.2
Solanesol SE	99.3 + /− 3.6	0.102 + /− 0.018	−8.2 + /− 0.3
C25 dimer SE	87.9 + /− 1.2	0.046 + /− 0.025	−21.6 + /− 3.3
C20 dimer SE	109.3 + /− 2.8	0.082 + /− 0.018	−27.8 + /− 1.0
Farnesene thermal dimers SE	96.9 + /− 2.0	0.050 + /− 0.007	−19.7 + /− 1.7
Alcohols A SE	106.7 + /− 1.6	0.055 + /− 0.018	−4.9 + /− 1.5
Alcohols C SE	125.5 + /− 2.3	0.083 + /− 0.010	−13.0 + /− 0.4
Alcohols D SE	78.2 + /− 1.6	0.062 + /− 0.013	–
Diols B SE	214.9 + /− 3.9	0.232 + /− 0.019	–
Esters A SE	101.5 + /− 1.0	0.055 + /− 0.013	−8.8 + /− 0.6
Aldehydes A SE	83.5 + /− 1.7	0.061 + /− 0.018	−29.6 + /− 1.5
Acids A SE	122.1 + /− 2.9	0.070 + /− 0.021	−19.8 + /− 0.9
Acids C SE	217.2 + /− 7.4	0.185 + /− 0.004	–
Compounds 3 SE	90.9 + /− 1.8	0.134 + /− 0.007	−2.1 + /− 0.0
Difarnesyl ether SE	71.2 + /− 1.2	0.042 + /− 0.017	−7.8 + /− 1.0
Ether 2 SE	102.4 + /− 2.8	0.053 + /− 0.023	−4.5 + /− 0.4
Ether 3 SE	79.1 + /− 1.3	0.043 + /− 0.011	−6.9 + /− 2.3
Ether 4 SE	85.9 + /− 2.4	0.047 + /− 0.016	−7.4 + /− 0.4
Ether 5 SE	90.5 + /− 2.3	0.061 + /− 0.011	−1.3 + /− 0.3
Ether 6/long-chain triglyceride SE	104.5 + /− 3.0	0.069 + /− 0.012	−4.2 + /− 0.2
Squalene/long-chain triglyceride SE	87.9 + /− 2.4	0.044 + /− 0.018	−3.0 + /− 0.3
Long-chain triglyceride SE	95.2 + /− 1.7	0.068 + /− 0.019	−3.1 + /− 0.1
Shark squalene MF59-like	143.9 + /− 3.7	0.026 + /− 0.016	−29.2 + /− 0.5
Diols B MF59-like	199.4 + /− 5.0	0.043 + /− 0.027	−17.7 + /− 0.4
Diesters B MF59-like	153.4 + /− 3.7	0.053 + /− 0.019	−22.4 + /− 0.2
Long-chain triglyceride MF59-like	150.5 + /− 4.0	0.035 + /− 0.019	−25.1 + /− 0.2

^a^The age of the emulsions at the time of zeta potential measurement was not controlled. Some emulsions were not stable at 5 °C or did not have an initial particle diameter of <200 nm and thus were not measured for zeta potential.

**Fig. 1:**
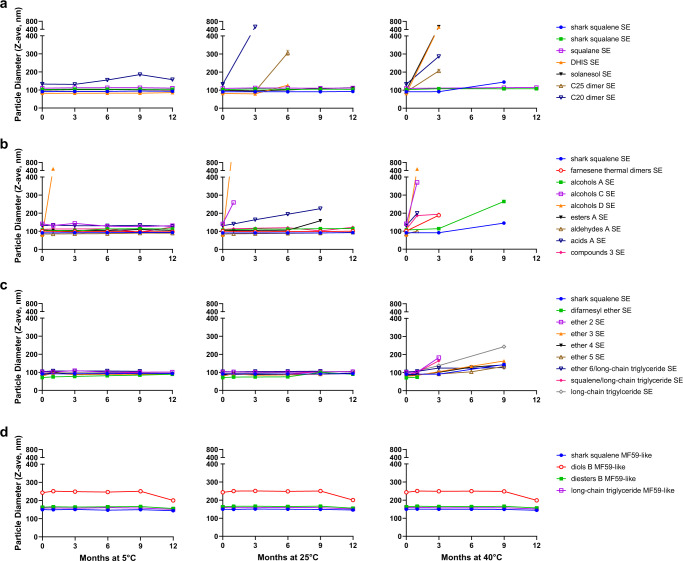
Physical stability of oil-in-water emulsions. Emulsions were stored at 5 °C, 25 °C, or 40 °C for up to 12 months and monitored by dynamic light scattering to assess emulsion droplet diameter. Data are represented as means with error bars representing standard deviation of the measurement. **a** Linear terpenoid SE series, **b** cyclohexene terpenoid SE series, **c** ether terpenoid SE series, **d** MF59-like formulations of selected cyclohexene-containing terpenoids.

For successfully formulated emulsions, a minimum stability benchmark of <20% droplet diameter growth after storage for 3 months at 5 °C was established to determine which emulsions should be tested for biological activity. The average droplet diameter and polydispersity were monitored over time on emulsions stored at 5 °C, 25 °C, and 40 °C (Fig. 1 and Supplementary Fig. 2). Many terpenoid oil emulsions demonstrated comparable physicochemical stability as shark-derived squalene emulsion when stored at 5 °C and 25 °C. At 40 °C, squalane SE formulations and all MF59-like compositions demonstrated the most robust physical stability, followed by the shark squalene SE, the ether SEs, and alcohols A SE. Terpenoid oils that resulted in physically unstable emulsions as evidenced by increasing droplet diameter, polydispersity index, and/or phase separation were removed from further stability testing. SEs made with C20 dimer, C25 dimer, DHIS, solanesol, acids A, alcohols C, or esters A were less physically stable than the shark squalene SE at elevated temperatures. At 5 °C storage, alcohols D emulsion was physically unstable within 1 month following manufacture, and compounds 3 emulsion evidenced visible particulates after ~7 months. Finally, a selection of emulsified oils was monitored for chemical stability by HPLC with charged aerosol detection (Supplementary Fig. 2). In this regard, solanesol demonstrated comparable chemical stability to shark squalene and squalane at all temperatures.

### In vitro innate immune stimulation activity

**Fig. 2:**
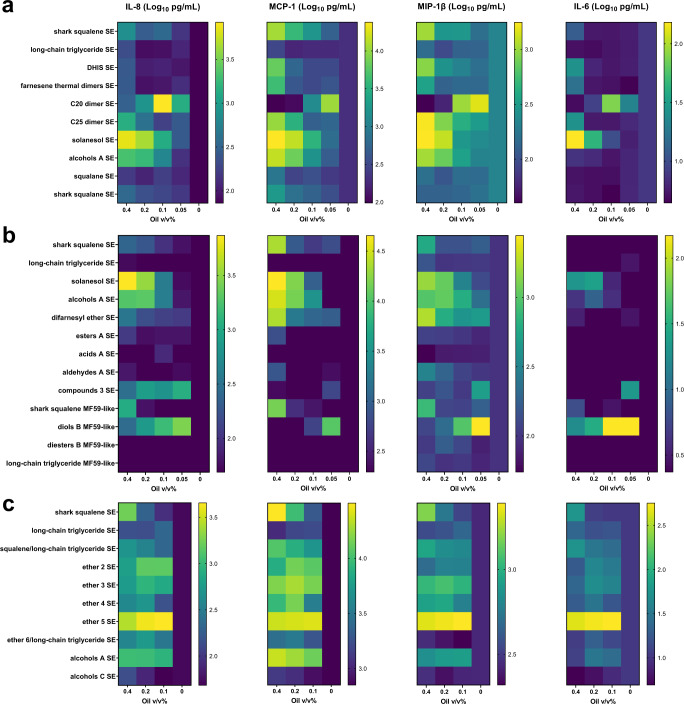
In vitro stimulation of human whole blood with terpenoid oil emulsions results in differential cytokine activity. Heat map representation of log-transformed cytokine concentrations measured in supernatants of heparinized blood stimulated by incubation with different % v/v terpenoid emulsions or saline alone (represented as 0% v/v oil). The various terpenoid oils were evaluated in comparison to shark squalene emulsion (positive control) and long-chain triglyceride emulsion (negative control). **a** Cytokine profiles elicited by a first set of terpenoid oils. Values represent the mean from 12 donors (six male and six female). **b** Cytokine profiles elicited by a second set of terpenoid oils. Values represent the mean from 6 donors (three male and three female). **c** Cytokine profiles elicited by a third set of terpenoid oils. Values represent the mean from 12 donors (six male and six female).

Emulsions demonstrating physical stability (<20% growth in diameter) for a minimum of 3 months when stored at 5 °C were selected for evaluation of innate immunomodulatory activity on whole blood from human subjects. Three experimental sets of emulsions were incubated with heparinized blood for 18–24 h at 37 °C, and supernatants were subsequently assessed for production of interleukin (IL)-8, monocyte chemoattractant protein-1 (MCP-1), macrophage inflammatory protein-1β (MIP-1β), and IL-6 cytokines. These cytokines were selected as potential indicators of innate stimulation rather than a comprehensive profile of all cytokine responses. Various terpenoid oil emulsions demonstrated comparable or enhanced dose-dependent innate immune stimulation compared to the shark squalene emulsion, whereas other terpenoid oil emulsions resulted in little or no innate immune stimulation (Fig. 2). In some cases (e.g., C20 dimer SE, compounds 3 SE, and diols B MF59-like), emulsions elicited high cytokine levels at low doses but little or no cytokine induction at high doses. To assess cytolytic behavior, selected emulsions were evaluated for their effect on viability of human peripheral blood mononuclear cells (PBMCs). Several emulsions along with shark squalene SE had a minimal impact on PBMC viability, whereas some emulsions (C20 dimer SE, compounds 3 SE, diols B MF59-like, and acids A SE) substantially decreased cell viability (Supplementary Fig. 3).

### In vivo adjuvanted influenza vaccine immunogenicity

In preparation for vaccination studies in the mouse model, selected emulsions were mixed with split, inactivated H5N1 vaccine antigen and monitored for physicochemical compatibility for up to 24 h after mixing, with emulsion droplet diameter measured by dynamic light scattering and antigen conformation assessed by single radial immunodiffusion (SRID). Adjuvant particle size and antigen conformation demonstrated only minor changes 24 h after mixing (Supplementary Fig. 4). In order to determine an optimal antigen dose for revealing the impact of an adjuvant, female C57BL/6 mice (5/group) were immunized intramuscularly with 1, 0.1, or 0.01 µg of hemagglutinin (HA) equivalent in the split, inactivated H5N1 vaccine, with or without the shark squalene emulsion. The ability of the shark squalene emulsion to significantly enhance antigen-specific serum antibody titers, functional hemagglutination inhibition (HAI) titers, and long-lived antibody-secreting plasma cells was most evident at the 0.01-µg HA dose (Supplementary Fig. 5).

**Fig. 3:**
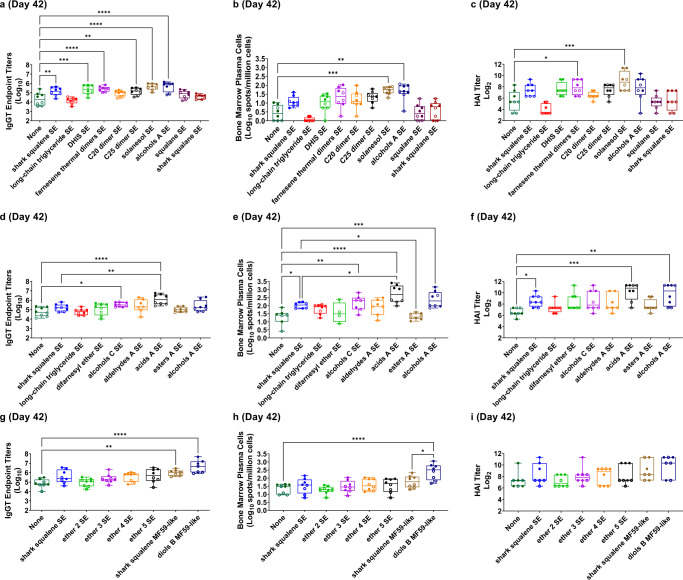
Effects of terpenoid oil emulsions on antigen-specific HAI, total IgG, and long-lived antibody-secreting plasma cells in mice immunized intramuscularly with split, inactivated H5N1 influenza antigen mixed with terpenoid emulsions. Vaccine antigen was mixed with the indicated terpenoid oil emulsion immediately prior to intramuscular immunization of C57BL/6 mice (male [open circles] and female [closed circles]) such that each animal received 10 ng of antigen in 100 µL of 2% v/v terpenoid emulsion. Negative control groups received antigen alone (None) or antigen mixed with long-chain triglyceride emulsion. Three separate experiments each with different terpenoids as indicated are represented (**a**–**c**, **d**–**f**, and **g**–**i**). Data are represented as box-whisker plots with bars representing median values, boxes representing 1st–3rd quartiles, and whiskers representing the maximum and minimum values. Statistical evaluation of each terpenoid emulsion group compared to antigen alone (negative control) and shark squalene emulsion (positive control) was performed by one-way ANOVA with Sidak’s correction for multiple comparisons or Kruskal–Wallis test with Dunn’s correction for multiple comparisons as indicated; *p-value < 0.05, **p-value < 0.01, ***p-value < 0.001, ****p-value < 0.0001. **a**, **d**, **g** Antigen-specific total IgG (IgGT) serum titers measured by ELISA 42 days after prime immunization (*n* = 7–8 mice, one-way ANOVA). **b**, **e**, **h** Long-lived antibody-secreting cells in the bone marrow measured by ELISpot assay 42 days after prime immunization (*n* = 8 mice, Kruskal–Wallis for **b**; *n* = 4–8 mice, one-way ANOVA for **e**; *n* = 8 mice, one-way ANOVA for **h**). **c**, **f**, **i** HAI titers measured 42 days after prime immunization (*n* = 8 mice, one-way ANOVA for **c**; *n* = 7–8 mice, Kruskal–Wallis for **f**, **i**). See also Supplementary Figs. 7–9.

Based on their stability performance and in vitro bioactivity, 17 emulsions with appropriate controls were selected for in vivo evaluation with split, inactivated H5N1 antigen. Using 0.01 µg as the antigen dose based on the previous study, C57BL/6 mice (4 male and 4 female mice per group) were immunized intramuscularly at Day 0 and Day 21 with influenza vaccine antigen alone or the vaccine antigen mixed with the indicated oil-in-water emulsions at 2% v/v oil in three separate experiments, each involving a different experimental set of oils (Supplementary Fig. 6 and Supplementary Table 2). Three weeks following the first immunization, blood was collected to assess antigen-specific serum antibody titers and functional serum HAI titers. Three weeks following the second immunization, blood, bone marrow, and spleen samples were acquired to assess antigen-specific serum antibody titers and functional serum HAI titers, long-lived antibody-secreting plasma cells, and cytokine production from antigen-stimulated splenocytes, respectively. After two immunizations, various terpenoid emulsions elicited significantly enhanced antigen-specific serum immunoglobulin (Ig)G, HAI, and/or long-lived antibody-secreting plasma cells compared to vaccine antigen alone, and comparable (or enhanced in the case of acids A SE) to the effects of shark squalene emulsion (Fig. 3). Although significant augmentation of antigen-specific serum IgG isotypes (IgG1 and/or IgG2c) was evident for many emulsions, there was no statistically significant shift in the IgG2c/IgG1 titer ratio (Supplementary Fig. 7), and cytokine levels from stimulated splenocytes were generally low, confirming that there was no definitive shift in T_H_ bias of the immune response (Supplementary Fig. 8). Immune responses measured 3 weeks after a single immunization exhibited trends consistent with the results obtained following the second immunization (Supplementary Fig. 9).

To generate a meaningful overall ranking of the emulsions tested, involving multiple in vivo immunological readouts and three separate in vivo experiments, we employed a desirability function approach adapted from previous reports^18,19^. Briefly, data from each readout were log-transformed, the average values from each experimental group were ratioed to the average shark squalene emulsion value from the same experiment, and the resulting values were normalized on a unitless scale of 0–1. A weighted composite desirability score was then calculated using the equation $D = \root {p} \of {{d_1^{w_1} \times d_2^{w_2} \times \ldots \times d_n^{w_n}}}$ where *d*_*i*_ = partial desirability attributed to the *i*th immunological response (*i* = 1; 2;…; *n*), *w*_*i*_ = weighting attributed to the *i*th response, and *p* = $\mathop {\sum}\nolimits_1^n {w_i}$^20^. The weighting system was designed to rank the various immunological readouts, with 5 representing the greatest importance and 1 representing the least importance (Table 3). For example, HAI titers are considered a correlate of protection for influenza vaccines^21^ and were thus given the highest weight. Nevertheless, the weighting approach is subjective and may be tailored based on the desired target immune profile. Thus, an editable desirability function spreadsheet is located in the Supplementary Data, allowing user modification of the weighting factors to determine the impact on the overall desirability ranking of the terpenoid formulations tested here.

**Table 3:** Desirability function weighting scheme.

Readout	Weight	Function	Justification
HAI (Day 42)	5	Maximize	Functional readout correlated with protection from influenza
Long-lived antibody-secreting cells (Day 42)	4	Maximize	Long-lived plasma cells are necessary for durable antibody-mediated immunity
IgGT (Day 42)	4	Maximize	Serum IgG antibody titers are indicative of systemic immunogenicity
IgG2c/IgG1 (Day 42)	3	Maximize	IgG2c/IgG1 ratio correlates with Th1 immunity
HAI (Day 21)	2	Maximize	Functional readout correlated with protection from influenza
IgGT (Day 21)	1	Maximize	Serum IgG antibody titers are indicative of systemic immunogenicity

**Fig. 4:**
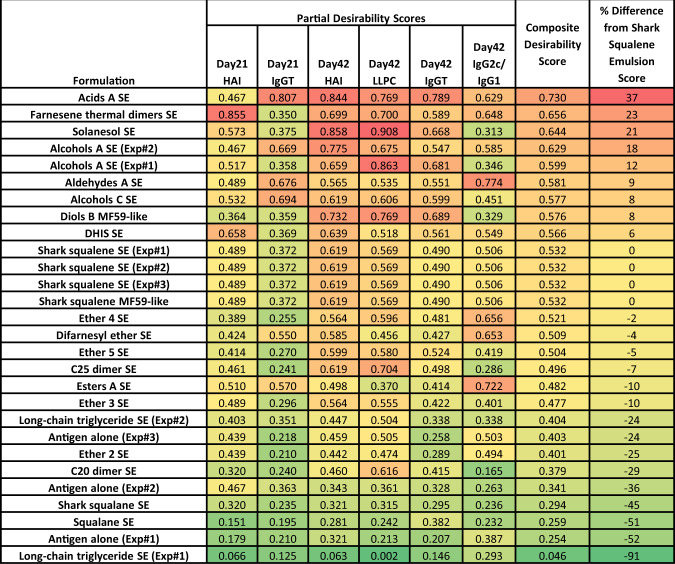
Desirability index scores calculated from mouse immunogenicity studies. Values are colored from green to red to represent low to high scores. Experiment (Exp) number is provided for terpenoid emulsions evaluated in more than one mouse experiment. Abbreviations: HAI hemagglutination inhibition titer, IgGT total antigen-specific serum immunoglobulin G, LLPC long-lived antibody-secreting plasma cells.

The partial and composite desirability scores representing all formulations tested in vivo are shown in Fig. 4, employing the weighting scheme from Table 3. The desirability scores for the shark squalene emulsion groups from each experiment are by definition the same. The composite desirability ranking can be used to broadly categorize emulsions that elicited enhanced, equivalent, or reduced immune responses compared to shark squalene emulsion. Thus, emulsions made with acids A, farnesene thermal dimers, solanesol, or alcohols A performed substantially or somewhat better than shark squalene emulsion (>10% improvement in composite desirability score); emulsions made with aldehydes A, alcohols C, diols B, DHIS, ether 4, difarnesyl ether, ether 5, C25 dimer, esters A, and ether 3 performed similar to shark squalene emulsion (≤10% change in composite desirability score); and emulsions made with long-chain triglyceride, ether 2, C20 dimer, shark squalane, squalane, or without any emulsion (antigen alone) performed poorly compared to shark squalene emulsion (>10% decrease in composite desirability score). Moreover, partial desirability scores representing specific immunological readouts indicate that rapid antibody immune responses were elicited by acids A and farnesene thermal dimers emulsions, whereas solanesol emulsion may favor response durability and aldehydes A the highest IgG2c/IgG1 ratio.

### Correlations

**Fig. 5:**
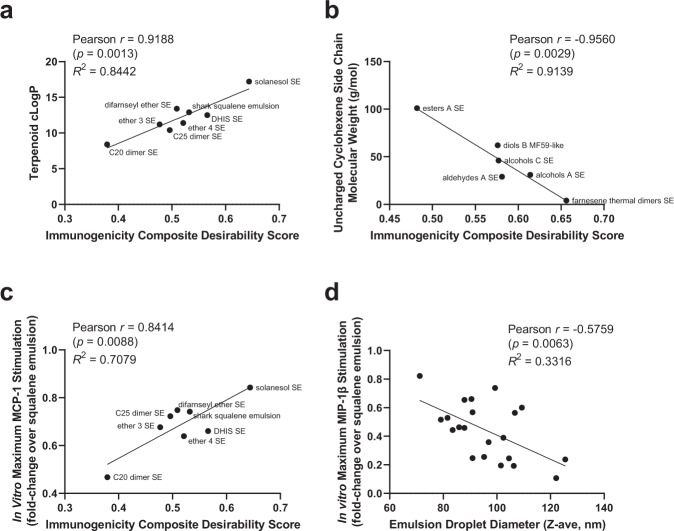
Structure-function correlations. **a** Immunogenicity composite desirability score of linear unsaturated terpenoids correlates with terpenoid cLogP. **b** Side chain molecular weight of cyclohexene-containing terpenoids inversely correlates with immunogenicity composite desirability score. **c** In vitro human whole blood MCP-1 stimulation activity of linear unsaturated terpenoid emulsions correlates with in vivo composite desirability score. **d** In vitro human whole blood MIP-1β stimulation activity of SE formulations correlates with emulsion droplet diameter. For all correlation plots, a single mean value was computed for structures that were included in more than one experiment (antigen alone, shark squalene SE/MF59-like, shark squalane/squalane SE, long-chain triglyceride SE, alcohols A SE, and solanesol SE) such that each terpenoid structure is represented only once.

Several structural features correlating with changes in adjuvant activity in vivo were identified. First, for unsaturated linear terpenoid emulsions, adjuvant activity was strongly associated with chain length (represented by cLogP, molecular weight, or number of carbons, see Fig. 5a and Supplementary Fig. 10a, b). Thus, C20 and C25 analogues of DHIS showed lower composite desirability scores than squalene or DHIS, whereas the C45 solanesol substantially enhanced responses, with a ranking of C20 dimer < C25 dimer < C30 squalene < C30 DHIS < C45 solanesol.

Second, we considered that conformationally restricted analogues such as acids A, alcohols A, aldehydes A, alcohols C, and esters A may impact bioactivity since they are more similar to the extended conformation of squalene at C11-C12 rather than a folded conformation at C11-C12 (squalene can exist in both conformations, see Supplementary Fig. 11). Clearly, this set of compounds has members with good to excellent activity (Fig. 4), indicating that the C11-C12 folded conformation is not required for activity. Thus, these results support the C11-C12 extended form as the active conformation of squalene.

Third, dramatically reduced adjuvant activity was associated with chain saturation as evidenced by comparing the composite desirability scores of unsaturated shark squalene emulsion with emulsions made from saturated squalane (shark-derived or semi-synthetic, see Table 1 and Fig. 4). Nevertheless, alterations in the double bond configuration did not appear to have detrimental impacts to bioactivity, as evidenced by the highly similar composite desirability scores of shark squalene and DHIS emulsions.

Fourth, the influence of charge was dramatic, with acids A SE achieving the highest composite desirability score (Fig. 4). Unfortunately, the other charged compounds did not generate stable emulsions and were thus not evaluated for bioactivity.

Fifth, a range of different connecting chain lengths in a series of a mono ether and diethers of farnesyl chloride made by alkylation of alcohols and phenols was tolerated, with some emulsified compounds demonstrating similar activity to shark squalene emulsion (Supplementary Table 3). Interestingly, the geometry of main chain linkage to the central ring structure had a substantial impact on composite desirability score, with ether 5 (1,2-linkage) outperforming ether 2 (1,3-linkage). Moreover, adjuvant activity was at least maintained and possibly enhanced with the introduction of a central ring structure: for instance, the cyclohexene-containing farnesene thermal dimers exhibited a higher composite desirability score than shark squalene or DHIS, and the benzene-containing ether 5 performed similarly to ether 4 or shark squalene (Fig. 4). However, for uncharged cyclohexene-containing terpenoids, the molecular weight of the side chain conjugated to the cyclohexene was inversely correlated with the composite desirability score (Fig. 5b), indicating a steric effect could be causing activity reduction.

Although several terpenoid emulsions stimulated innate responses from human cells in vitro and adaptive responses from immunized mice in vivo comparable to shark squalene emulsion, there was not a strong correlation between these readouts covering all emulsions tested. For instance, the top performer in the mouse immunogenicity studies was the acids A emulsion, but little or no cytokine stimulation was evident when this emulsion was incubated with human whole blood. On the other hand, the C20 dimer emulsion was extremely potent in vitro at low doses but was a poor performer in vivo. Nevertheless, when the analysis is limited to linear unsaturated terpenoids, a correlation between the in vivo composite desirability score and the in vitro MCP-1 cytokine stimulation was identified (Fig. 5c). Moreover, for all SE formulations, we identified an inverse correlation between in vitro cytokine production and emulsion particle size (Fig. 5d and Supplementary Fig. 10c). Thus, the ability to broadly predict adaptive in vivo immune response from the innate in vitro cytokine stimulation profile is limited, in part because in vitro results may be confounded by the effect of emulsion particle size.

## Discussion

Evaluation of adjuvant activity of squalene analogues other than squalane has only recently been reported^22^ despite the importance of squalene as an adjuvant component in commercial vaccines. For instance, the shark squalene emulsion MF59 is approved for use in a commercial influenza vaccine and boosts responses in elderly and pediatric populations^23^. The lack of tested analogues may be due in part to the structure of squalene, which is a highly symmetrical molecule with six nearly equivalent trisubstituted double bonds, meaning that attempting to achieve a highly selective industrially acceptable functionalization is not likely to succeed. However, the procedures described in the present report using yeast-produced β-farnesene as a starting material enabled the synthesis of a set of squalene-like triterpenoid analogues to explore structure-activity relationships. Maintenance of biological activity—despite terpenoid structural changes—is consistent with a previous report evaluating two novel triterpenoids^22^, and the reduced biological activity of squalane compared to squalene is consistent with a previous report that correlated biological activity with ability to induce reactive oxygen species^24^ but contradicts an earlier report describing squalene and squalane as equally effective vaccine adjuvant emulsion components^25^. In any case, the present study describes systematic structural changes other than chain saturation that resulted in substantial loss or enhancement of biological activity, thus informing a new structure-activity based understanding of squalene-like molecules. Additional work will be needed to refine the structure-activity map of squalene-like molecules. In particular, since the negatively charged acids A compound was the top performing molecule in terms of the mouse immunogenicity composite desirability score, additional study is warranted regarding the effect of charge on terpenoid adjuvant activity. A possible explanation for this striking bioactivity enhancement is that the salt of the acid may cause reordering of emulsion droplet morphology such that the anion of acids A is localized at the oil-water interface with increased bioavailability. Alternatively, it could be considered that acids A is substantially less lipophilic than the other molecules tested. All of the other molecules tested in vivo were highly lipophilic. The only other molecules with substantial hydrophilic character (acids C, compounds 1, and compounds 3) were not evaluated in vivo due to emulsion instability, although compounds 3 emulsion showed interesting in vitro bioactivity.

While acids A SE was the most potent formulation in the in vivo studies, it was not as physically stable as other leading candidates. Indeed, it is important to note that the phospholipid/surfactant emulsification systems employed here were previously optimized for shark squalene^14,26–29^. Thus, it is highly possible that development of optimized emulsifier compositions could generate emulsions with more suitable stability profiles for the leading candidates identified here, and additional work is merited in this regard. Nevertheless, most candidates were stable when stored at 5 °C, and long-term stability at elevated temperatures was demonstrated for several candidates (Fig. 1). Another potential benefit of formulation optimization would be to reduce cell viability loss evident with some candidates in the in vitro assays. While some cell necrosis and/or death may represent important mechanisms of action for squalene emulsions and other adjuvants^30–32^, formulation development may optimize both adjuvant activity and biocompatibility. The best example of this type of scenario is the saponin adjuvant QS-21, which is highly hemolytic unless formulated in cholesterol-containing platforms such as liposomes^33^. We considered the possibility that the bioactivity of aldehydes A might be enhanced if the aldehyde could provide a costimulatory signal by forming an imine bond with a T cell surface receptor amino group similar to a proposed mechanism for the saponin QS-21^34,35^, but no benefit was apparent in this regard (Fig. 4). Indeed, cellular responses overall were modest in our studies, and it is possible that inclusion of additional adjuvant components (such as TLR ligands) might be required for more potent T cell-based immunity.

Despite generating and evaluating the immunogenicity of multiple compounds, we recognize that additional testing would be necessary in future to advance such compounds as alternatives to shark squalene in vaccine products. For instance, protective efficacy testing in larger animal models of selected lead compounds would be necessary to establish that they are suitable replacements of shark squalene. Moreover, elucidation of specific mechanisms of action would be desirable in future for comparison to the various reported mechanisms of shark squalene emulsions. However, such efforts were outside the scope of the present report. Another limitation of the current work is that a semi-synthetic process for generating squalene itself was not reported as work is ongoing in this regard.

Semi-synthetic approaches to the manufacture of pharmaceuticals have been practiced for decades. Possibly the best-known examples are the semi-synthetic β-lactam antibiotics whose synthesis commences with a natural product (penicillin G) from *Penicillium chrysogenum*^36,37^. The approach we have taken is that the fermentation-derived precursor, β-farnesene, is the product of metabolic engineering and development of its industrial fermentative production from engineered yeast. The ready availability of the β-farnesene precursor enabled us to synthesize a range of squalene analogues that would have been inaccessible had the tools of synthetic biology not been applied to engineering and industrializing yeast for its production. The syntheses employed are industrially scalable, and—given the ready availability of the precursor (β-farnesene)—these compounds could be produced at commercial scale. Although synthesis of solanesol was not attempted here, we note that this molecule showed promising emulsion stability and adjuvant activity, is commercially available from solanaceous plants, and is an intermediate for the synthesis of coenzyme Q_10_ and vitamin K_2_. Shark populations are under severe ecological pressure, and an alternative source of a terpenoid adjuvant to replace shark-derived squalene would be environmentally beneficial.

In summary, we designed and synthesized >20 distinct terpenoids using a sustainable semi-synthetic approach and compared vaccine adjuvant activity of the emulsified terpenoids to shark squalene emulsion. Several of the compounds (e.g., alcohols A, farnesene thermal dimers, diols B, ether 4, and ether 5) possess similar or improved adjuvant activity properties compared to shark squalene as well as acceptable physicochemical stability, which could make them promising alternatives for shark squalene in vaccine adjuvant formulations for both human and veterinary usage. Furthermore, changes in squalene analogue structure were correlated with biological activity. Thus, these results demonstrate a path for obtaining squalene-like molecules from sustainable non-animal sources and for systematically mapping their structure-activity relationship in vaccine adjuvant emulsions.

## Methods

### Experimental design

The objectives of the study were to design and generate multiple terpenoid compounds using sustainable approaches and evaluate them for emulsion stability and vaccine adjuvant bioactivity compared to shark-derived squalene emulsion. Pre-specified criteria included a target of ≥90% compound purity as determined by GC-FID or HPLC-MS and a physical emulsion stability requirement of <20% size growth after 3 months storage at 5 °C.

### Compound synthesis and characterization

Detailed synthesis, characterization information, and IUPAC names for each compound are located in the Supplementary Information.

### Emulsion formulation

Plant-derived solanesol was obtained from TCI America. Shark-derived squalene, squalane, and sorbitan trioleate were obtained from Sigma-Aldrich. Napa Valley Naturals (Stonewall Kitchen) grapeseed oil was purchased from a local grocery store. DMPC was obtained from Lipoid. Poloxamer 188, α-tocopherol, and glycerol were purchased from Spectrum Chemical. Polysorbate 80 was obtained from NOF. Buffer components were obtained from J.T.Baker and Fluka. Split, inactivated H5N1 (A/Vietnam/1194/2004) was obtained from the National Institute for Biological Standards and Control (NIBSC). For the SE compositions, terpenoid oils were formulated with a mixture of emulsifiers (DMPC and poloxamer 188), an antioxidant agent (α-tocopherol), a tonicity agent (glycerol), and a buffer system (25 mM ammonium phosphate, pH 5.8) and processed by high-shear mixing and high-pressure homogenization to generate 4% v/v oil-in-water nanoemulsions^16^. For the MF59-like compositions, terpenoid oils were formulated with a mixture of non-ionic surfactants (polysorbate 80 and sorbitan trioleate) and a buffer system (10 mM citrate, pH 6.0), and processed by high-shear mixing and high-pressure homogenization to generate 4% v/v oil-in-water nanoemulsions^27^.

### In vitro cytokine stimulation assay

Fresh heparinized whole blood from 6–12 human volunteers (equal numbers of male and female) was obtained from Bloodworks Northwest. Biological sample collections were approved by WCG IRB. All participants reviewed and signed informed consent forms. Emulsions at the indicated % v/v concentrations (diluted with saline) were incubated with the blood for 18–24 h at 37 °C and 5% CO_2_^13,15^. After incubation, supernatants were aspirated and assessed by enzyme-linked immunosorbent assay (ELISA) kits for production of IL-6, IL-8, MCP-1 (Life Technologies #88-7066-77, 88-8086-77, 88-7399-77, respectively), and MIP-1β (R&D Systems #DY271) cytokines according to manufacturer’s instructions. Cytokine levels that were below the lowest concentration of the standard curve were assigned half the value elicited by the lowest standard curve concentration.

### Mice, immunizations, and sample collection

C57BL/6 (B6) mice were purchased from The Jackson Laboratory. Experimental groups consisted of equal numbers of 6–8-week-old male and female mice. Mice were immunized by intramuscular injection of 100 µL total volume (50 µL in each hind leg) of vaccine containing split, inactivated H5N1 A/Vietnam/1194/2004 (NIBSC) at the indicated dose and 2% v/v oil-in-water emulsion at Study Day 0 and Study Day 21. All animal experiments were performed in accordance with national and institutional guidelines for animal care of laboratory animals and approved by the Infectious Disease Research Institute Institutional Animal Care and Use Committee.

### Blood and tissue collection and processing

Peripheral blood was collected at Study Day 21 by the retro-orbital route (prior to the boost immunization) under light sedation using isoflurane and at Study Day 42 via cardiac puncture after euthanasia. Blood was collected in Sarstedt Microvette capillary blood collection tubes and centrifuged to separate the serum. Serum was stored at −20 °C until analysis. On Study Day 42, mice were euthanized by controlled administration of carbon dioxide inhalation, followed by cervical dislocation, and spleens and bone marrow were removed aseptically. Lymphocytes were isolated from the spleen using manual disruption. Red blood cells contained in spleens were lysed using Red Blood Cell Lysis Buffer (eBioscience). Lymphocytes were used for cytokine secretion ELISA (interferon (IFN)-γ and IL-5) and cytokine ELISpot (IFN-γ and IL-5) assays as described below. Bone marrow was exposed by snipping ends of harvested femurs followed by rinse and centrifugation cycles in complete Roswell Park Memorial Institute (RPMI) medium (consisting of RPMI medium supplemented with 10% v/v fetal bovine serum (FBS) and penicillin-streptomycin). The red blood cells were removed with Red Blood Cell Lysis Buffer (eBioscience). Bone marrow-derived cells were used for B-cell ELISpot assay as described below.

### Serum and cytokine ELISA

Antigen-specific total IgG (IgGT), IgG1, and IgG2c were measured in the serum samples from the immunized animals using antibodies purchased from Southern Biotech (#1031-05, 1070-05, and 1079-05, respectively) that were diluted 1:5000, 1:3000, and 1:2000, respectively. Briefly, 384-well plates were coated with 1 µg/mL recombinant H5 A/Vietnam/1194/2004 antigen (Sino Biological) overnight. The next day, plates were washed and blocked with phosphate-buffered saline (PBS) with 0.1% polysorbate 20 and 1% bovine serum albumin (BSA). After washing, plates were incubated with the serum followed by incubation with horseradish peroxidase (HRP)-conjugated antibodies and 3,3',5,5'-tetramethylbenzidine (TMB) substrate. The reaction was stopped using 1 N H_2_SO_4_ and read within 30 min using a Victor *X*4 (PerkinElmer) plate reader. Endpoint titers were interpolated using a sigmoidal curve fit and an arbitrary cutoff value of 0.5 or 0.75 depending on background signal. Endpoint titers below the standard curve range were assigned the value corresponding to 0.5*x* of the lowest standard curve dilution. Splenocytes from immunized animals were stimulated with 10 µg/mL recombinant H5 A/Vietnam/1194/2004 antigen (Sino Biological) for 72 h. IFN-γ and IL-5 were quantified in the supernatant by ELISA, according to the manufacturer’s instructions (Invitrogen #88-7314-88 and #88-7054-88, respectively).

### HAI assay

Serum samples were treated with receptor destroying enzyme (RDE) at a ratio of 1:3 v:v (sample:RDE) in a 96-well V-bottom plate. Following overnight incubation at 37 °C, samples were centrifuged at 400 x g for 1 min, and 6 parts of PBS were added for a final serum dilution of 1:10. Horse red blood cell (HRBC) suspension (10% HRBC) was prepared by washing with PBS three times, resuspending to a final concentration of 1% HRBC, and storing at 4 °C for same-day use. Treated serum samples or control sera were added to 96-well V-bottom plates in duplicate, after which 25 µL of split, inactivated H5N1 A/Vietnam/1194/2004 (NIBSC) (67 µg/mL stock diluted 1:200 in PBS) were added to each well. Plates were then incubated at ambient temperature for 15 min, after which 50 µL of 1% HRBC suspension were added to each well. Plates were then further incubated at ambient temperature undisturbed for 30–60 min. The HAI titer is the reciprocal of the greatest dilution that completely inhibits the agglutination of the HRBCs as indicated by the absence of a tear-shaped streaming of erythrocytes upon tilting of the plate.

### Bone marrow plasma cell ELISpot

ELISpot plates (Millipore) were coated with 2 µg/mL recombinant H5 A/Vietnam/1194/2004 antigen (Sino Biological) and incubated overnight at 4 °C. Plates were washed with PBS with 0.1% v/v polysorbate 20 (PBST), blocked with complete RPMI medium for 2 h at ambient temperature, and then washed again. The first and third experimental set of oils demonstrated high viability cell preparations (>66%), and single-cell suspensions were seeded at 1.0 × 10^6^ cells per well with 3-fold serial dilutions. The second set of experimental oils demonstrated low viability cell preparations (mean value: 21%), so data from single-cell suspensions were only included if the seeded cell count before dilution was above 1.0 × 10^4^ cells per well, with a multiplication factor employed to normalize to a 1.0 × 10^6^ cell count. The plates were incubated at 37 °C with 5% CO_2_ for 3 h, washed with PBST, then incubated overnight at 4 °C with HRP-conjugated anti-mouse IgG (H + L) (Southern Biotech #1031-05) diluted 1:1000. After incubation, the plates were washed with PBS and developed with 3-amino-9-ethylcarbazole (AEC) substrate kits (Vector Laboratories) according to the manufacturer’s protocol. The reaction was stopped by washing the plates with deionized water, plates were dried in the dark, and spots were counted using an automated ELISpot reader (CTL Analyzer, Cellular Technology Limited). Data were analyzed using ImmunoSpot version 7 professional software (Cellular Technology Limited).

### Splenocyte cytokine ELISpot

ELISpot plates were coated with IFN-γ (BD Biosciences #551881 or eBioscience #88-7384) and IL-5 (BD Biosciences #551880 or eBioscience #88-7825) capture antibodies, diluted 1:200, overnight at 4 °C. Plates were washed with PBST, blocked with complete RPMI medium for 2 h at ambient temperature, and then washed again. Splenocytes were plated at 2.0 × 10^5^ cells per well and were stimulated with 10 µg/mL recombinant H5 A/Vietnam/1194/2004 antigen (Sino Biological) at 37 °C with 5% CO_2_ for 48 h. The plates were washed with PBST, detection antibodies (BD Biosciences #551881 and #551880 or eBioscience #88-7384 and #88-7825), diluted 1:250, were added, and the plates were then incubated overnight at 4 °C. After incubation, plates were washed with PBST, and Avidin D (Av)-HRP (Invitrogen #50-112-3249), diluted 1:250, was added for a 45-min incubation at ambient temperature followed by a PBS wash. The plates were developed with AEC substrate kits according to the manufacturer’s protocol. The reaction was stopped by washing the plates with deionized water, plates were dried in the dark, and spots were counted using an automated ELISpot reader (CTL Analyzer). Data were analyzed using ImmunoSpot version 7 professional software (Cellular Technology Ltd).

### Statistical analysis

Adaptive immunity responses measured in vaccinated animals were log-transformed as indicated and then assessed for distribution normality by the D’Agostino-Pearson test (for group sizes of *n* = 8) or the Shapiro-Wilk test (for group sizes *n* < 8). Normally distributed data were compared by one-way ANOVA with Sidak’s correction for multiple comparisons, whereas non-normally distributed data were evaluated by the non-parametric Kruskal–Wallis test with Dunn’s correction for multiple comparisons as indicated in figure legends. Pearson’s correlation coefficient was computed on normally distributed data (tested by D’Agostino-Pearson or Shapiro-Wilk tests as described above). Statistical analysis was performed using GraphPad Prism version 9.3.1.

### Reporting summary

Further information on research design is available in the Nature Research Reporting Summary linked to this article.

## Supplementary information

Supplementary Data

Supplementary Information

REPORTING SUMMARY

## Data Availability

The datasets generated during and/or analysed during the current study are available from the corresponding author on reasonable request. Materials were transferred between institutions under a Material Transfer Agreement (MTA), and MTAs would be required for any sample requests.
