# Supplementary material for: Semi-synthetic terpenoids with differential adjuvant properties as sustainable replacements for shark squalene in vaccine emulsions
Source: NPJ Vaccines. 2023 Feb 16;8:14. doi: 10.1038/s41541-023-00608-y (PMC9935550; doi:10.1038/s41541-023-00608-y)
Supplement: Supplementary file 2 — Supplementary Information [file 41541_2023_608_MOESM2_ESM.pdf]

SUPPLEMENTARY METHODS

Emulsion stability

Emulsion droplet size and size polydispersity index were measured by dynamic light scattering using a Zetasizer S, Zetasizer ZS, or Zetasizer APS (Malvern Panalytical) on samples diluted 1:100 in water with the same sample measured repeatedly at least 3 times. Zeta potential on diluted emulsions was measured by microelectrophoresis using the Zetasizer ZS. Terpenoid oil chemical stability was assessed by reverse-phase high-performance liquid chromatography (HPLC) with charged aerosol detection (Thermo Fisher Scientific). Standards for each oil were prepared in a solvent consisting of 20 mM ammonium acetate in 66:33:1 v:v:v chloroform:methanol:acetic acid. Distinct triplicate samples were prepared for HPLC analysis by adding 20 µL of sample to 980 µL of the same solvent used for the standards (1:50 dilution). Samples were analyzed using a Shimadzu Prominence HPLC system with a charged aerosol detector (CAD). The method utilizes a Waters XBridge C18, 5 µm, 4.6 x 250 mm column at 30°C with a two-solvent system gradient consisting of mobile phase A (20 mM ammonium acetate and 1% acetic acid in 75:15:10 v:v:v methanol:chloroform:water) and mobile phase B (20 mM ammonium acetate and 1% acetic acid in 1:1 methanol:chloroform) at a flow rate of 1 mL/min. Sample injection volume was 15 µL. Injection volumes of standards were varied, and areas under the curve (AUCs) of the standard peaks were plotted and fitted by a second-order polynomial curve fit in GraphPad Prism software to create a 5-point standard curve. Terpenoid oil content of each sample was then calculated by interpolating the AUC of the peak of interest using the standard curve.

Antigen-adjuvant compatibility

Selected emulsions were mixed 1:1 with split, inactivated H5N1 antigen and assessed by dynamic light scattering for emulsion droplet size and single radial immunodiffusion (SRID) for hemagglutinin content at 0, 4, and 24 h after mixing, with mixtures stored at 2-8°C. The antigen was diluted with saline prior to mixing with emulsion such that the final hemagglutinin content in the vaccine mixture was 75 µg/mL. The materials necessary for SRID were acquired as follows: anti-H5 serum (National Institute for Biological Standards and Control), glacial acetic acid (Thermo Fisher Scientific), agarose (Lonza), Brilliant Blue R staining solution (Sigma-Aldrich), 1x phosphate-buffered saline (PBS) (KD Medical), Zwittergent 3-14 (EMD Biosciences), saline (made in-house or purchased from KD Medical), GelBond Film agarose support medium (Lonza), qualitative 1-mm and 3-mm filter paper (Whatman), methanol (Thermo Fisher Scientific), and an acrylic well-punching template and gel puncher (both made in-house). The following solutions were prepared: Destaining Solution (10:50:40 v:v:v acetic acid:methanol:water), Staining Solution (0.1% w/v Brilliant Blue R in Destaining Solution), Zwittergent-Water Solutions (20%, 10%), Zwittergent-PBS Solutions prepared by mixing the 20% and 10% Zwittergent-Water Solutions 1:9 v:v with 1x PBS, and Saline Solution (0.9% w/v sodium chloride in water). Agarose gels were prepared by boiling 1% agarose in 1x PBS and adding anti-H5 antibodies after cooling (525 µL of anti-H5 serum per gel). The agarose/anti-H5 solution was poured into a gel bond and allowed to solidify. The gel puncher and template were employed to generate 4-mm holes throughout the gel. The H5N1 antigen was mixed 45:5 v:v with 10% Zwittergent Solution, vortexed briefly at low speed, and allowed to incubate at ambient temperature for at least 30 min. The antigen-Zwittergent mixture was then diluted further with 1% Zwittergent Solution at 10:5, 10:10, and 5:15 v:v ratios. The same procedure was followed with adjuvanted H5N1 mixtures except that 20% and 2% Zwittergent Solutions were employed instead of 10% and 1% Zwittergent Solutions. Each well in the gel was loaded with 20 µL of sample-Zwittergent mixture and incubated for 18-24 h at ambient temperature. Gels were then submerged in 0.9% saline solution and placed on a rocker for ~20 min, rinsed with water on a rocker for ~10 min, then placed face-up on a flat surface and covered with four layers of fine filter paper (1 mm) and six layers of coarse filter paper (3 mm) soaked in water and a weight for 30 min, after which the same rinsing procedure was repeated. The gels were then dried for 2-4 h in a 37-45°C oven, after which they were placed into a tray with Staining Solution for 7-10 min. Finally, gels were placed in a tray with Destaining Solution until the precipitation rings were visible. The diameter of the precipitation rings was estimated by averaging two diameter measurements from different locations on each ring using ImageJ software (National Institutes of Health).

Peripheral blood mononuclear cell (PBMC) viability assay

PBMCs isolated from 3-4 donors were resuspended at 0.5-1 x 10^6 cells/mL in Roswell Park Memorial Institute (RPMI) medium with 10% fetal calf serum (FCS). Selected emulsions (0.05–0.4% oil diluted in RPMI medium and 10% FCS) were mixed with 1.0 x 10^5 PBMCs in 96-well U-bottom plates and incubated at 37°C for 18 h. The mixtures were then diluted 1:20 v:v with Guava ViaCount Reagent (Luminex) and incubated in the dark for 5 min at ambient temperature. Viability was measured using the Guava easyCyte HT (Luminex).

Compound synthesis and characterization

Dehydroisosqualene (DHIS), squalane, and farnesene thermal dimers were prepared as described in US Patent No. 7,691,792 B1.

DHIS [2]

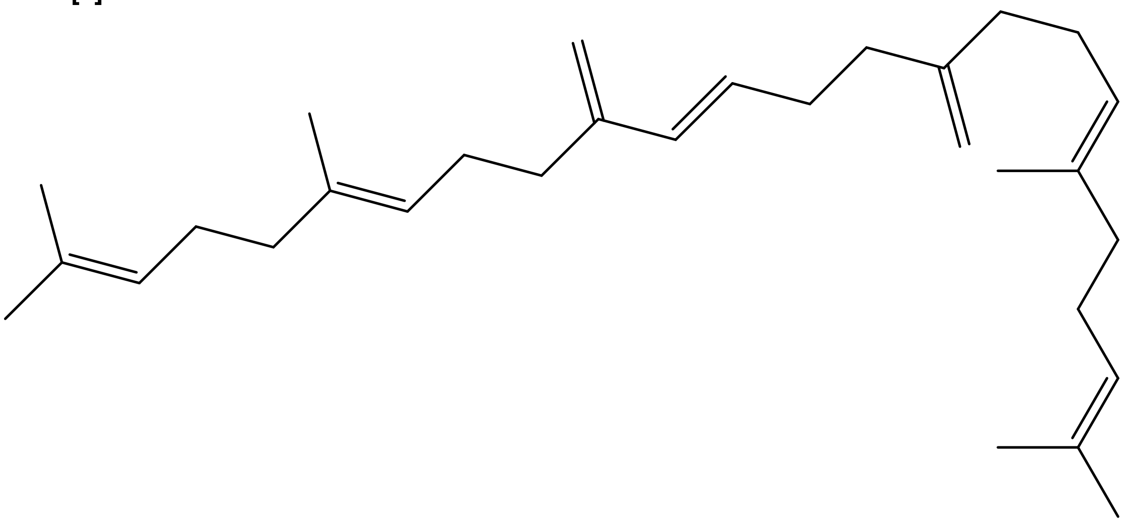

Squalane [11]

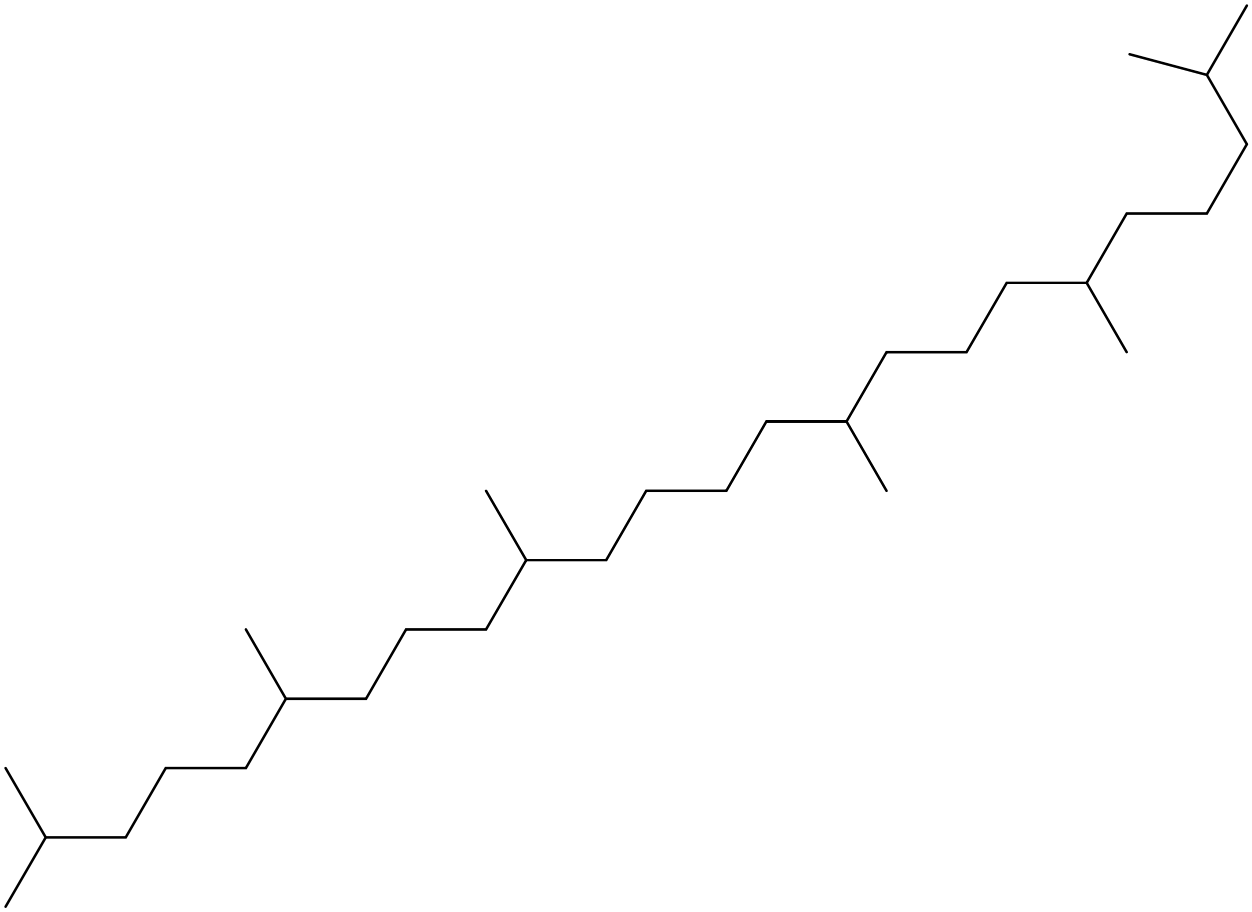

Farnesene thermal dimers, four isomers [3, 4, 5, 6]

3

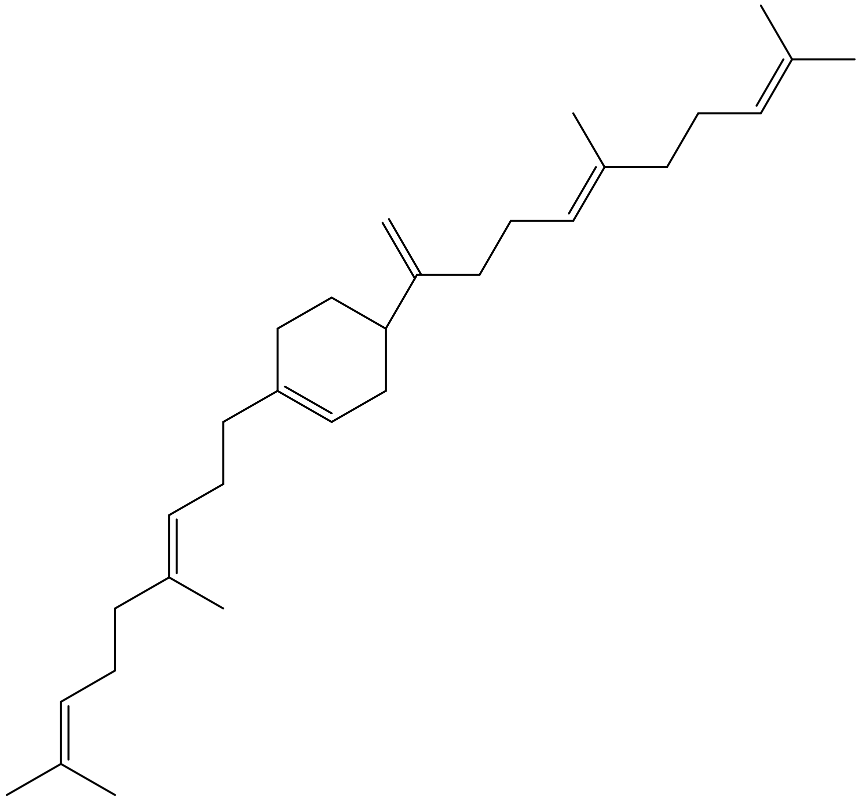

4

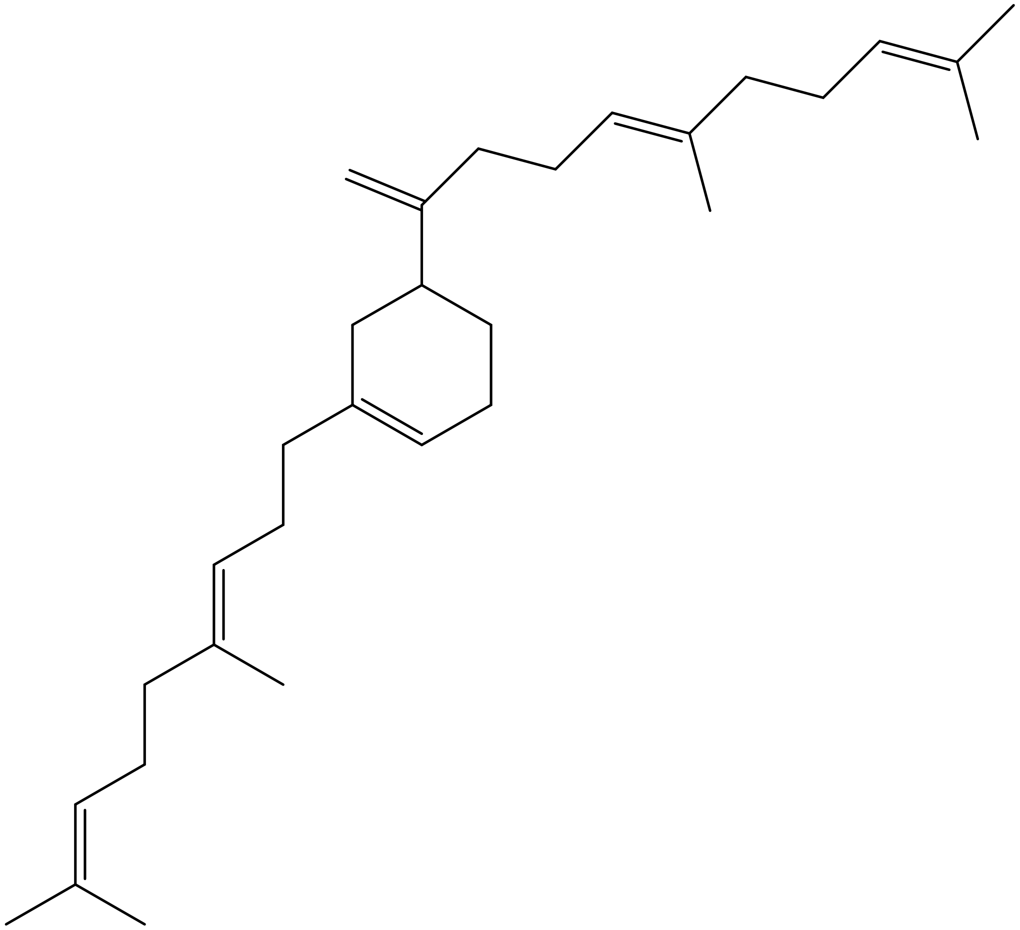

5

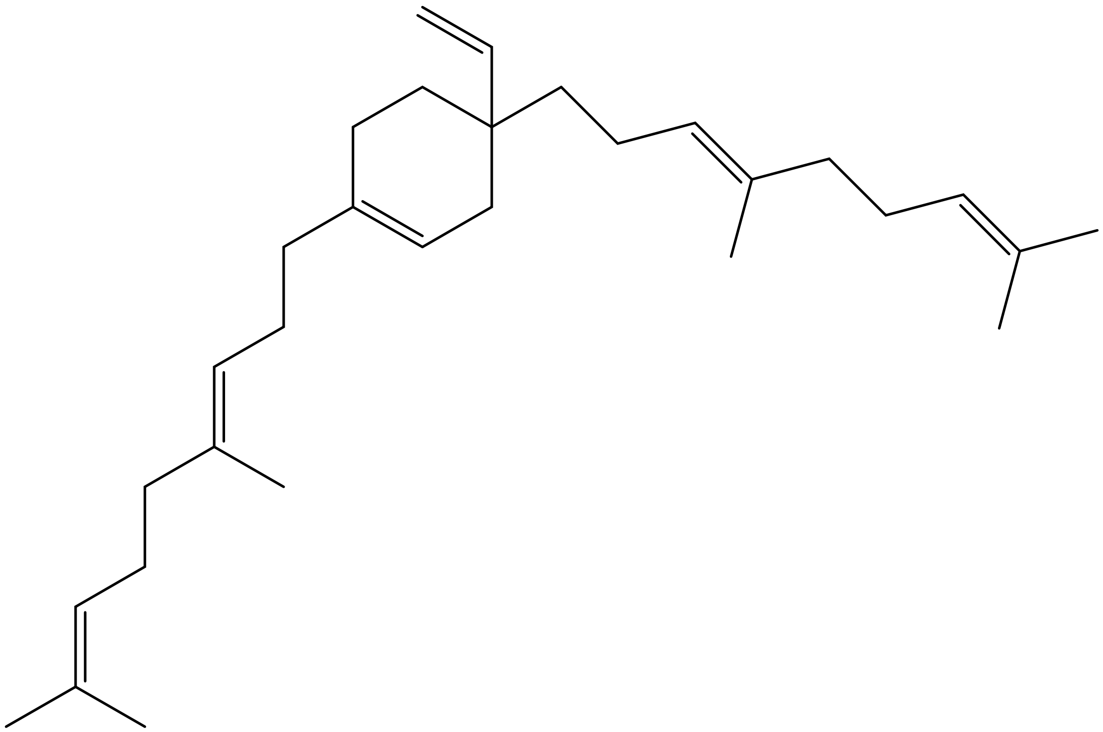

6

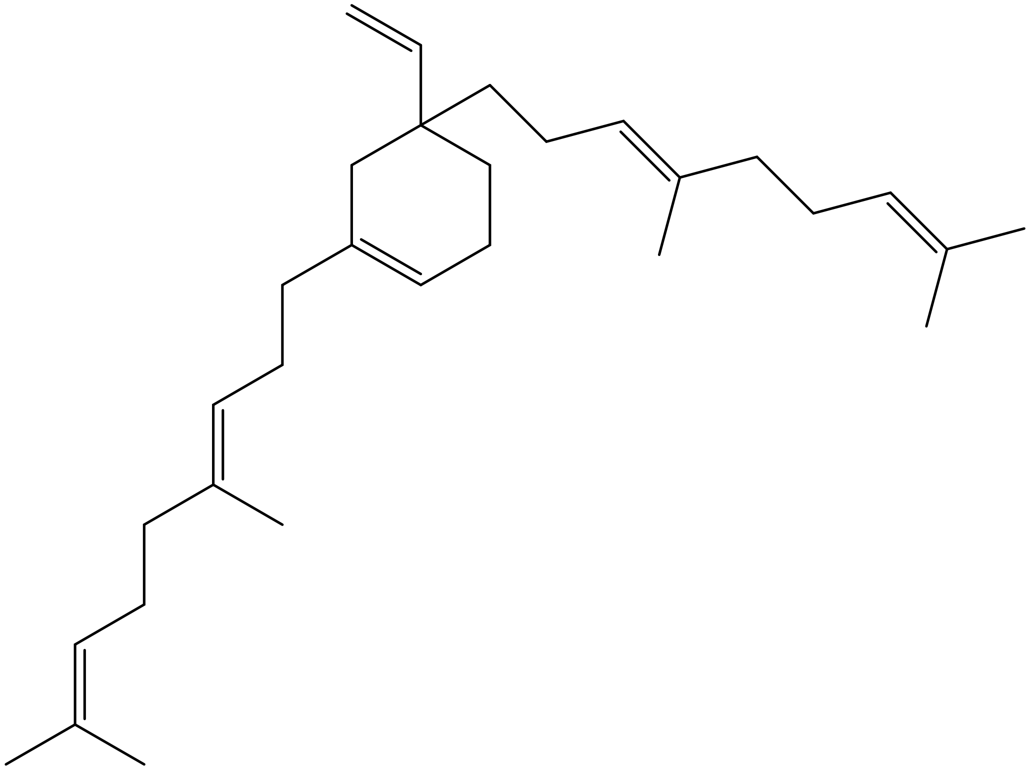

C_{20} dimer (myrcene linear dimer) [7]

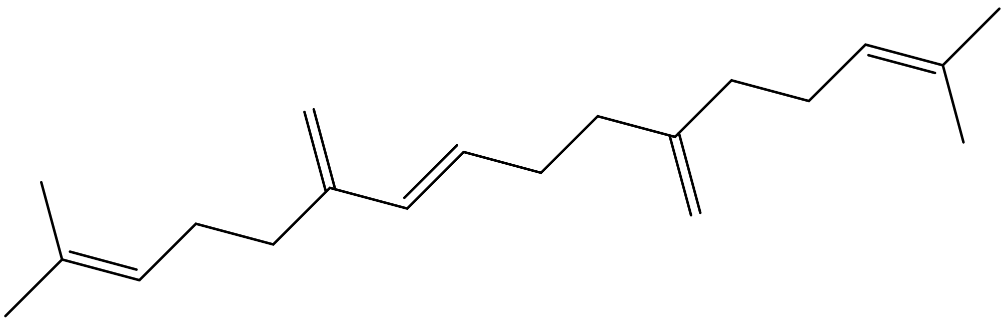

Myrcene (159.4 g, 1.17 mol) was added to a 1-L three-neck flask and diluted with 300 mL of 2-propanol, and palladium(II) acetylacetonate (1.13 g) and triphenylphosphine (1.56 g) were added. The mixture was heated to 80°C for 16 h. The solvent was removed by rotary evaporation; crude weight was 156.9 g. The sample was diluted with 200 mL of hexanes and filtered through a 6-in-tall by 10-cm-diameter silica-gel column and then eluted with 500 mL of n-pentane and then 250 mL of 10% ethyl acetate in hexanes. The combined filtrates were concentrated by rotary evaporation to give 148.8 g of nearly colorless oil. Additional evaporation and first-stage vacuum distillation at 160-170°C and 0.35 torr gave 120.2 g of slightly impure product, which contained some C10 impurities. The product was resubjected to distillation at 120°C and 0.5 torr to give 5.9 g of light materials (C10 starting material) and 112 g of desired product. Yield was 70%.

Proton (1H) nuclear magnetic resonance (NMR) (in ppm): 6.08 (d, J = 15.8 Hz, 1H), 5.77 (dt, J = 15.6, 6.8 Hz, 1H), 5.14 (m, 2H), 4.88 (broad doublet, J = 11.1 Hz, 2H), 4.74 (broad singlet, 2H), 2.26-2.00 (m, 12H), 1.69 (broad singlet, 6H), 1.61 (broad singlet, 6H).

Carbon 13 (13C) NMR (in ppm): 148.98, 148.07, 132.26, 131.63, 131.58, 129.55, 124.32, 124.18, 36.16, 35.99, 32.35, 31.24, 26.98, 26.46, 25.69, 17.71.

C_{25} dimer (farnesene and myrcene coupling product) [8]

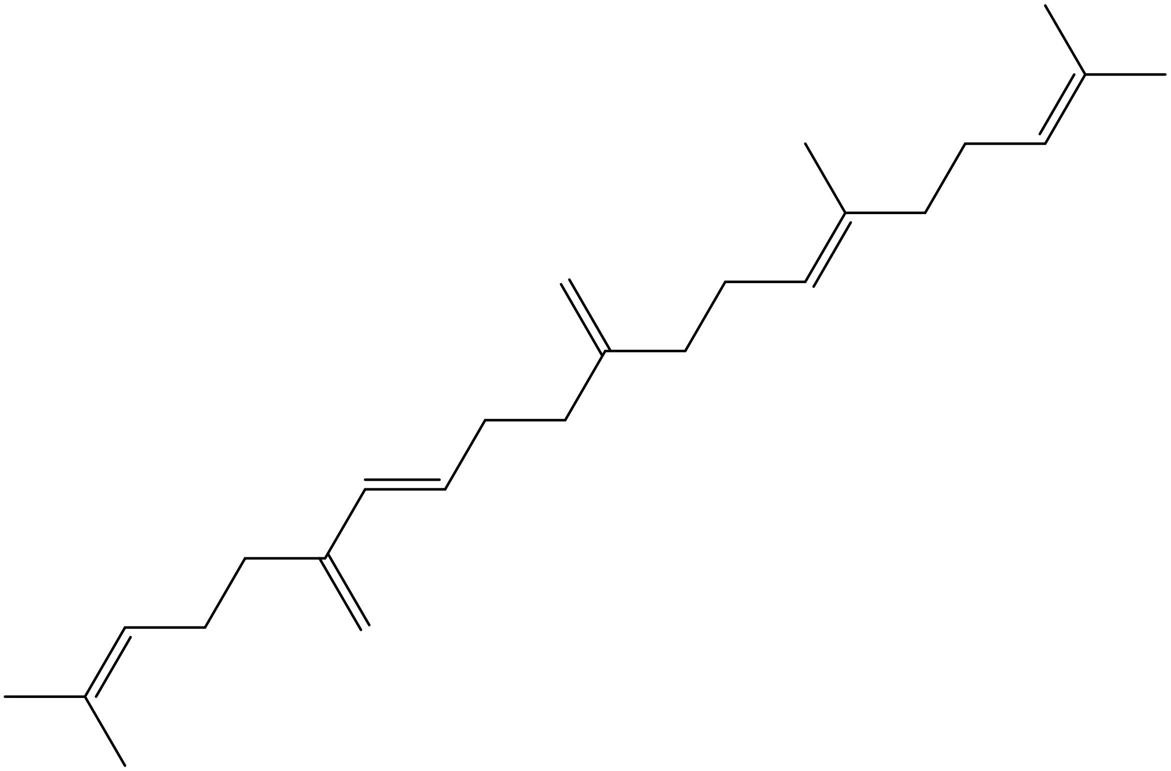

Farnesene (79.8 g, 0.39 mol) was added to myrcene (154 g, 1.13 mol), and the headspace was purged with nitrogen. 400 mL of 2-propanol were added, followed by 1.09 g of palladium(II) acetylacetonate and 1.43 g of triphenylphosphine. The mixture was heated at 80°C for 17 h. Gas chromatography (GC) showed a mixture of C25, C20, and C30 coupling products. Most of the solvent was removed by rotary evaporation. The crude product was diluted with 600 mL of 10% ethyl acetate in hexanes and filtered through silica gel. The filter cake was washed with 400 mL of 10% ethyl acetate and concentrated to

give 224.2 g of black liquid. 111.3 g of this black oil were diluted with 200 mL of hexanes and filtered through silica gel, and the silica gel was washed with 200 mL of hexanes and then 200 mL of 10% ethyl acetate in hexanes. This process was repeated with the rest of the batch, and the combined filtrates were evaporated, combined, and concentrated on the rotary evaporator to give 213.1 g of nearly colorless liquid. Distillation at 160°C and 0.35 torr gave a light fraction of mainly C20s and a distillation residue of 167.1 g, which was enriched in C25s and C30s. Further distillation at 160°C and 0.35 torr gave a light fraction weighing 30.6 g, which was approximately a 4:1 mixture of C20:C25 materials and was discarded. The distillation residue weighed 136.1 g, which was further distilled to give a light fraction that weighed 31.9 g and was a mixture of approximately 70% C20s and 30% C25s. The nonvolatile residue from the distillation was further distilled at 200°C and 0.54 torr to give a fraction that weighed 44 g and was approximately 80% C25 products along with some C20s and C30 impurities. This material was submitted for initial formulation and screening.

1H NMR: 6.08 (d, J = 15.8 Hz, 1H), 5.73 (broad doublet of triplets, J = 15.8, 6.6 Hz, 1H), 5.18-5.07 (m, 3H), 4.88 (d, J = 11.5 Hz, 2H), 4.74 (s, 2H), 2.28-1.95 (m, 16H), 1.68 (broad singlet, 6H), 1.61 (broad singlet, 6H).

13C NMR: 148.99, 148.98, 146.07, 146.03, 135.26, 135.21, 132.26, 132.24, 131.64, 131.59, 131.28, 129.56, 129.53, 124.40, 124.39, 124.31, 124.17, 124.06, 113.37, 109.26, 109.22, 39.73, 36.15, 35.98, 32.34, 31.24, 26.97, 26.84, 26.75, 26.74, 26.45, 26.36, 25.70, 17.71, 17.70, 16.04.

Alcohols A, racemic [10]

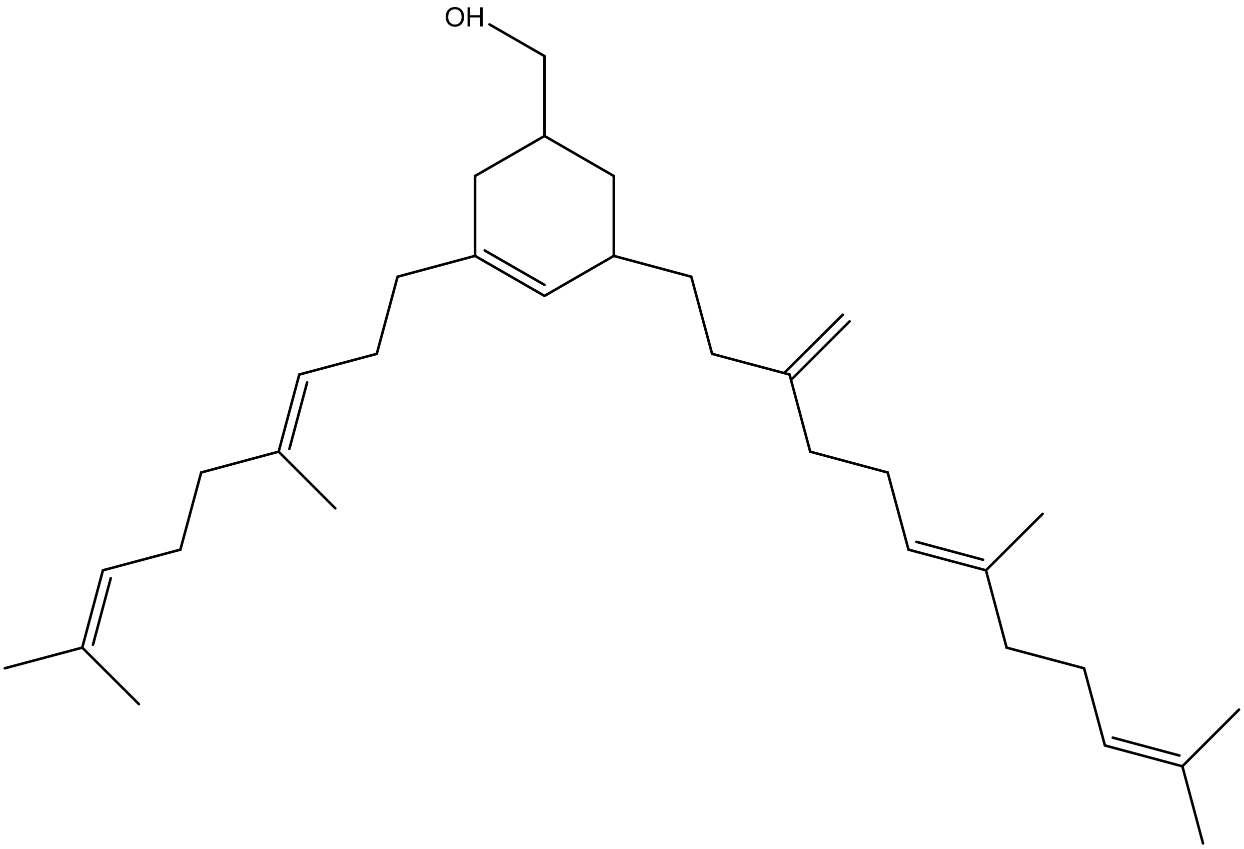

10, cis and trans, racemic

Batch 1

90 mL of dry tetrahydrofuran (THF) were cooled in an ice-water bath, and solid lithium aluminum hydride (0.98 g, 25.8 mmol) was added, followed by dropwise addition of a solution of the esters (19.8 g, 36.9 mmol theoretical from batch 2) in 60 mL of THF. After 1 h at room temperature, thin-layer chromatography (TLC) showed no starting ester, so the mixture was cooled to 0°C, and 5 mL of water were added slowly (hydrogen released). Then 55 mL of 0.50 M hydrochloric acid were added. The phases were separated, and the organic phase was concentrated to give 18.3 g of a nearly colorless oil.

Batch 2

Lithium aluminum hydride (2.99 g, 79 mmol) was added to 250 mL of dry THF cooled in an ice-water bath. A solution of the esters (60.9 g of batches 1 and 2, up to 0.113 mol) in 100 mL of THF was added dropwise. After the addition was complete, the mixture was stirred at room temperature for 2 h. The flask was re-cooled to 0ºC, and 20 mL of water were added slowly followed by 100 mL of 5% aqueous hydrochloric acid. Most of the THF was removed by rotary evaporation, and the phases were separated. The aqueous phase was extracted with an additional 100 mL of ethyl acetate, and the combined organic phases were concentrated to give 61.2 g of crude alcohol. The two batches were purified by silica-gel chromatography using a 5% ethyl acetate to 30% ethyl acetate step gradient to give 44.9 g of desired alcohols as a 55:45 mixture of isomers. Yield was 49.6% over the two steps.

1H NMR: 5.41 (broad singlet, 1H for one isomer), 5.31 (broad singlet, 1H for one isomer), 5.14-5.08 (multiplet, 4H for each isomer), 4.73 (broad singlet, 2H for each isomer), 3.64 (dt J = 10.5(t), d coupling hard to assign due to incomplete resolution, 2H for one isomer), 3.55-3.45 (multiplet, 2H for one isomer), 2.35-1.85 (multiplet, 21H for each isomer), 1.68 (broad singlet, 6H for each isomer), 1.60 (broad singlet, 12H for each isomer), 1.48 (multiplet, 2H for each isomer), 1.27 (multiplet, 2H for each isomer), 0.88 (t, J = 7, 1H for each isomer).

13C NMR: 149.91, 149.90, 137.52, 137.34, 135.18, 135.00, 131.24, 124.54, 124.41, 124.20, 124.15, 124.11, 108.87, 65.48, 63.54, 39.76, 39.73, 39.13, 37.87, 37.75, 36.16, 36.10, 35.83, 33.91, 33.25, 32.92, 31.92, 29.52, 29.06, 27.22, 22.46, 17.69, 16.05, 16.03, 14.14.

M/Z = 496.5, 496.5

Difarnesyl ether [12]

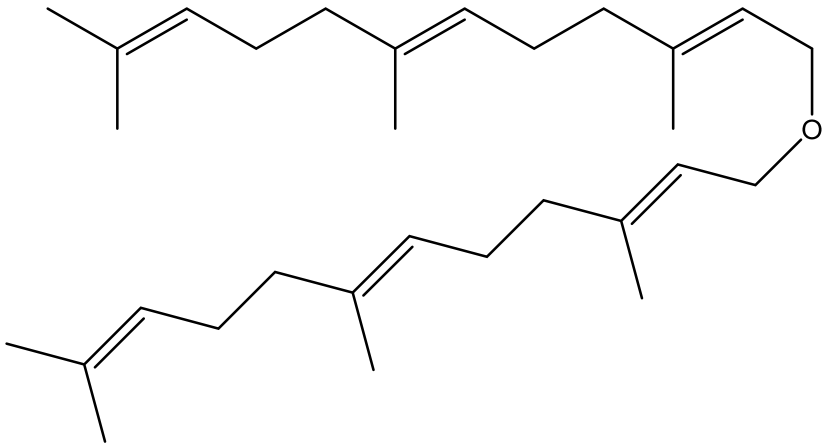

A solution of E,E-farnesol (4.1 g, 18.47 mmol) in 10 mL of THF was added to a solution of potassium tert-butoxide (3.96 g, 35.3 mmol) in 38 mL of THF. The solution turned light yellow. After 15 min, a solution of farnesyl chloride in 6 mL of THF was added. The mixture was heated at 56ºC for 90 min and cooled to room temperature. Most of the solvent was removed by rotary evaporation. 25 mL of 5% aqueous sodium bicarbonate and 25 mL of deionized water were added, and the crude product was evaporated to remove ethyl acetate and purified by silica-gel chromatography using 2% ethyl acetate in heptanes to give 0.9 g of cleaner fractions (91% by gas chromatography mass spectrometry (GCMS)) and 3.1 g of less pure fractions (85% by GCMS). Estimated yield was 47% (unoptimized).

1H NMR: 5.37 (2H, dt, J = 6.83 Hz (triplet), J = 1.2 Hz (doublet)), 5.10 (4H, m), 3.97 (4H, d, J = 6.6 Hz), 2.16-2.01 (m, 12H), 2.00-1.93 (m, 4H), 1.67 (broad singlet, 12H), 1.59 (broad singlet, 12H).

13C NMR: 139.99, 135.22, 131.26, 124.36, 123.93, 121.11, 66.40, 39.70, 39.62, 26.73, 26.34, 25.66, 17.65, 16.46, 15.97.

M/Z = 426.4

Esters C, racemic (intermediate for alcohols C) [21]

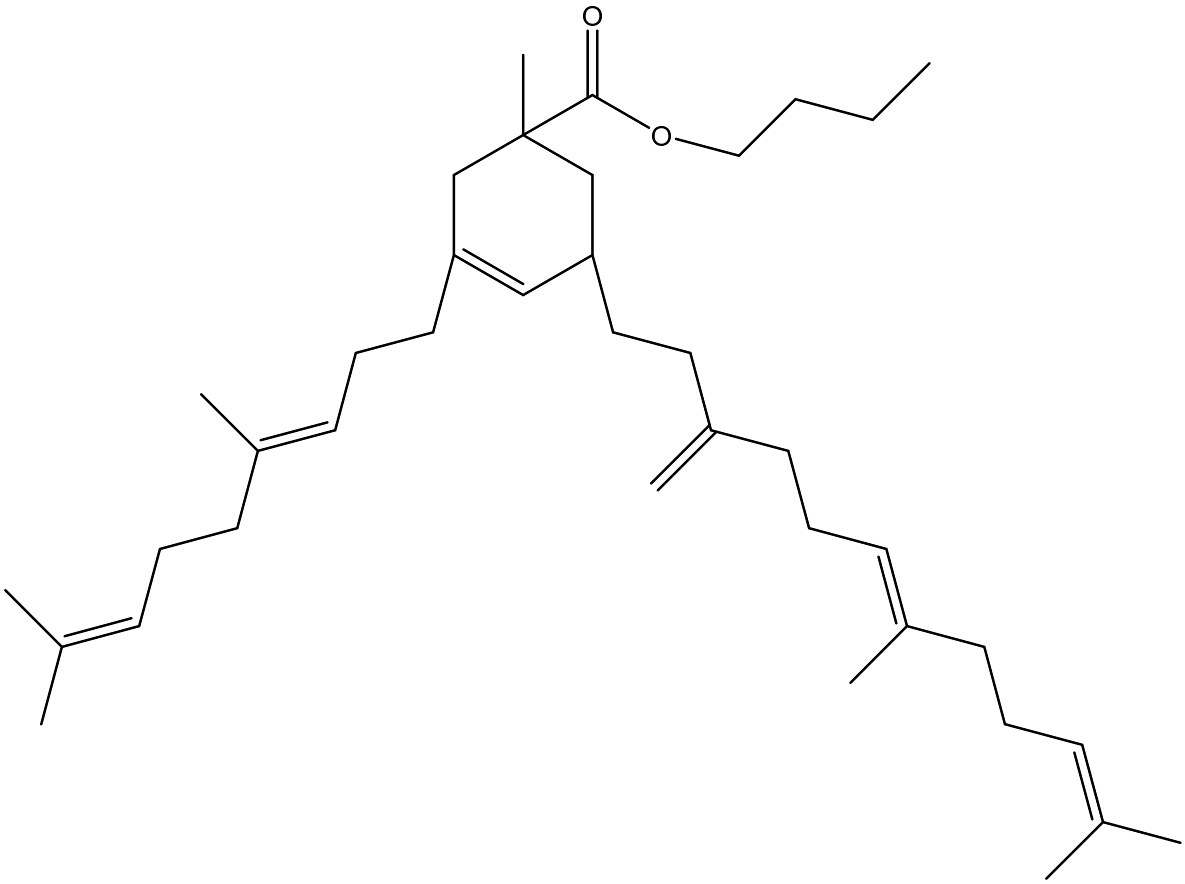

Racemic, mixture of two stereoisomers

DHIS (14.3 g, 35 mmol) was added to a 250-mL flask followed by n-butyl methacrylate (7 mL, 44 mmol) and 15 mL of toluene. The mixture was heated at 120°C for 8 h, when GC showed an approximate 2:1 ratio of desired product isomers to DHIS. An additional 10 mL of toluene and 2 mL of n-butyl methacrylate were added, and heating at 120°C was resumed for 5 h. GC showed high (but incomplete) conversion. The reaction product was concentrated by rotary evaporation, and most of the starting n-butyl methacrylate was removed by distillation under vacuum at approximately 1 torr and 80°C to give 17.8 g of slightly cloudy yellow oil. 1.2 g of this oil were purified by silica-gel chromatography using a 10% toluene/heptane to 10% ethyl acetate/heptane step gradient to give 0.6 g of colorless oil. Yield was estimated as 46% of a mixture of two isomers.

1H NMR: 5.447 (4, J = 4.9 Hz, 1H for one isomer), 5.30 (broad singlet, 1H for one isomer), 5.10-5.06 (m, 4H for each isomer), 4.719 (d, J = 4.3 Hz, 2H for each isomer), 4.16-4.04 (m, 2H for each isomer), 2.3-1.85 (m, 21H for each isomer), 1.687 (singlet, 6H for each isomer), 1.608 (s, 12H for each isomer), 1.70-1.57 (m, 2H for each isomer), 1.50-1.30 (m, 6H for each isomer), 1.18 (3, 3H for each isomer), 1.03 (s, 3H for one isomer), 0.937 (t, J = 7.4 Hz, 3H for each isomer).

13C NMR: 177.77, 149.57, 135.86, 135.14, 135.12, 131.31, 124.36, 124.06, 122.97, 122.31, 108.98, 64.20, 63.96, 44.46, 42.24, 40.41, 39.74, 39.72, 37.48, 36.06, 35.89, 34.01, 32.74, 31.94, 30.72, 29.77, 26.78, 26.72, 26.39, 26.28, 25.71, 25.45, 22.40, 19.30, 19.25, 17.70, 16.08, 16.07, 16.02, 13.75.

M/Z = 550.5, 550.5

Alcohols C, racemic [13]

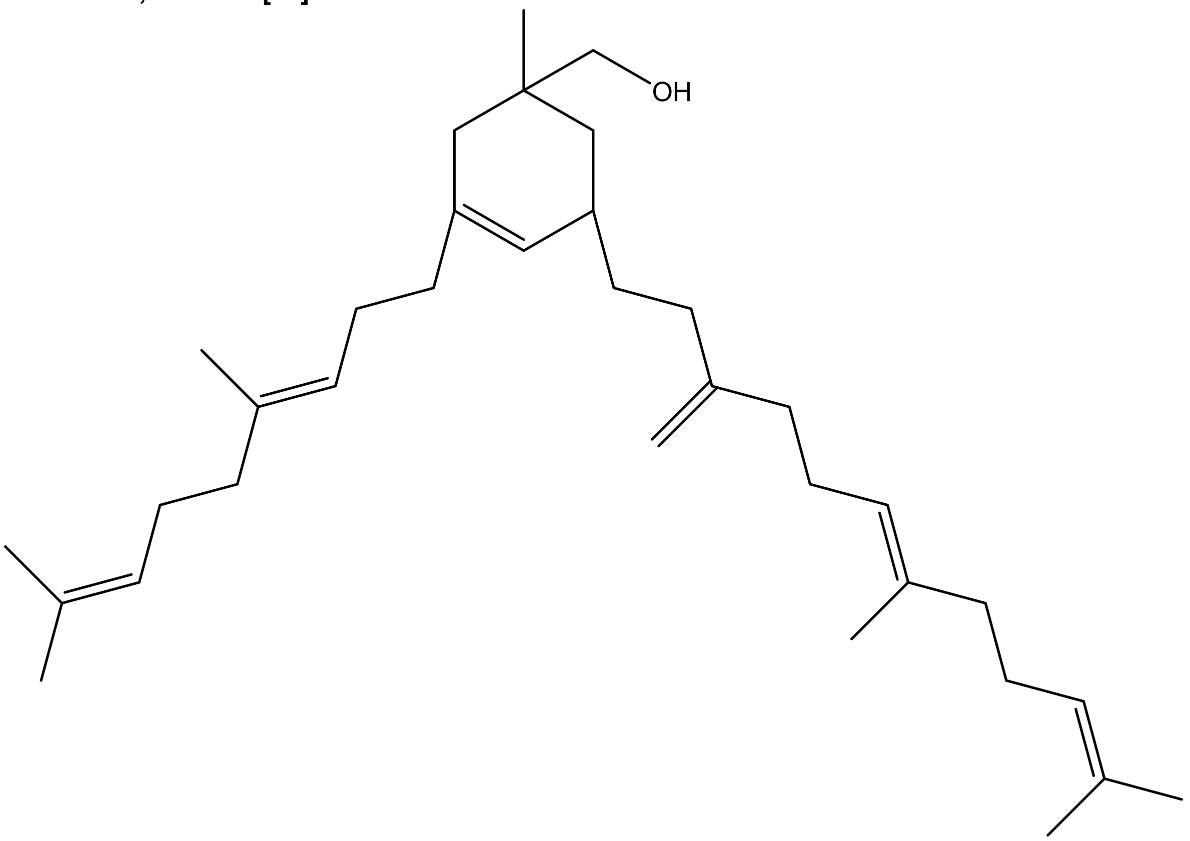

Cis and trans, racemic

Impure esters C (16.6 g, approximately 50% pure, 18.1 mmol) were dissolved in 20 mL of THF and added to a 0°C suspension of lithium aluminum hydride (0.545 g, 15.1 mmol) in 20 mL of dry THF over 10 min. Ten min after the addition was complete, the suspension was stirred at room temperature for 90 min. It was cooled back to 0°C, and 1 mL of water (hydrogen liberated) was carefully added. The mixture was then carefully acidified to pH 4 with 5% aqueous hydrochloric acid, and the product was extracted with 25 mL of ethyl acetate. After removal of most of the solvent by rotary evaporation, the alcohols were purified by silica-gel chromatography using 10% ethyl acetate as eluent. Yield was 5.5 g of colorless oil (76%).

1H NMR: 5.359 (broad singlet, 1H for one isomer), 5.322 (broad singlet, 1H for one isomer), 5.16-5.06 (m, 4H for both isomers), 4.737 (broad singlet, 2H for both isomers), 3.49 (d, J = 18.8 Hz, 1H for one isomer), 3.433 (s, 2H for one isomer), 3.34 (d, J = 18.8 Hz, 1H for one isomer), 2.26-1.78 (overlapping multiplets, 21H per isomer), 1.679 (broad singlet, 6H for each isomer), 1.600 (broad singlet, 12H for each isomer), 1.35-1.20 (m, 2H for one isomer, 1H for one isomer), 1.18-1.04 (m, 1H for one isomer), 0.97 (s, 3H for one isomer), 0.881 (dd (overlapping), J = 6.8 Hz, 2H for each isomer), 0.76 (s, 3H for one isomer).

13C NMR: 150.03, 149.81,136.75, 136.12, 135.18, 135.15, 135.03, 134.99, 131.25, 124.39, 124.37, 124.19, 124.16, 124.09, 123.36, 123.20, 108.99, 108.96, 70.34, 67.37, 42.72, 39.74, 39.71, 38.68, 37.63, 37.61, 36.47, 36.29, 36.15, 36.08, 34.41, 34.25, 31.88, 30.66, 29.74, 29.02, 28.61, 28.46, 26.78, 26.72, 26.48, 26.46, 26.36, 25.68, 25.58, 22.69, 17.68, 17.27, 16.06, 16.01, 14.10.

M/Z = 480.4, 480.4

Aldehydes A [14]

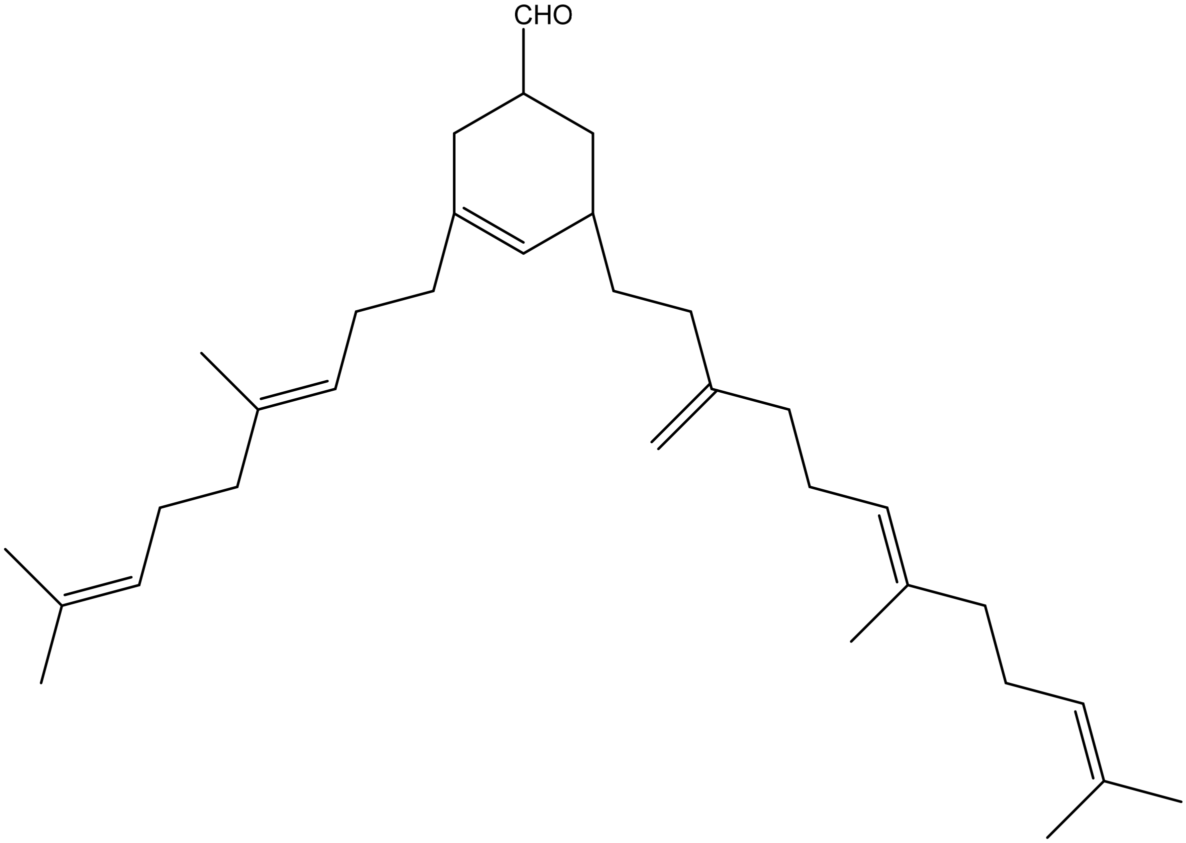

Cis and trans, racemic

Dilute alcohols A (4.2 g, 9 mmol) in 100 mL of methylene chloride were cooled in an ice-water bath. 4 mL of dimethyl sulfoxide and 7 mL of triethylamine were added followed by sulfur trioxide pyridine complex (5.4 g, 34 mmol). After 1 h, TLC showed low conversion, so an additional 7 mL of triethylamine were added followed by 3 mL of dimethyl sulfoxide and sulfur trioxide pyridine complex (5.6 g, 35.2 mmol). After an additional hour stirring, TLC showed high conversion, so most of the solvent was removed by rotary evaporation. The residue was combined with 100 mL of ethyl acetate and 100 mL of water, and the organic phase was separated and concentrated to give 10.3 g of a two-phase mixture. The lighter phase was purified using a 4% ethyl acetate/heptanes to 8% ethyl acetate/heptanes step gradient to give 3.8 g of aldehydes A (90.9%) as a colorless oil, which was a mixture of two diastereoisomers.

1H NMR: 9.787 (d, J = 1.5, 1H for one compound), 9.634 (d, J = 2.2, 1H for one compound), 5.503 (broad singlet, 1H for one compound), 5.380 (broad singlet, 1H for one compound), 5.116-5.05 (M, 4H for each compound), 4.726 (broad singlet, 2H for each compound), 2.60-2.40 (m, 2H for one compound, 1H for the other compound), 2.27-1.95 (M, 41H for each compound), 1.92-1.82 (m, 2H for one compound, 1H for one compound), 1.672 (broad singlet, 6H for each compound), 1.594 (broad singlet, 12H for each compound), 1.59-1.40 (m, 2H for each compound).

13C NMR: 205.53, 204.91, 149.20, 149.00, 138.24, 137.75, 135.22, 135.21, 135.19, 131.26, 131.23, 131.22, 124.36, 124.35, 124.32, 123.98, 123.95, 123.83, 123.66, 123.06, 109.37, 109.22, 51.02, 49.88, 39.72, 39.71, 37.69, 37.58, 36.04, 35.92, 35.08, 34.11, 33.78, 33.17, 33.01, 30.99, 26.76, 26.71, 26.48, 26.34, 26.31, 26.29, 25.71, 25.69, 21.56, 20.92, 17.68, 16.04, 16.02.

M/Z = 464.4, 464.4

Compounds 1 [15]

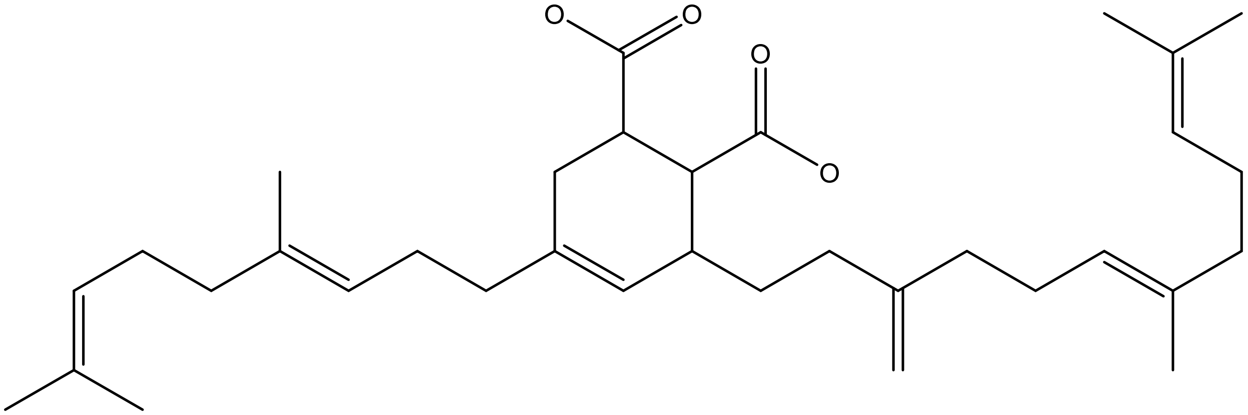

Cis and trans, racemic

50 mL of N,N-dimethyl were added to DHIS (51.9 g, .127 mol) followed by fumaric acid (13.95 g, 0.120 mol) under a nitrogen atmosphere. The mixture was heated at 120ºC for 27 h. The crude NMR looked okay with some starting material. The solvent was removed by distillation at 60-80ºC and 0.2 torr to give 67.3 g of light brown oil. Silica-gel chromatography using 5% methanol in methylene chloride and 2% acetic acid gave 7.2 g of cleaner fractions (11%, 85% pure) along with 41.5 g of mixed fractions, which were 70-80% pure. Estimated yield was 59%.

1H NMR: 5.57 (broad singlet, 1H for one compound), 5.33 (broad singlet, 1H for each compound), 5.09 (m, 4H for each compound), 4.73 (broad singlet, 2H for each compound), 3.10-2.85 (m, 2H for one compound, 1H for one compound), 2.65-2.55 (m, 1H for one compound), 2.52-2.42 (m, 1H for each compound), 2.40-2.33 (m, 1H for each compound), 2.25-1.92 (m, 19H for each compound), 1.676 (broad singlet, 6H for each compound), 1.598 (broad singlet, 12H for each compound), 1.6-1.45 (m, 1H for each compound), 1.42-1.24 (m, 1H for each compound).

13C NMR: 182.74, 182.03, 181.62, 181.06, 149.30, 148.97, 135.85, 135.63, 135.34, 135.21, 135.17, 131.32, 131.23, 131.21, 124.39, 124.36, 124.27, 124.02, 123.96, 123.51, 123.47, 123.23, 109.32, 109.18, 46.84, 46.17, 43.39, 39.71, 38.23, 38.19, 37.10, 31.64, 31.26, 31.12, 30.53, 26.73, 26.31, 26.27, 26.24, 26.15, 25.67, 17.67, 16.07, 16.00, 15.99.

M/Z = 523.38, 523.38 (negative ion, M-1)

Acids A [16]

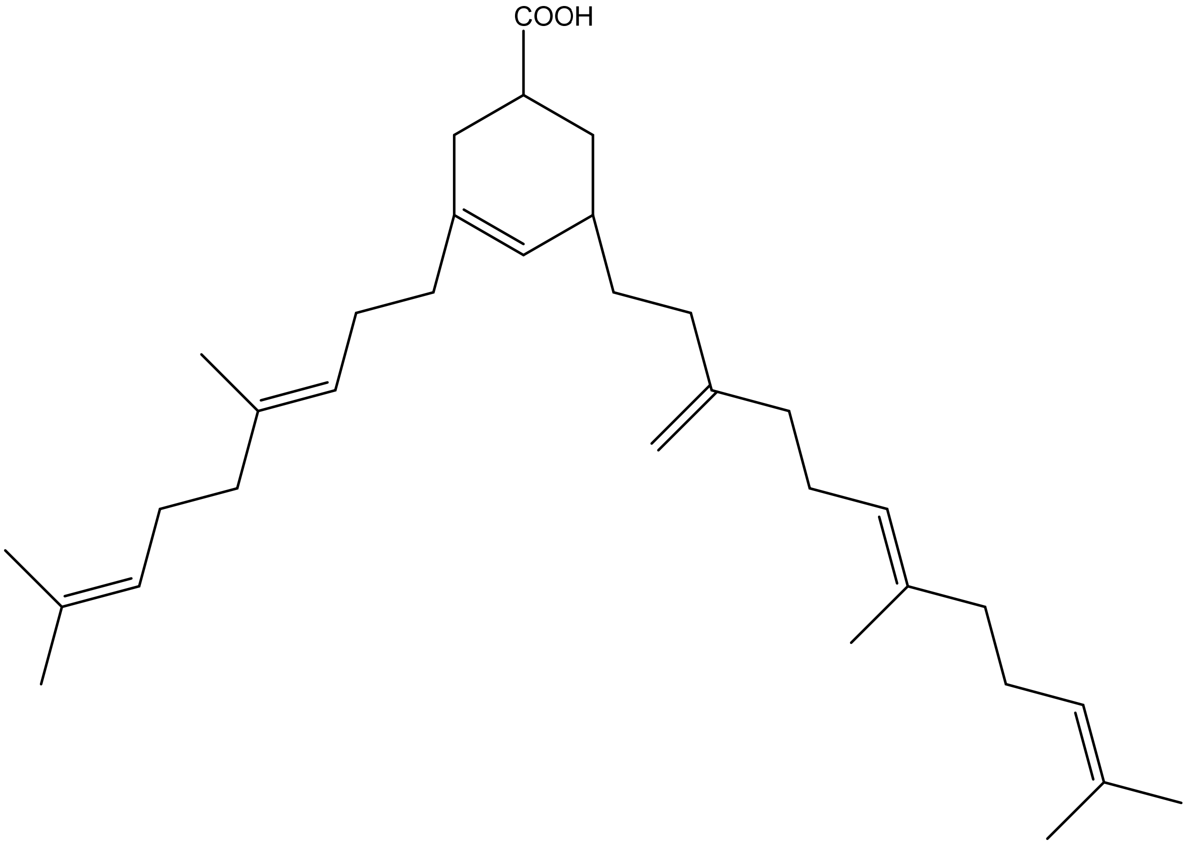

Cis and trans, racemic

A solution of potassium hydroxide (8.34 g, 0.149 mol) in 100 mL of methanol was prepared. Esters A (7.5 g, 14 mmol) were added as a two-phase mixture with 90 mL of methanol and heated at reflux for 16 h. Most of the solvent was removed by rotary evaporation. Thirty mL of water were added, and the mixture was acidified to a methyl orange endpoint with 5% aqueous hydrochloric acid while cooling with an ice-water bath. The product was extracted with 70 mL of ethyl acetate, and the aqueous phase was extracted with 30 mL of ethyl acetate. The combined organic phases were concentrated on the rotary evaporator to give 7.2 g of crude acids as an orange oil. The oil was purified by silica-gel chromatography using a 10% ethyl acetate/heptanes and 2% acetic acid to a 20% ethyl acetate/heptanes and 2% acetic acid step gradient to give 5.6 g of acids A as a light yellow oil.

1H NMR: 5.52 (broad doublet, J = 4.7, 1H for one isomer), 5.37 (broad singlet, 1H for one isomer), 5.17-5.06 (m, 4H for each isomer), 4.737 (broad singlet, 2H for each isomer), 2.72-2.65 (m, 1H for each isomer), 2.49 (broad singlet, 1H for one compound), 2.33-2.2.27 (broad triplet, 1H for one compound), 2.23-1.98 (23H for one isomer, 22H for the other isomer), 1.97-1.73 (m, 1H for each isomer), 1.689 (broad singlet, 6H for each isomer), 1.609 (broad singlet, 12H for each isomer), 1.50-1.39 (m, 1H for each compound).

13C NMR: 182.86, 181.50, 149.51, 149.39, 137.50, 137.21, 135.18, 135.16, 135.13, 135.11, 131.25, 131.21, 124.41, 124.37, 124.08, 124.01, 123,97, 123.33, 123.24, 109.11, 108.99, 45.16, 43.61, 39.73, 37.59, 36.79, 36.13, 35.96, 35.43, 33.67, 32.83, 32.63, 27.43, 26.78, 26.74, 26.32, 25.80, 25.70, 17.69, 16.05, 16.00.

M/Z = 479.3864, 479.3864 (M–1, negative ion)

Acids C [17]

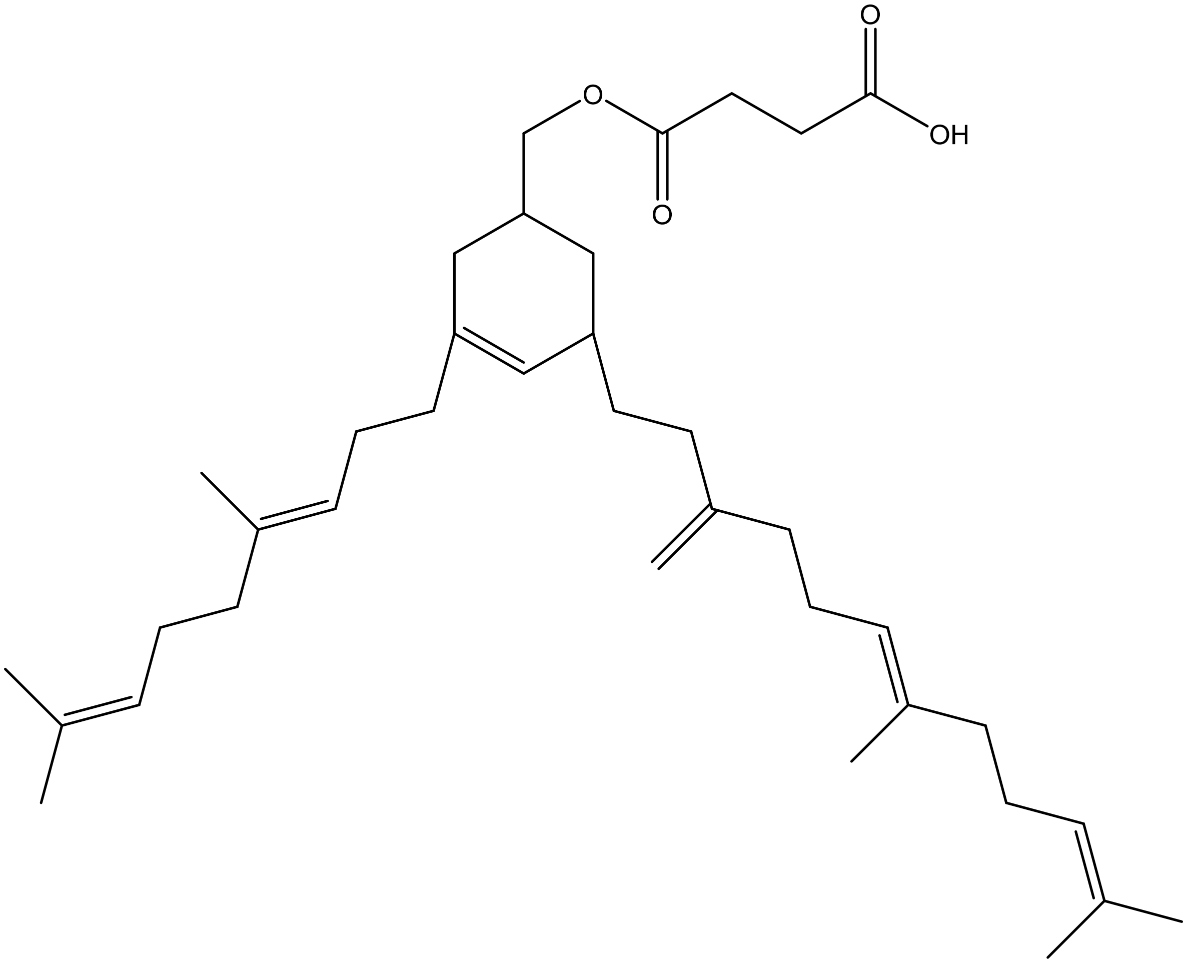

Cis and trans, racemic

80 mL of ethyl acetate were added to alcohols A (8.1 g, 17.38 mmol). Triethylamine (5 mL) was added and cooled in an ice-water bath, and then 4-dimethylaminopyridine (222 mg, catalytic) was added followed by solid succinic anhydride (2.84 g, 28.4 mmol). After 44 h stirring at 25°C, TLC showed high conversion, so the solution was quenched by the addition of 10 mL of water, and the solution was acidified to a methyl orange endpoint with 5% aqueous hydrochloric acid. The organic phase was separated, the aqueous phase was extracted with 20 mL of ethyl acetate, and the combined organic phases were concentrated by rotary evaporation to give 11.1 g of cloudy oil. Silica-gel chromatography using a 20% ethyl acetate/heptanes to 35% ethyl acetate/heptanes and 2% acetic acid to 50% ethyl acetate/heptanes and 2% acetic acid step gradient gave fractions 17 to 26 (7.1 g) of desired product as a colorless oil (72% yield). Fractions 15 and 16 (2 g) were slightly less pure by TLC but gave a good proton NMR.

1H NMR: 5.37 (broad singlet, 1H for one isomer), 5.29 (broad singlet, 1H for one isomer), 5.15-5.07 (m, 4H for both isomers), 4.73 (broad singlet, 2H for both isomers), 4.14-4.07 (m, 2H for one isomer or 1H for each isomer), 4.04-3.97 (m, 2H for one isomer or 1H for each isomer), 2.75-2.58 (m, 4H for each compound), 2.25-1.89 (m, 21H for each compound), 1.83-1.53 (overlapping multiplets, 2H for each compound), 1.677 (broad singlet, 6H for each compound), 1.598 (broad singlet, 12H for each compound), 1.52-1.40 (m, 2H for each compound), 1.35-1.20 (m, 1H for each compound).

13C NMR: 178.37, 172.18, 149.69, 149.58, 137.43, 137.36, 135.17, 135.15, 135.07, 131.24, 124.39, 124.37, 124.12, 124.10, 124.07, 124.05, 123.94, 123.68, 109.00, 108.93, 77.36, 77.04, 76.72, 67.33, 65.41, 39.72, 39.71, 37.79, 37.65, 36.32, 36.11, 36.03, 35.93, 35.32, 33.73, 33.09, 32.73, 29.61, 28.99, 28.90, 28.88, 26.77, 26.73, 26.67, 26.44, 26.39, 26.36, 26.34, 26.26, 23.67, 22.80, 17.68, 16.03, 16.02.

M/Z = 565.4280, 565.4280 (M-1, negative ion)

Esters A, racemic [18]

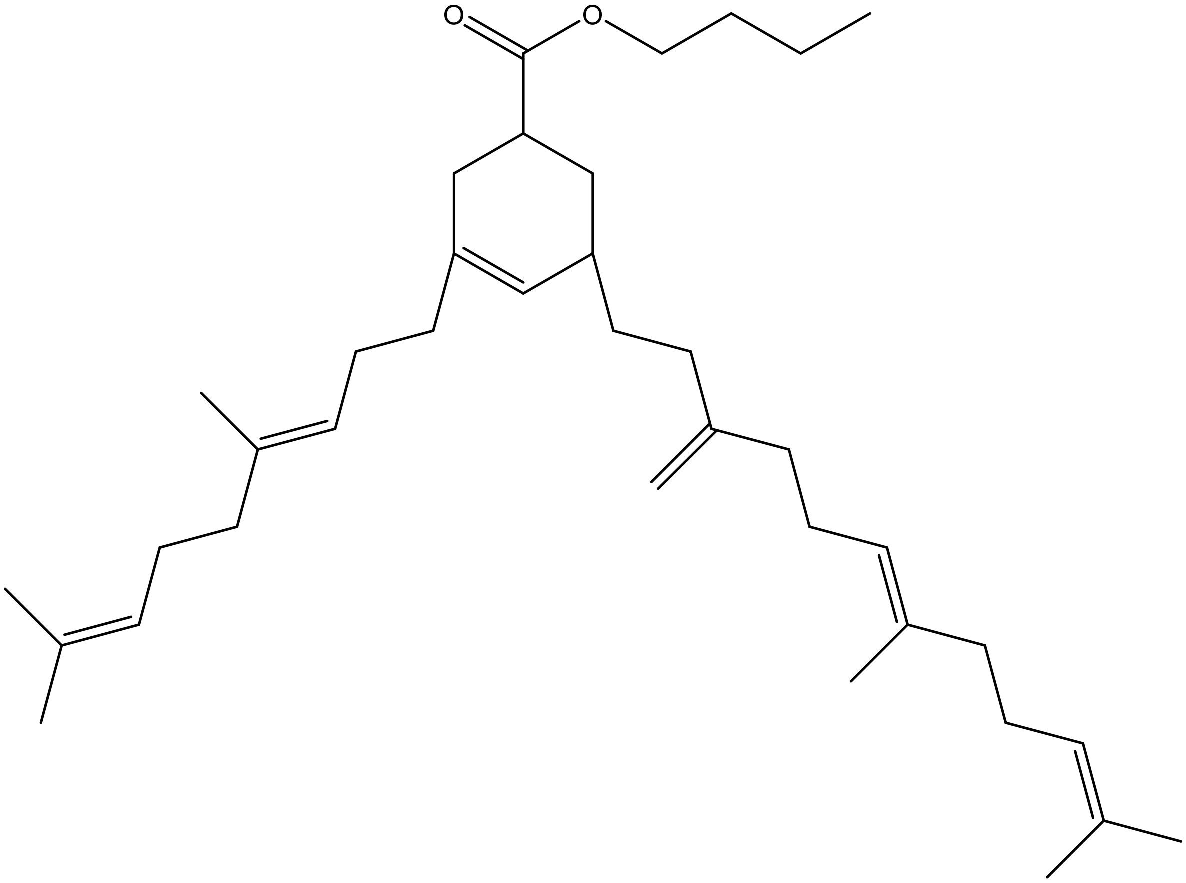

Cis and trans, racemic

Batch 1

DHIS (9.2 g, 22.5 mmol), 6 mL of xylenes, and butyl acrylate (6 mL, 42.1 mmol) were combined under a nitrogen atmosphere and then heated at 135°C. After 65 min, the reaction was essentially complete by GC. Xylenes were removed by distillation at 65°C and 0.5 torr on a vacuum system (Kugelrohr), and then the desired product was purified by silica-gel chromatography using 10% ethyl acetate as eluent. Yield was 12.6 g of slightly impure material containing some residual solvent and hydrocarbon impurities.

Batch 2

DHIS (69.8 g, 0.171 mol), butyl acrylate (35 mL, 0.245 mol), and 50 mL of toluene were combined and heated under a nitrogen atmosphere at 111-120°C for 6 h. GC showed that conversion was very high. Most of the solvent was removed by rotary evaporation. Crude weight was 109.8 g (91.66 g theoretical). 40.2 g of this material were purified by silica-gel chromatography using 10% toluene in heptanes to give 7.8 g (fractions 6 to 8, impure product) along with 19.8 g of clean material (fractions 9 to 26). 1.7 g of the mixed fractions were later purified by silica-gel chromatography using 10% toluene in heptanes to give 1.5 g of fractions 12 to 18 used for characterization of the intermediate. It turned out that it was easier to purify the alcohols after the next step, so most of the ester mixture was carried forward as the technical product.

1H NMR: 5.50 (broad singlet, 1H for one isomer), 5.32 (broad singlet, 1H for the other isomer), 5.10 (multiplet, 4H for each isomer), 4.71 (broad singlet, 2H for each isomer), 4.09 (multiplet, 2H for each isomer), 2.62 (dddd, J = 11.4, 10.6, 5.4, and 3.4 Hz, 1H for one isomer), 2.46 (multiplet, 1H for each isomer), 2.24 (ddt, J = 10 for triplet, 3 and 2.3 Hz for the two doublets, 1H for one isomer), 2.2-1.9 (multiplet, 20H for both isomers), 1.67 (broad singlet, 6H for each isomer), 1.60 (broad singlet, 12H for each isomer), 1.43-1.34 (m, 4H for one isomer, 3H for the other isomer), 1.32-1.24 (multiplet, 4H for each isomer), 1.14-1.10 (t, J = 14, 1H for one isomer), 0.93 (t, J = 6.9 Hz, 3H for one isomer), 0.88 (t, J = 7.0 Hz, 3H for one isomer).

13C NMR: 176.22, 174.94, 149.54, 149.45, 137.47, 137.11, 135.10, 131.20, 129.04, 128.24, 124.42, 124.12, 124.07, 123.64, 123.52, 109.09, 108.96, 71.21, 64.09, 63.92, 45.62, 43.78, 39.77, 37.67, 37.65, 37.26, 36.16, 35.96, 35.77, 33.82, 32.93, 32.68, 31.95, 30.83, 30.50, 29.09, 28.14, 27.97, 27.68, 26.83, 26.20, 25.71, 22.76, 20.25, 19.31, 19.25, 19.20, 17.69, 16.06, 16.02, 14.14, 13.74.

M/Z = 552.5 (isomers not resolved by GCMS)

Diesters B, racemic, mixture of two diastereoisomers (intermediate for diols B) [22]

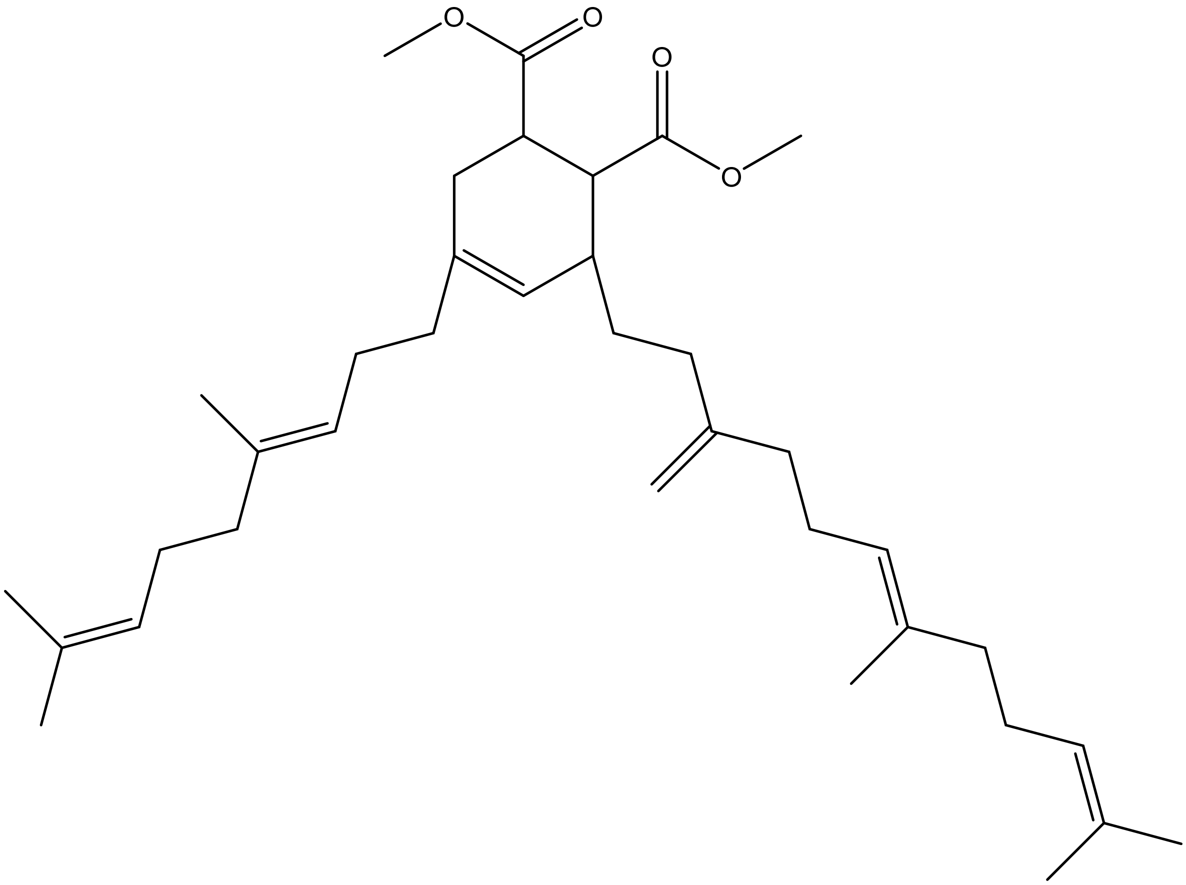

Cis and trans, racemic

DHIS (81.2 g, 0.199 mol) was diluted in 100 mL of toluene, and dimethyl fumarate (24.35 g, 0.169 mol) was added. The mixture was heated at 90ºC for 13 h. The crude product was concentrated by rotary evaporation and vacuum line to give 112.4 g of technical diesters. GCMS showed 93% diesters (not resolved) and approximately 1.5% DHIS. The product was purified by silica-gel chromatography in three batches using 10% ethyl acetate in heptanes as eluent. Yield was 71.4 g and 65%.

1H NMR: 5.546 (d, J = 4.7 Hz, 1H for one isomer), 5.313 (broad singlet, 1H for one isomer), 5.087 (multiplet, 4H for both isomers), 4.714 (d, J = 8.5 Hz, 2H for both isomers), 3.706 (s, 3H for one isomer), 3.701 (s, 3H for one isomer), 3.682 (s, 3H for one isomer), 3.679 (s, 3H for one isomer), 3.05-2.90 (M, 2H for one isomer, 1H for other isomer), 2.60-2.40 (m, 2H for one isomer, 1H for the other isomer), 2.35-2.20 (M, 1H for each isomer), 2.20-1.9 (m, 19H for each isomer), 1.679 (broad singlet, 6H for each isomer), 1.599 (broad singlet, 12H for each isomer), 1.40-1.2 (m, 2H for each isomer).

13C NMR: 176.286, 175.688, 174.944, 174.326, 149.347, 148.980, 135.753, 135.519, 135.421, 135.224, 135.194, 131.347, 131.304, 124.338, 124.324, 124.278, 123.998, 123.950, 123.577, 123.465, 123.212, 109.310, 109.144, 51.92, 51.891, 51.738, 51.607, 47.128, 45.767, 43.137, 39.706, 38.425, 37.894, 37.139, 37.113, 36.044, 35.772, 35.157, 33.501, 32.063, 31.822, 31.692, 31.144, 30.639, 26.744, 26.732, 26.707, 26.265, 26.248, 26.216, 26.178, 25.712, 17.694, 16.088, 16.016.

M/Z = 552.5

Diols B, racemic, mixture of two diastereoisomers [19]

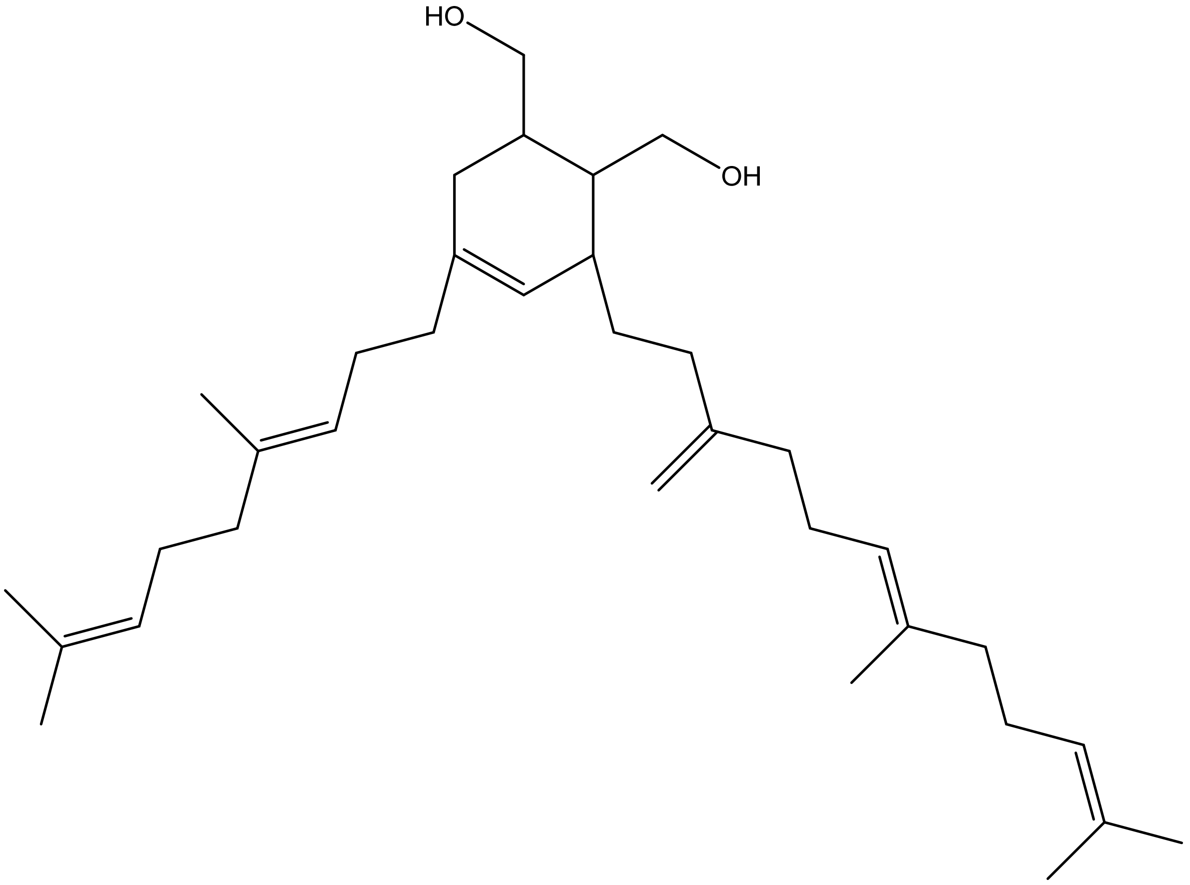

400 mL of THF were cooled in an ice-water bath under nitrogen. After 15 min, solid lithium aluminum hydride (4.9 g, 0.129 mol) was carefully added, and after mixing well, a solution of the diesters B (59.3 g, 0.107 mol) in 200 mL of THF was added over 40 min. The cooling bath was removed, and the mixture was stirred at ambient temperature for 30 min and then cooled in an ice-water bath. Twenty mL of water were added slowly (hydrogen gas was evolved), and the mixture was acidified to pH 2 with 5% aqueous hydrochloric acid and phase separated. The organic phase was concentrated by rotary evaporation to give 163.1 g of water phase and oil. The original aqueous phase was extracted with 200 mL of ethyl acetate. The ethyl acetate from this extraction was combined with the water and oil mixture, and the new mixture was also phase separated. This organic phase was concentrated to give 54.5 g of oil, which was purified by silica-gel chromatography in three batches using a 20% ethyl acetate/heptanes to 50% ethyl acetate step gradient. Yield was 47.6 g and 89.6%.

1H NMR: 5.435 (d, J = 4.2, 1H for one isomer), 5.297 (broad singlet, 1H for one isomer), 5.15-5.07 (m, 4H for each isomer), 4.724 (s, 2H for each isomer), 3.876 (dd, J = 9.3, 2.6, 1H for one isomer), 3.805-3.720 (m, 2H for each isomer), 3.368 (dt, t, J = 11.0, d, J = 6.1, 1H for each isomer), 3.557 (dd, J = 11.2, 4.9, 1H for one isomer), 2.2-1.9 (overlapping multiplets, 21H for each isomer), 1.678 (broad singlet, 6H for each isomer), 1.599 (broad singlet, 12H for each isomer), 1.47-1.35 (multiplet, 2H for each isomer), 1.36-1.25 (multiplet, 2H for each isomer).

13C NMR: 150.01, 149.87, 136.85, 136.79, 135.30, 135.29, 135.23, 135.22, 131.19, 124.51, 124.47, 124.24, 124.15, 124.13, 109.04, 108.94, 66.63, 65.69, 64.66, 63.36, 44.34, 41.84, 40.20, 39.84, 39.82, 37.71, 37.63, 37.01, 36.92, 36.32, 36.16, 35.99, 34.13, 32.70, 32.32, 32.15, 31.36, 29.94, 26.89, 26.83, 26.52, 26.46, 26.44, 25.81, 17.80, 16.18, 16.13

Compounds 3 [20]

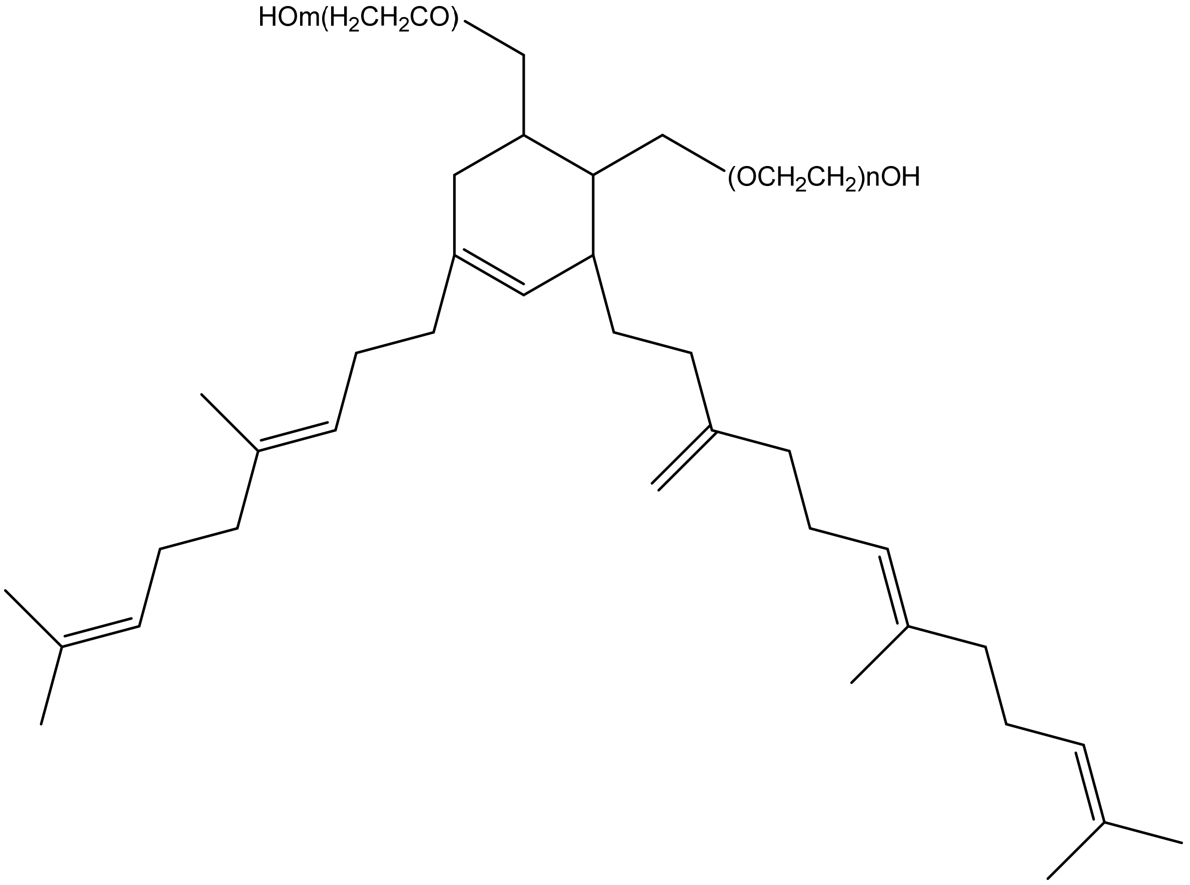

Cis and trans, racemic

Compounds 3, sample 1, average value of $m + n$ was approximately 9.5.

Diols B (10 g, 20 mmol) were added to 70 mL of toluene. Potassium hydroxide (2.3 g, 20 mmol) was added followed by oxirane (85 mL x 3 M in THF, 12 equiv). The mixture was transferred to an autoclave, which was evacuated with nitrogen once. The autoclave was heated to 120ºC and stirred overnight. The heating was stopped, and the vessel was cooled to room temperature. Most of the solvent was removed by rotary evaporation at reduced pressure. The mixture was dissolved in THF, and then 1.5 equivalents of ammonium chloride were added relative to potassium hydroxide. This mixture was heated at 65ºC for 2 h. Then the desired product was purified by silica-gel chromatography (100 g, 200-300 mesh) using methylene chloride: methanol was 20:1 as eluent. 11 g of molecules 3 were obtained as a light brown oil (yield was 58.3%).

1H NMR: 5.25 (broad singlet, 1H for each mixture of diastereoisomers), 5.04-5.16 (multiplet, 4H for each mixture of diastereoisomers), 4.71 (broad singlet, 2H for each mixture of diastereoisomers), 3.30-4.30 (multiplet, 42H for each mixture of diastereoisomers), 1.90-2.20 (multiplet, 23H for each mixture of diastereoisomers), 1.67 (broad singlet, 6H for each mixture of diastereoisomers), 1.60 (broad singlet, 12H for each mixture of diastereoisomers), 1.45 (multiplet, 1H for each mixture of diastereoisomers), 1.27 (multiplet, 1H for each mixture of diastereoisomers).

Alcohols D [23]

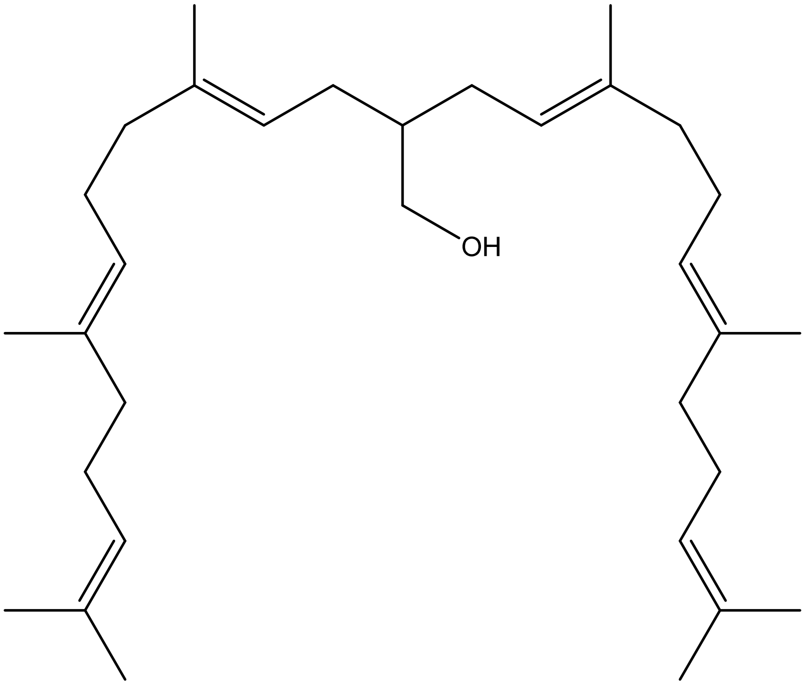

A solution of monoesters A (26 g, 0.054 mol) was added to a solution of 1M lithium aluminum hydride in THF (43 mL, 0.043 mol) at 0-5°C under a nitrogen atmosphere. The reaction mixture was maintained for 3-4 h at 0-5°C. The reaction mixture was carefully quenched with a mixture of THF and water (5 vol) at 0-5°C. pH of the reaction mass was adjusted to 1 with 1M aqueous hydrochloric acid (3 vol) at 5-10°C. The organic layer was separated, and the aqueous layer was extracted with ethyl acetate (2 x 5 vol). The combined organic layer was dried over sodium sulfate and evaporated under vacuum at 40°C to get crude alcohols D. The crude was purified by column chromatography on silica gel (100-200 mesh) and eluted with 8-10% ethyl acetate in hexanes. All the fractions containing pure product were collected and dried under vacuum at 40°C to afford alcohols D (17.2 g; 70.2 % yield; 99.17% HPLC purity, AUC by HPLC: 82.0% and 17.17% for two peaks) as a pale yellow liquid.

1H NMR: 5.17 (broad triplet, 2H, J = 6.8 Hz), 5.07-5.15 (m, 4H), 3.53 (broad singlet, 2H), 2.14–1.95 (m, 21H), 1.68 (singlet, 6H), 1.65 (singlet, 6H), 1.59 (broad singlet, 12H).

13C NMR: 136.52, 135.09, 131.28, 124.38, 124.10, 122.64, 66.10, 42.16, 39.87, 39.73, 29.68, 26.74, 26.59, 25.71, 17.68, 16.13, 16.02.

M/Z: 455 ([M+H]+, positive ion)

Ether 2 [24]

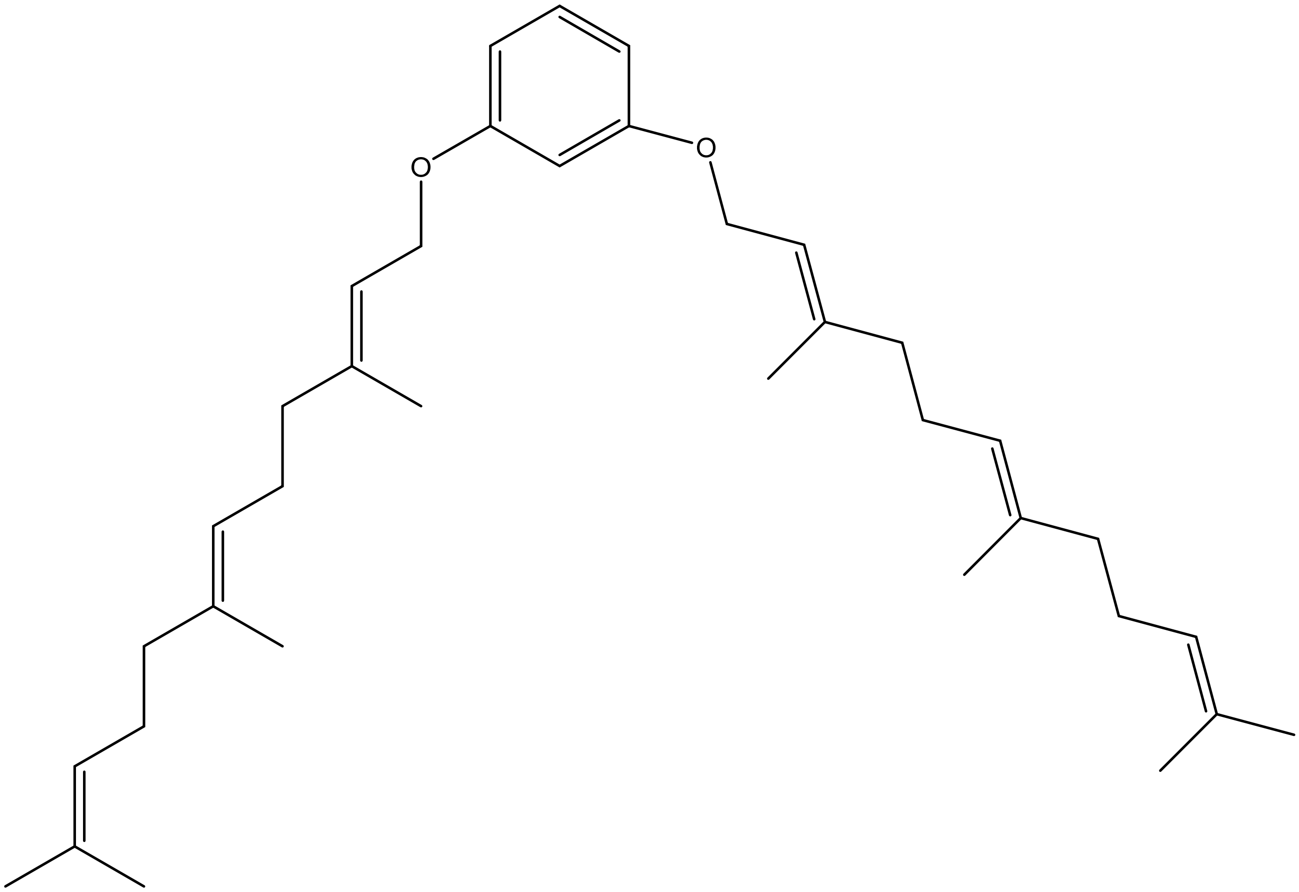

N,N–Dimethylformamide (37.5 mL, 25 vol), resorcinol (1.50 g, 14 mmol), and potassium carbonate (18.82 g, 136 mmol, 10 equiv) were added into a 250-mL three-neck round-bottom flask at room temperature under a nitrogen atmosphere. The resulting reaction mixture was heated to 75–80°C and stirred for 2 h. The reaction mass was cooled to room temperature, farnesyl chloride (13.3 g, 54 mmol) was slowly added, and then the mixture was heated to 55–60°C. The resulting mixture was stirred at 55–60°C overnight. The reaction conversion was monitored by TLC (5% ethyl acetate in hexane). Upon completion of the reaction, the mixture was cooled to room temperature and diluted with water (200 mL), and the compound was extracted with methyl tert-butyl ether (2 x 100 mL). The organic layer was dried over anhydrous sodium sulfate followed by concentration below 40°C under reduced pressure to obtain crude Target 6. The crude Target 6 was purified by flash chromatography using ethyl acetate and hexanes. The product was eluted at 3–6% ethyl acetate in hexanes. All the pure fractions were combined and concentrated to obtain pure ether 2 (3 g; 43.4% yield; 95.6% purity, AUC by HPLC) as a pale yellow liquid.

1H NMR**:** 7.14 (m, 1H), 6.50 (m, 3H), 5.49 (t, 2H, J = 6.4 Hz), 5.15-5.05 (m, 4H), 4.49 (d, 4H, J = 6.4 Hz), 2.18–2.03 (m, 12H), 1.99–1.95 (m, 4H), 1.75 (s, 6H), 1.67 (s, 6H), 1.60 (s, 12H).

13C NMR**:** 160.13, 141.15, 135.43, 131.31, 129.73, 124.36, 123.74, 119.52, 106.92, 101.79, 64.85, 39.71, 39.59, 26.74, 26.27, 25.71, 17.70, 16.67, 16.03.

M/Z: 520 ([M+H]+, positive ion)

Ether 3 [25]

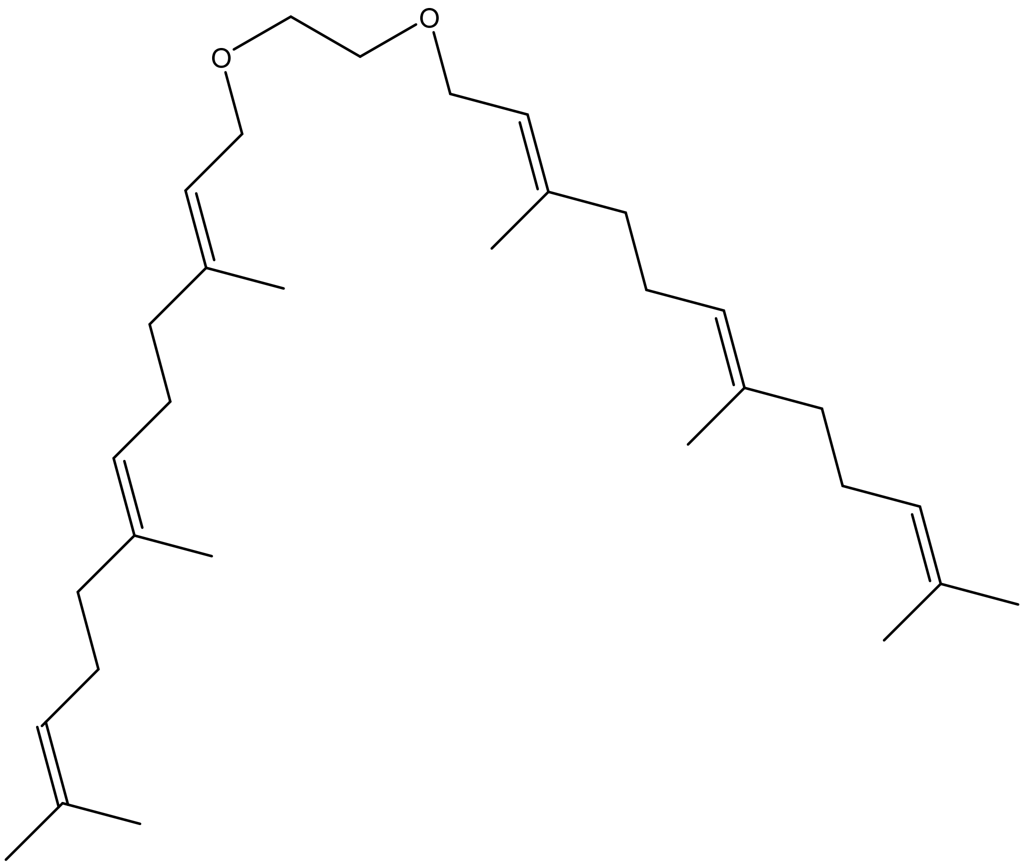

Ethylene glycol (4 g, 64.5 mmol) was added to a stirred solution of sodium hydroxide (15.47 g, 390 mmol) and water (32 mL, 8 vol) in a 500-mL three-neck round-bottom flask at room temperature. The resulting mixture was heated to 90–95°C and stirred for 2 h. The reaction mixture was cooled to room temperature, and then farnesyl chloride (77.40 g, 322 mmol) was added slowly into the reaction followed by solid tetrabutylammonium iodide (4.76 g, 12.9 mmol). The mixture was heated at 50–55°C and maintained overnight at 50–55°C. The reaction mixture was cooled to room temperature and then diluted with 40 mL of water and extracted with methyl tert-butyl ether (2 x 100 mL). The combined organic layers were washed with 20 mL of saturated brine solution, dried over 4 g of anhydrous sodium sulfate, filtered, and then dried under vacuum below 40°C to obtain crude ether 3. This crude product was purified by silica-gel (100-200 mesh size) column chromatography and eluted with 4-6% ethyl acetate in petroleum ether. All the pure fractions were collected and dried under vacuum to afford 7.5 g of ether 3 (59% yield).

1H NMR: 5.37 (broad triplet, J = 6.8 Hz, 2H), 5.09 (m, 4H), 4.04 (d, J = 6.8 Hz, 4H), 3.62 (s, 4H), 2.09 (m, 16H), 1.67 (s, 6H), 1.66 (s, 6H) 1.60 (s, 12H).

13C NMR: 140.02, 135.25, 131.30, 124.35, 123.90, 120.92, 69.27, 67.71, 39.71, 39.63, 26.73, 26.31, 25.71, 17.70, 16.50, 16.01.

M/Z: 488 $[M+NH4]^{+}$

Ether 4 [26]

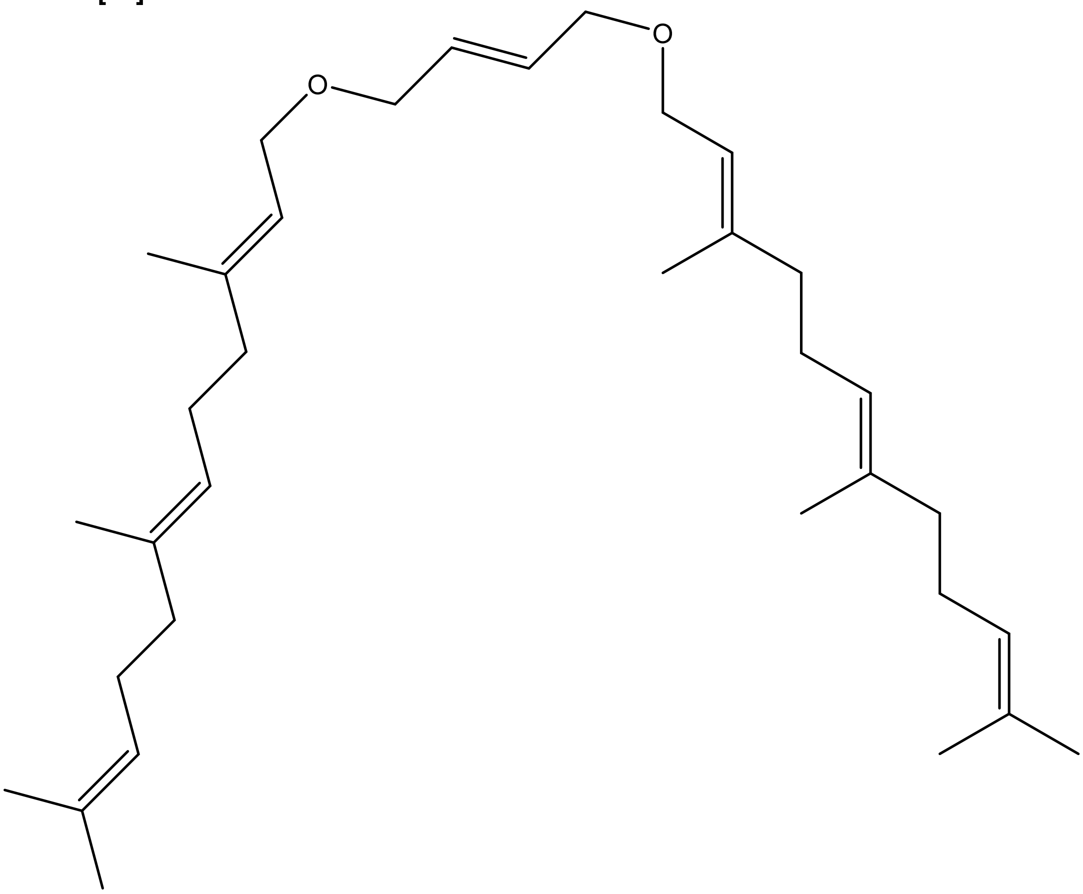

Trans-2-butene-1,4-diol (4 g, 45.4 mmol) was added to a stirred solution of sodium hydroxide (10.9 g, 272 mmol) and water (24 mL, 6 vol) in a 500-mL three-neck round-bottom flask at room temperature and then heated at 90–95°C. The resulting reaction mixture was stirred for 2 h at 90–95°C. The mixture was cooled to room temperature, and farnesyl chloride (54.56 g, 227 mmol) was added slowly into the reaction mass and then charged with tetrabutylammonium iodide (3.36 g, 9.09 mmol). The reaction was heated at 50–55°C overnight. The reaction mass was then cooled to room temperature, and the reaction was diluted with water (40 mL, 10 vol) and extracted with methyl tert-butyl ether (2 x 100 mL). Combined organic layers were washed with 20 mL of saturated brine solution. The organic layer was dried over 4 g of anhydrous sodium sulfate, and then the solvent was removed under vacuum below 40°C. This crude material was purified by column chromatography using silica gel (100-200 mesh), and the compound was eluted with 4–6 % ethyl acetate in petroleum ether. All the pure fractions were collected, and solvents were removed under vacuum to afford pure ether 4 as a pale yellow liquid.

1H NMR: 5.85 (m, 2H), 5.37 (broad triplet, J = 6.8 Hz, 2H), 5.12 (m, 4H), 3.99 (m, 8H), 2.18-1.95 (m, 16H), 1.70 (s, 6H), 1.68 (s, 6H) 1.62 (s, 12H).

13C NMR: 140.29, 135.27, 131.30, 124.35, 123.88, 120.75, 69.98, 66.62, 39.71, 39.61, 26.73, 26.32, 25.71, 17.70, 16.53, 16.01.

M/Z: 514 $[M+NH4]^+$

Ether 5 [27]

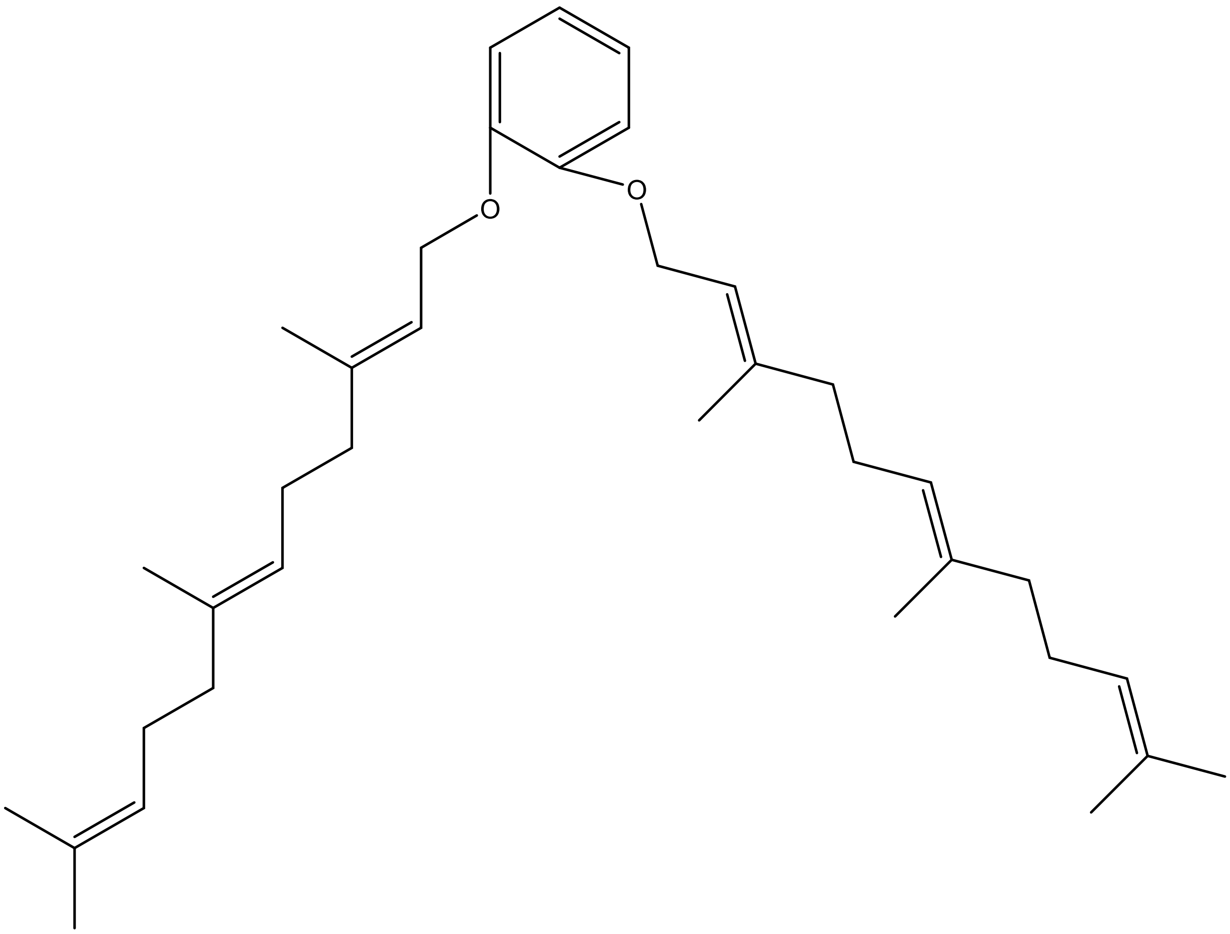

Potassium carbonate (18.82g, 136 mmol, 6 equiv) was added to a solution of catechol (2.50 g, 23 mmol, 1 equiv) in N,N–dimethylformamide (50 mL, 20 vol.) under a nitrogen atmosphere at 25-30°C. The reaction mass was heated to 55-60°C for 1 h. The reaction mass was then cooled to 25°C. Farnesyl chloride (21.8 g, 90.8 mmol, 4 equiv) was slowly added to the above stirred mixture over a period of 15 min. The mixture was heated to 55–60°C and stirred for 5-6 h. Reaction conversion was monitored by TLC (mobile phase: 5% ethyl acetate in hexanes). The reaction mass was cooled and diluted with water (250 mL) at 25-30°C. The product was extracted with methyl tert-butyl ether (2 × 100 mL) at 25°C. The combined organic layer was washed with water (5 vol) followed by brine (5 vol). The organic layer was dried over anhydrous sodium sulfate and evaporated under reduced pressure below 40°C to obtain crude ether 5. The crude was purified by column chromatography on silica gel (100–200 mesh) and eluted using 2-5% ethyl acetate in hexanes. All the pure fractions were combined and evaporated to obtain pure ether 5 (9 g; 80.3% yield; 96.3% purity, AUC by HPLC) as a yellow liquid.

1H NMR: 6.87 (m, 4H), 5.52 (t, 2H, J = 6.0 Hz), 5.08 (m, 4H), 4.60 (d, 4H, J = 6.4 Hz), 2.15–2.09 (m, 4H), 2.07–2.04 (m, 8H), 1.98–1.94 (m, 4H), 1.71 (s, 6H), 1.67 (s, 6H), 1.59 (s, 12H).

13C NMR: 148.95, 140.15, 135.32, 131.27, 124.39, 123.84, 121.01, 120.27, 114.23, 66.09, 39.73, 33.59, 26.76, 26.30, 25.74, 17.72, 16.72, 16.04.

M/Z: 519.69 $[M+H]^+$ and 541.66 $[M+Na]^+$

Ether 6 [28]

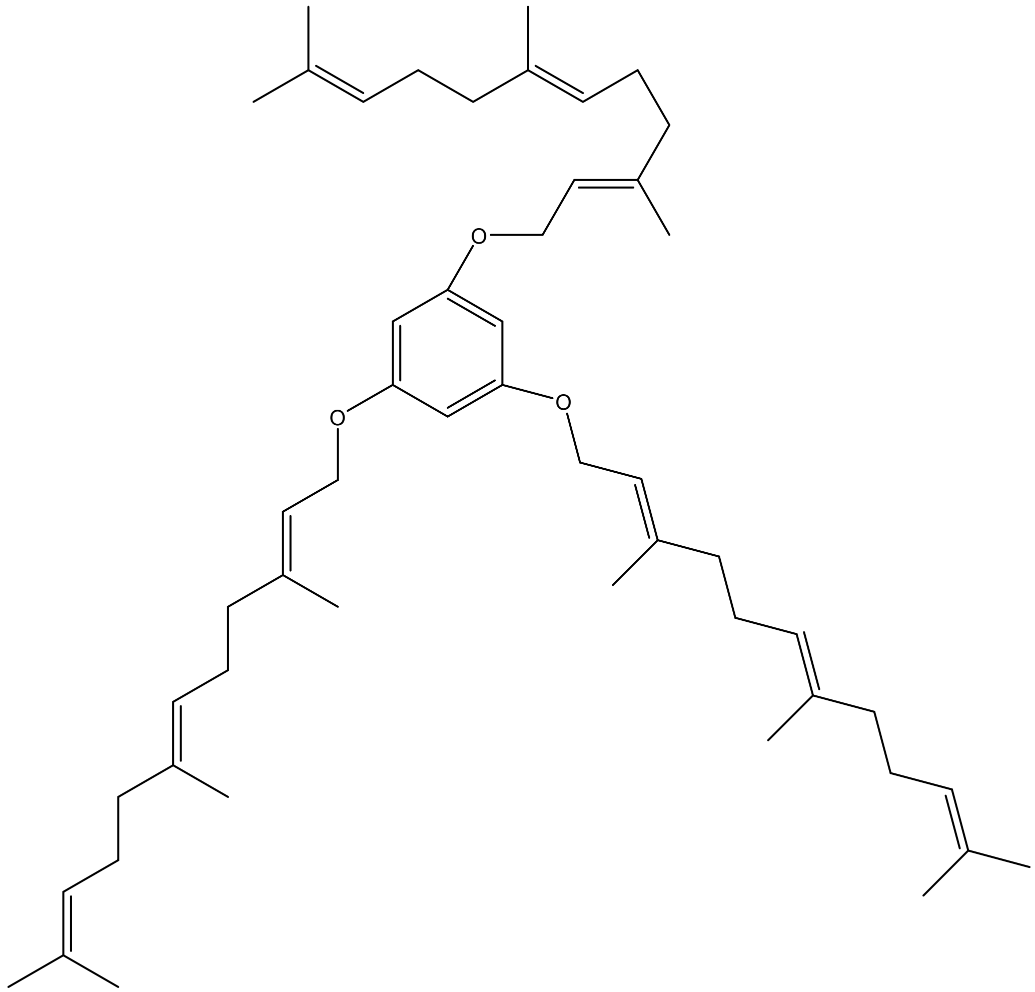

Potassium carbonate (32.8 g, 238 mmol, 10 equiv) was added to a solution of phloroglucinol (3 g, 23.8 mmol, 1 equiv) in N,N–dimethylformamide (60 mL, 20 vol.) under a nitrogen atmosphere at 25-30°C. The reaction mass was heated to 55-60°C for 1 h. The reaction mass was then cooled to 25°C. Farnesyl chloride (45.8 g, 190 mmol, 8 equiv) was slowly added to the above stirred mixture over a period of 15-20 min. The reaction mass was heated to 55–60°C and stirred for 16-20 h. The reaction mass was cooled to 25-30°C and diluted with 300 mL of water at 25-30°C. The product was extracted with methyl tert-butyl ether (2 × 150 mL) at 25°C. The combined organic layers were washed with 50 mL of water followed by 50 mL of brine. The organic layer was dried over anhydrous sodium sulfate, and the solvent was removed under reduced pressure below 40°C to obtain crude ether 6. The crude ether 6 was purified by column chromatography on basic alumina and eluted using 0.5-1% ethyl acetate in hexanes. All the pure fractions were combined and evaporated to obtain ether 6 (8.50 g; 48.3% yield; 79.8% purity, AUC by HPLC) as a pale yellow liquid. Additional purification was performed by two methods:

- Purification method 1: The ether 6 (~8 g, different batches) was purified by reverse-phase CombiFlash, eluted with 95-100% acetonitrile, and isolated as 1.6 g of pure material with 94.91% HPLC purity.
- Purification method 2: The ether 6 (~18 g, different batches) was purified by using a supercritical fluid chromatography (SFC) purification method and isolated as 3.6 g of pure material with 94.91% HPLC purity.

All pure fractions were dissolved in dichloromethane, mixed, and evaporated under vacuum to obtain 5.2 g of ether 6 with 96.4% HPLC purity (29% yield).

1H NMR: 6.13 (s, 3H), 5.50 (t, 3H, J = 6.0 Hz), 5.11 (m, 6H), 4.48 (d, 6H, J = 6.8 Hz), 2.16–1.97 (m, 24H), 1.73 (s, 9H), 1.69 (s, 9H), 1.61 (s, 18H).

13C NMR: 158.67, 139.17, 133.39, 122.37, 121.74, 117.42, 92.12, 62.83, 37.72, 37.59, 24.73, 24.26, 23.72, 15.71, 14.66, 14.03.

M/Z: 740.3 $[M+H]^+$

SUPPLEMENTARY FIGURES

a

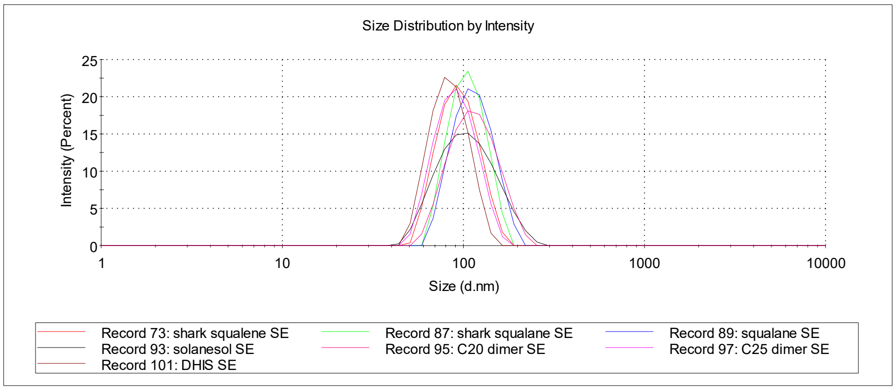

b

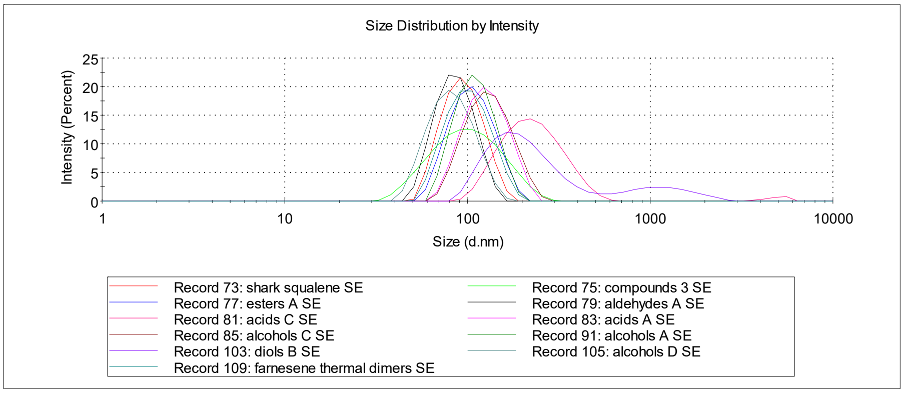

c

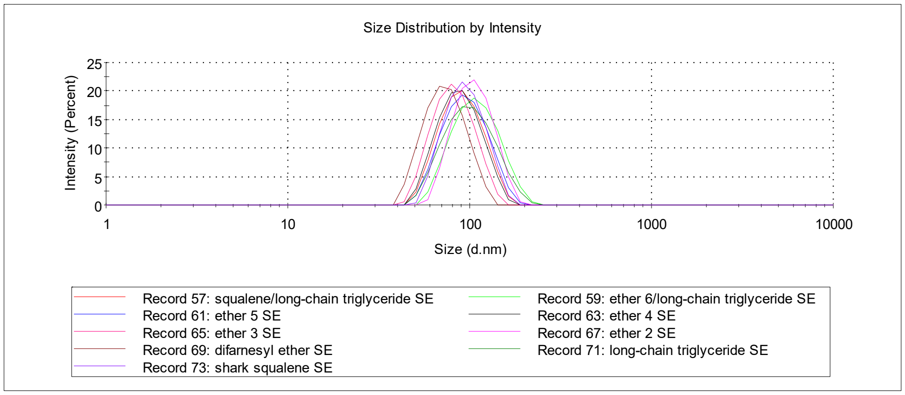

d

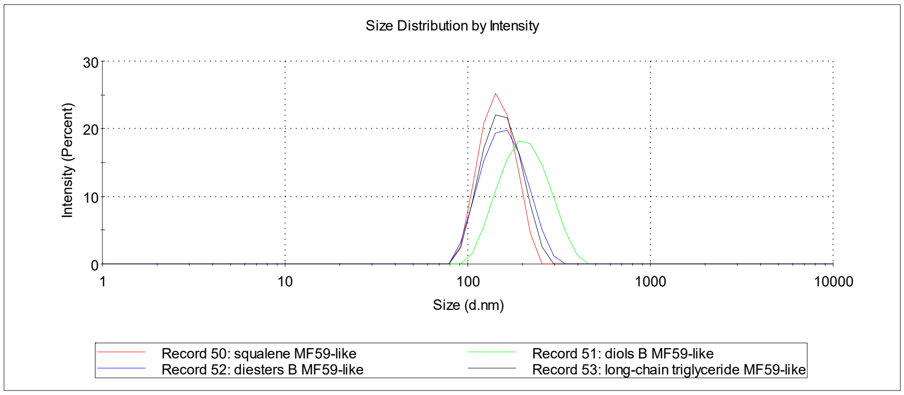

Supplementary Figure 1. Dynamic light scattering intensity-based size distributions. (**a**) Linear terpenoid SE series, (**b**) cyclohexene terpenoid SE series, (**c**) ether terpenoid SE series, (**d**) MF59-like formulations of selected cyclohexene-containing terpenoids.

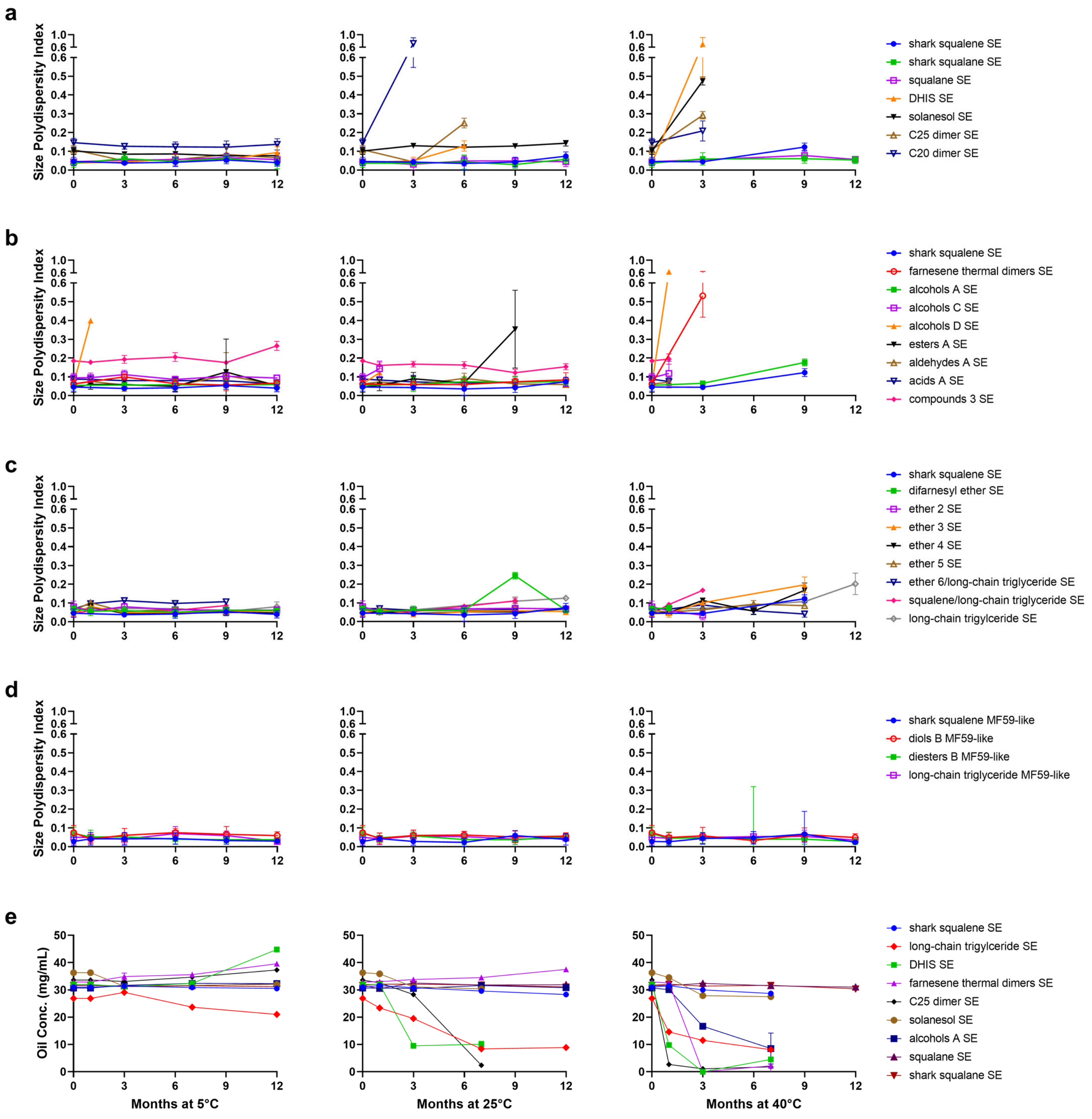

Supplementary Figure 2. Size polydispersity index and chemical stability of oil-in-water emulsions. Emulsions were stored at 5°C, 25°C, or 40°C for up to 12 months and monitored by dynamic light scattering and HPLC with charged aerosol detection to assess (**a-d**) size polydispersity index and (**e**) terpenoid concentration in selected emulsions. Data are represented as means with error bars representing standard deviation of the measurement.

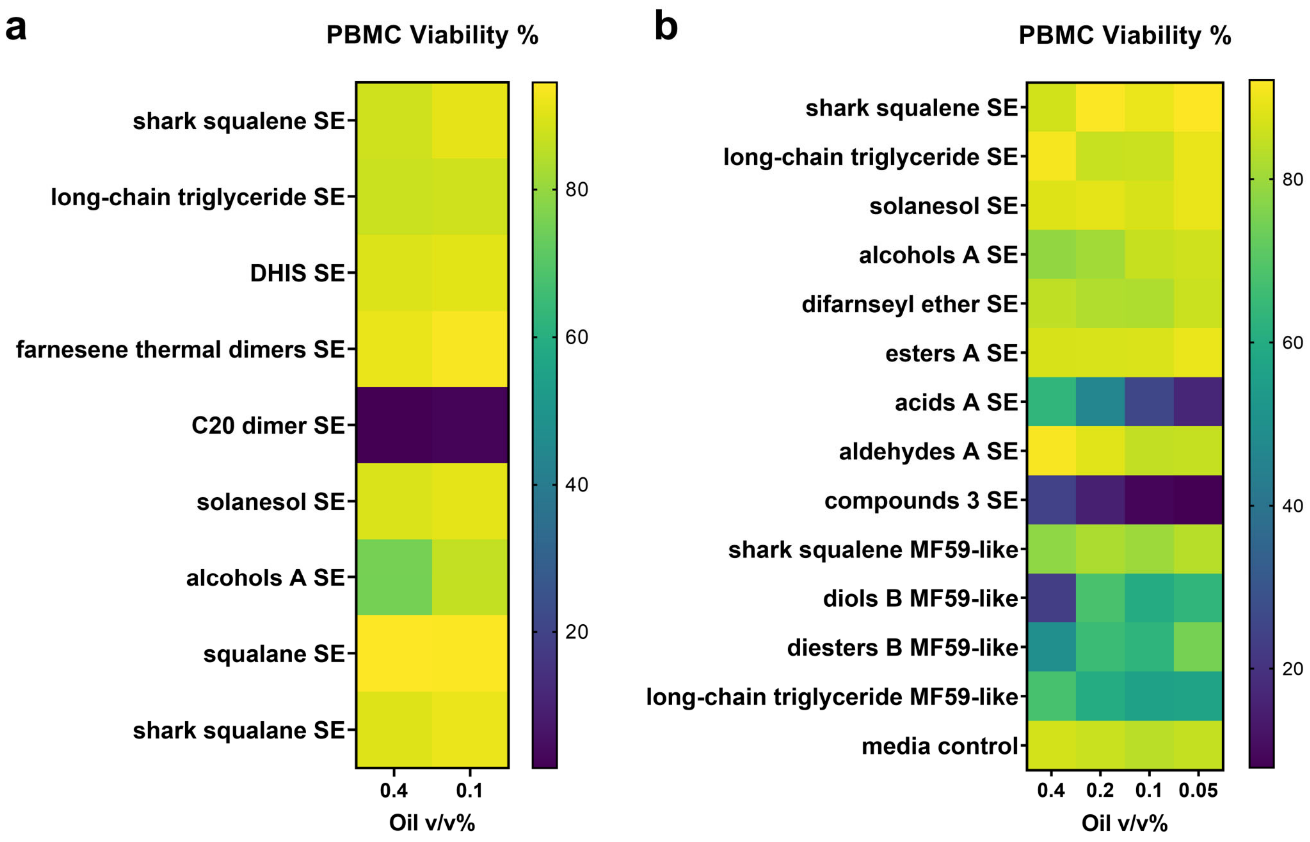

Supplementary Figure 3. Viability of peripheral blood mononuclear cells (PBMCs) following incubation with selected oil-in-water emulsions. (**a**) PBMC viability with a first experimental set of oil-in-water emulsions. The emulsions in this case did not include α-tocopherol, but some included butylated hydroxytoluene (BHT) or nitrogen overlay to reduce potential oxidative degradation. PBMCs from 4 donors (2 male and 2 female) were employed. (**b**) PBMC viability with a second experimental set of oil-in-water emulsions. PBMCs from 3 donors (2 male and 1 female) were employed.

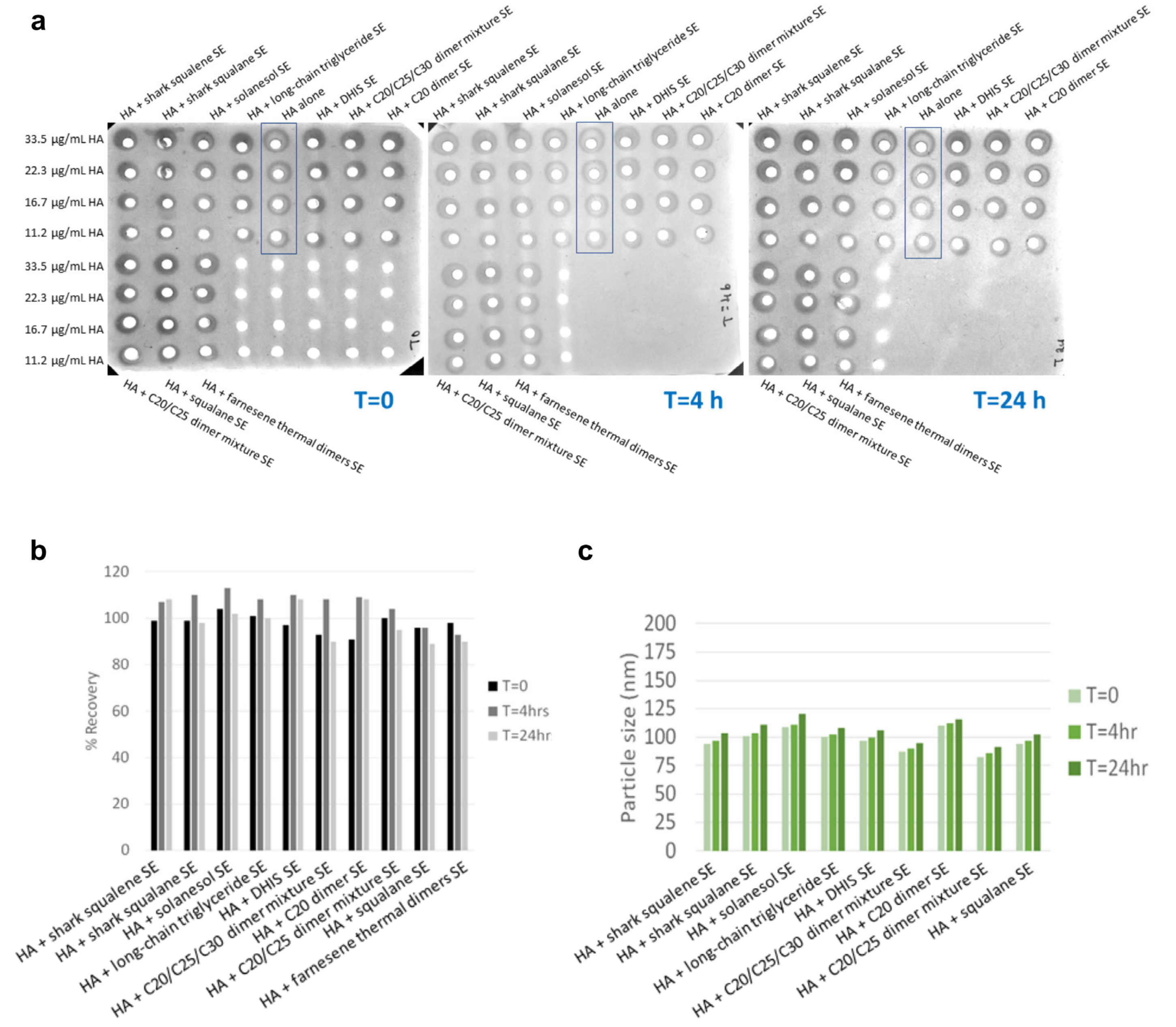

Supplementary Figure 4. Antigen-adjuvant short-term compatibility following mixing. Split, inactivated H5N1 antigen (HA) was mixed with selected oil-in-water emulsions at 2% v/v oil. At 0, 4, and 24 h after mixing, antigen conformation was monitored by (**a**) SRID gels, with calculated diameters compared to a standard to determine (**b**) % recovery of antigen. (**c**) Emulsion droplet diameter was monitored by dynamic light scattering at 0, 4, and 24 h after mixing with H5N1 antigen.

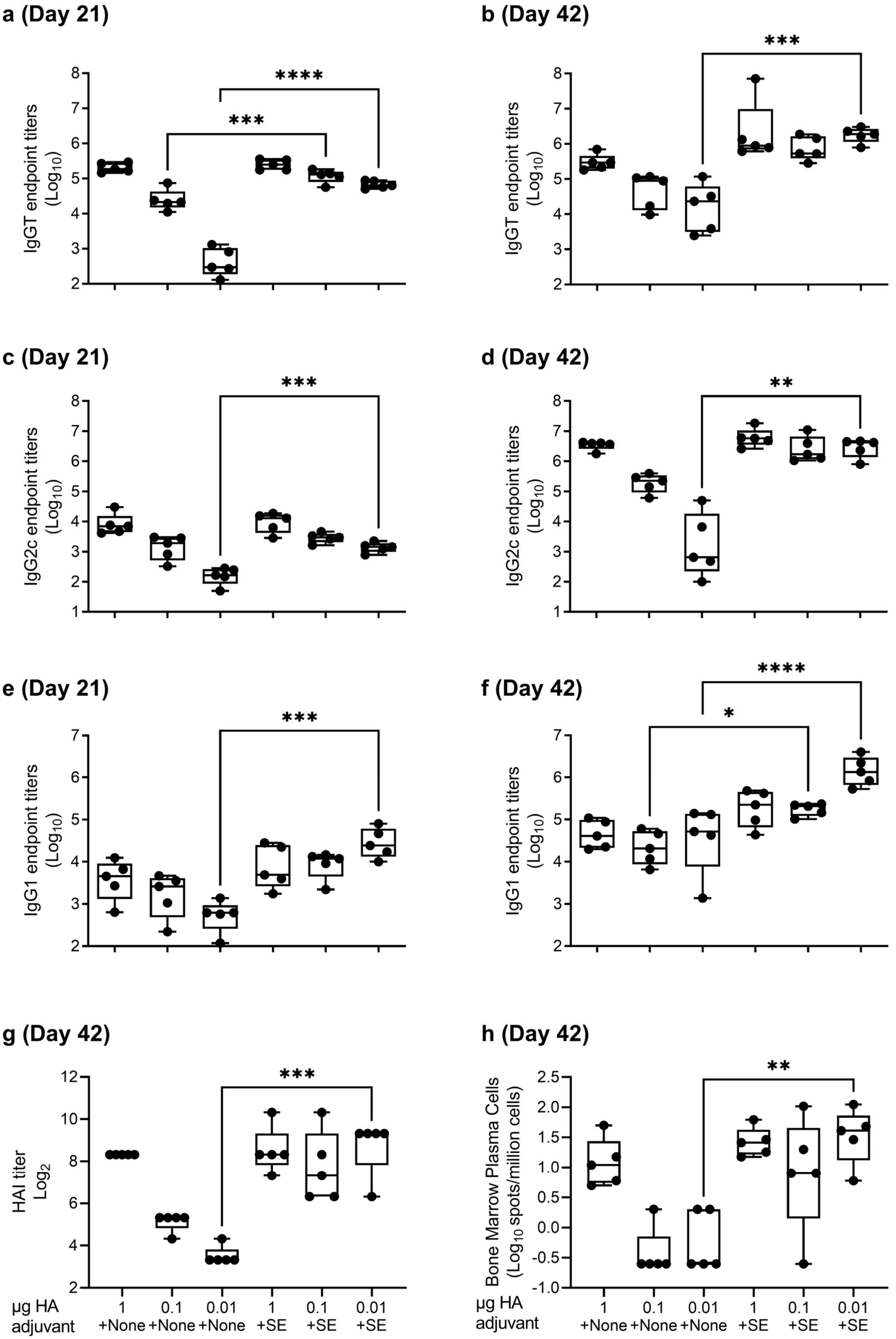

a (Day 21)
b (Day 42)
c (Day 21)
d (Day 42)
e (Day 21)
f (Day 42)
g (Day 42)
h (Day 42)
IgGT endpoint titers (Log10)
IgG2c endpoint titers (Log10)
IgG1 endpoint titers (Log10)
HAI titer Log2
Bone Marrow Plasma Cells (Log10 spots/million cells)
µg HA
adjuvant
1 +None
0.1 +None
0.01 +None
1 +SE
0.1 +SE
0.01 +SE

Supplementary Figure 5 (previous page). Effect of antigen dose on antigen-specific immunogenicity in mice immunized intramuscularly with split, inactivated H5N1 influenza antigen alone or mixed with shark squalene emulsion. Related to Figure 3. Vaccine antigen was mixed with shark squalene emulsion (2% v/v oil) immediately prior to intramuscular immunization of female C57BL/6 mice (*n* = 5). The emulsion composition used in this experiment was similar to that described in the main manuscript except that the stock concentration was prepared at 10% v/v oil, and it did not contain α-tocopherol. Data are represented as box-whisker plots with bars representing median values, boxes representing 1st-3rd quartiles, and whiskers representing the maximum and minimum values. Statistical evaluation of each experimental group compared to antigen alone at the same dose was performed by one-way ANOVA with Sidak's correction for multiple comparisons or the Kruskal-Wallis test with Dunn's correction for multiple comparisons as indicated; *p-value < 0.05, **p-value < 0.01, ***p-value < 0.001, ****p-value < 0.0001. (**a**) Antigen-specific total IgG (IgGT) serum titers measured by ELISA 21 days after prime immunization (one-way ANOVA). (**b**) Antigen-specific IgGT serum titers measured by ELISA 42 days after prime immunization (Kruskal-Wallis test). (**c**) Antigen-specific IgG2c serum titers measured by ELISA 21 days after prime immunization (one-way ANOVA). (**d**) Antigen-specific IgG2c serum titers measured by ELISA 42 days after prime immunization (Kruskal-Wallis test). (**e**) Antigen-specific IgG1 serum titers measured by ELISA 21 days after prime immunization (Kruskal-Wallis test). (**f**) Antigen-specific IgG1 serum titers measured by ELISA 42 days after prime immunization (one-way ANOVA). (**g**) HAI titers measured 42 days after prime immunization (Kruskal-Wallis test). (**h**) Long-lived antibody-secreting cells in the bone marrow measured by ELISpot assay 42 days after prime immunization (Kruskal-Wallis test).

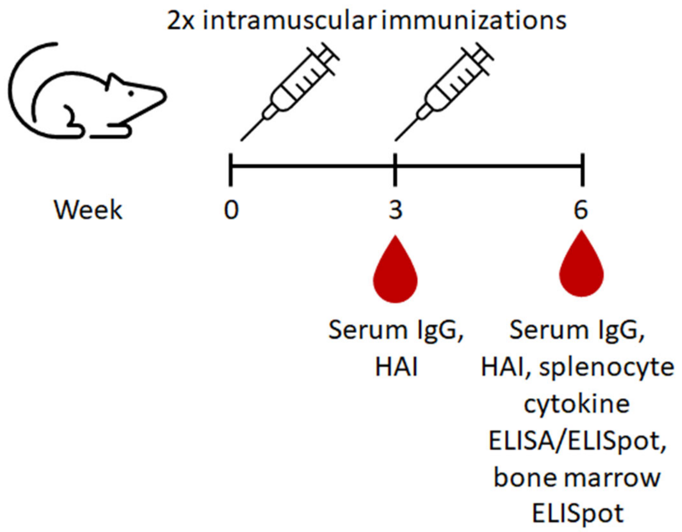

Supplementary Figure 6. Experimental design of mouse immunogenicity studies. Related to Figure 3 in the manuscript.

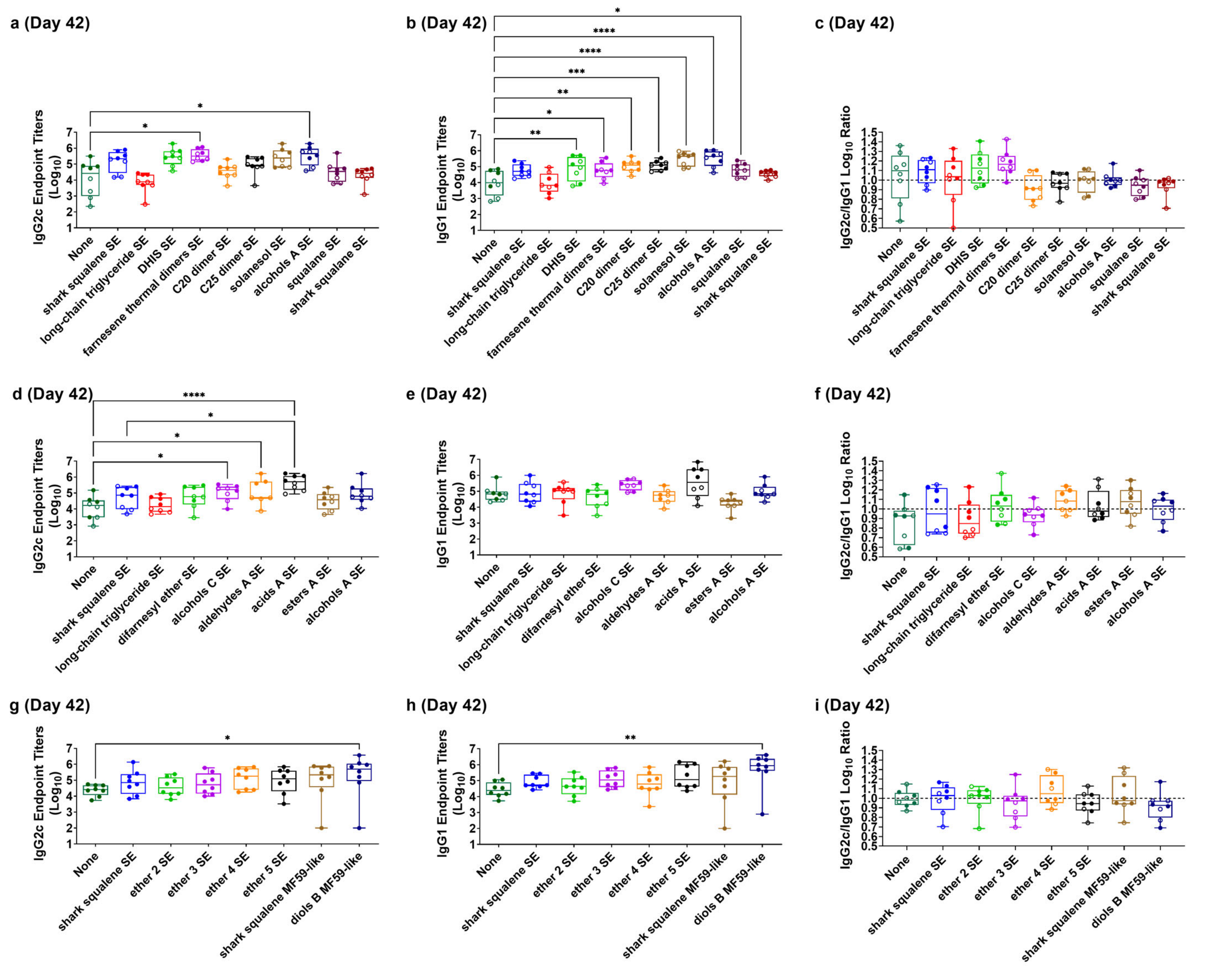

Supplementary Figure 7. Antigen-specific serum IgG2c and IgG1 titers from immunized mice. Vaccine antigen was mixed with the indicated terpenoid oil emulsion immediately prior to intramuscular immunization of C57BL/6 mice (male [open circles] and female [closed circles]) such that each animal received 10 ng of antigen in 2% v/v terpenoid emulsion. Negative control groups received antigen alone or antigen mixed with long-chain triglyceride emulsion. Three separate experiments each with different terpenoids as indicated are represented (**a**-**c**, **d**-**f**, and **g**-**i**). Data are represented as box-whisker plots with bars representing median values, boxes representing 1st-3rd quartiles, and whiskers representing the maximum and minimum values. Statistical evaluation of each terpenoid emulsion group compared to antigen alone (negative control) and shark squalene emulsion (positive control) was performed by one-way ANOVA with Sidak's correction for multiple comparisons or the Kruskal-Wallis test with Dunn's correction for multiple comparisons as indicated; *p-value < 0.05, **p-value < 0.01, ***p-value < 0.001, ****p-value < 0.0001. (**a**,**d**,**g**) Antigen-specific IgG2c serum titers measured by ELISA 42 days after prime immunization (n = 8 mice, Kruskal-Wallis test for panels **a** and **g**; n = 7-8 mice, one-way ANOVA for panel **d**). (**b**,**e**,**h**) Antigen-specific IgG1 serum titers measured by ELISA 42 days after prime immunization (n = 8 mice, ANOVA for panel **b**; n = 7-8 mice, Kruskal-Wallis test for panels **e** and **h**). (**c**,**f**,**i**) Serum IgG2c/IgG1 ratio 42 days after prime immunization (n = 8 mice, Kruskal-Wallis test for panels **c** and **i**; n = 7-8 mice, one-way ANOVA for panel **f**). Related to Figure 3 in the manuscript.

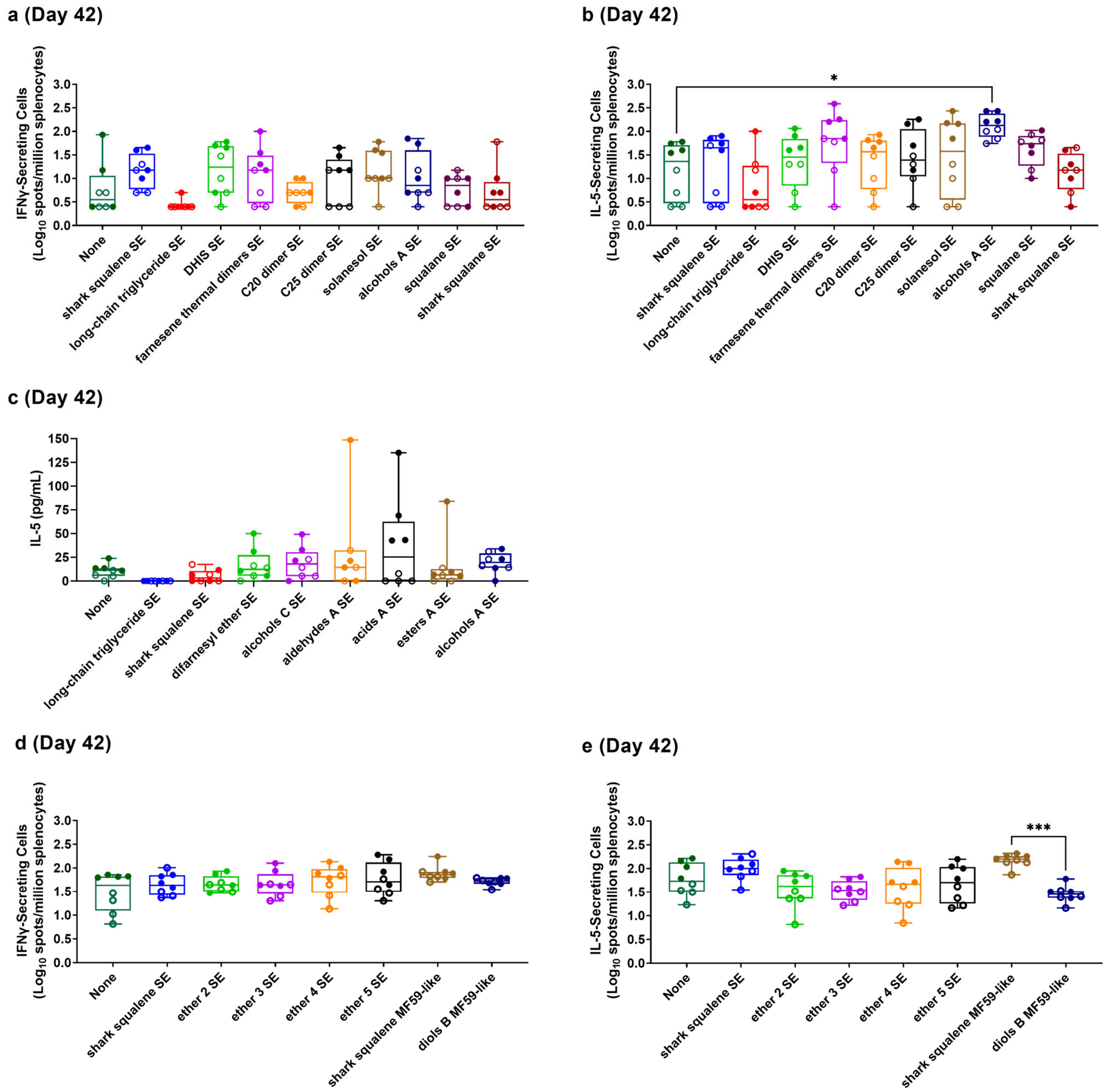

Supplementary Figure 8. Cytokines produced by antigen-stimulated splenocytes from immunized C57Bl/6 mice. Data are represented as box-whisker plots with bars representing median values, boxes representing 1st-3rd quartiles, and whiskers representing the maximum and minimum values. Male animals are represented by open circles and female animals by closed circles. Statistical evaluation of each terpenoid emulsion group compared to antigen alone (negative control) and shark squalene emulsion (positive control) was performed by one-way ANOVA with Sidak's correction for multiple comparisons or the Kruskal-Wallis test with Dunn's correction for multiple comparisons as indicated; **p*-value < 0.05, ****p*-value < 0.001. (**a**) Enumeration of IFNγ-producing cells by ELISpot assay following stimulation of splenocytes with H5N1 antigen where spleens were harvested 3 weeks after the second immunization with the first set of experimental oils (*n* = 8 mice, Kruskal-Wallis test). (**b**) Enumeration of IL-5-producing cells by ELISpot assay following stimulation of splenocytes with H5N1 antigen where spleens were harvested 3 weeks after the second immunization with the first set of experimental oils (*n* = 8 mice, one-way ANOVA). (**c**) Secreted IL-5 concentration produced by antigen-stimulated splenocytes as measured by ELISA where spleens were harvested 3 weeks after the second immunization with the second set of experimental oils (*n* = 7-8 mice, Kruskal-Wallis test). Secreted IFNγ quantitation was also attempted by ELISA but only 3 mice showed detectable levels. (**d**) Enumeration of IFNγ-producing cells by ELISpot assay following stimulation of splenocytes with H5N1 antigen where spleens were harvested 3 weeks after the second immunization with the third set of experimental oils (*n* = 8 mice, Kruskal-Wallis test). (**e**) Enumeration of IL-5-producing cells by ELISpot assay following stimulation of splenocytes with H5N1 antigen where spleens were harvested 3 weeks after the second immunization with the third set of experimental oils (*n* = 8 mice, Kruskal-Wallis test). Related to Figure 3 in the manuscript.

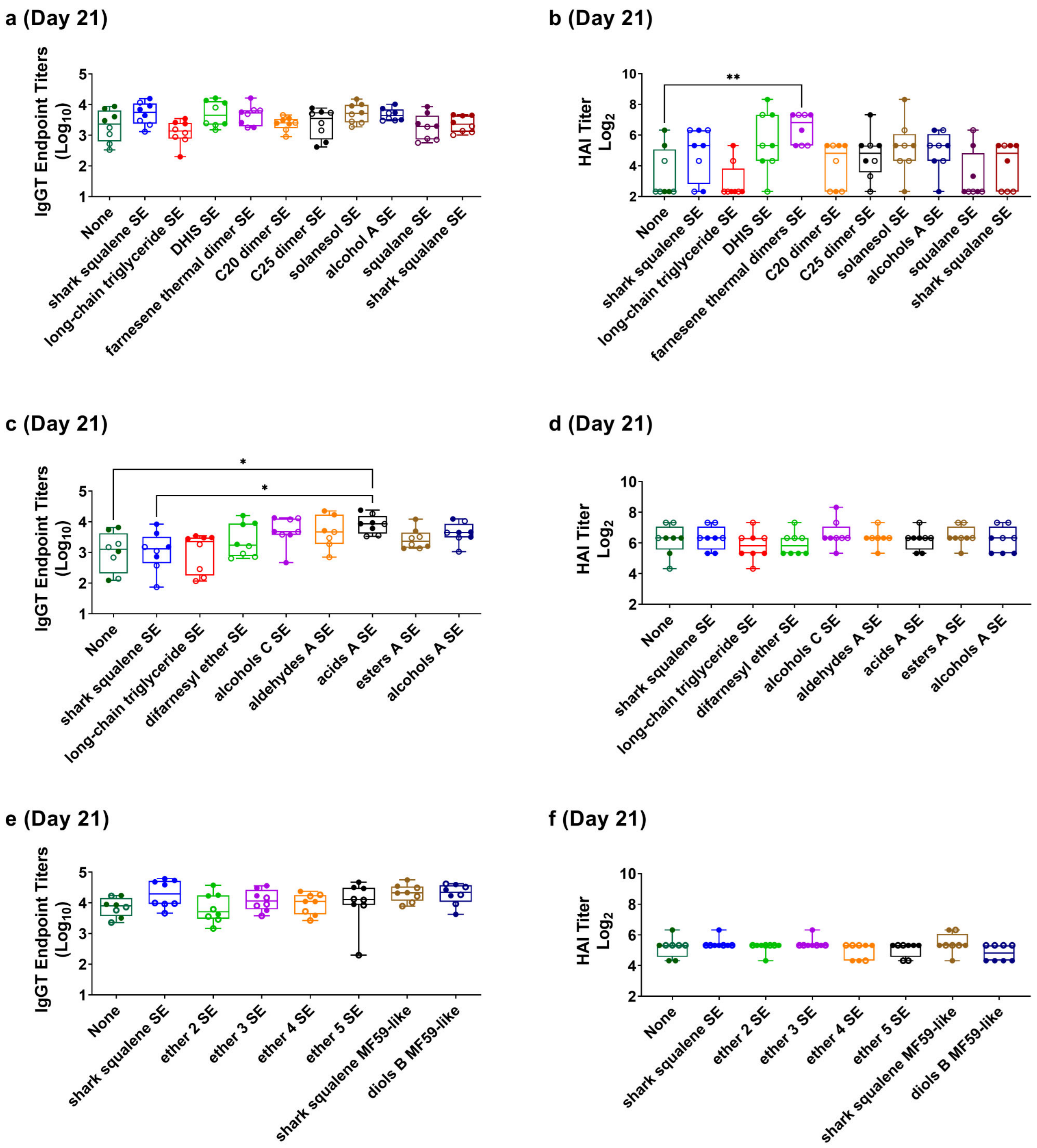

Supplementary Figure 9. Immune responses measured 3 weeks after a single immunization. Vaccine antigen was mixed with the indicated terpenoid oil emulsion immediately prior to intramuscular immunization of C57BL/6 mice (male [open circles] and female [closed circles]) such that each animal received 10 ng of antigen in 2% v/v terpenoid emulsion. Negative control groups received antigen alone or antigen mixed with long-chain triglyceride emulsion. Three separate experiments each with different terpenoids as indicated are represented (**a**-**b**, **c**-**d**, and **e**-**f**). Data are represented as box-whisker plots with bars representing median values, boxes representing 1st-3rd quartiles, and whiskers representing the maximum and minimum values. Statistical evaluation of each terpenoid emulsion group compared to antigen alone (negative control) and shark squalene emulsion (positive control) was performed by one-way ANOVA with Sidak's correction for multiple comparisons or the Kruskal-Wallis test with Dunn's correction for multiple comparisons as indicated; *p-value < 0.05, **p-value < 0.01. (**a**,**c**,**e**) Antigen-specific total IgG (IgGT) serum titers measured by ELISA 21 days after prime immunization (n = 8 mice, one-way ANOVA for panel **a**; n =7-8 mice, Kruskal-Wallis test for panels **c** and **e**). (**b**,**d**,**f**) HAI titers measured 21 days after prime immunization (n = 7-8 mice, Kruskal-Wallis test). Related to Figure 3 in the manuscript.

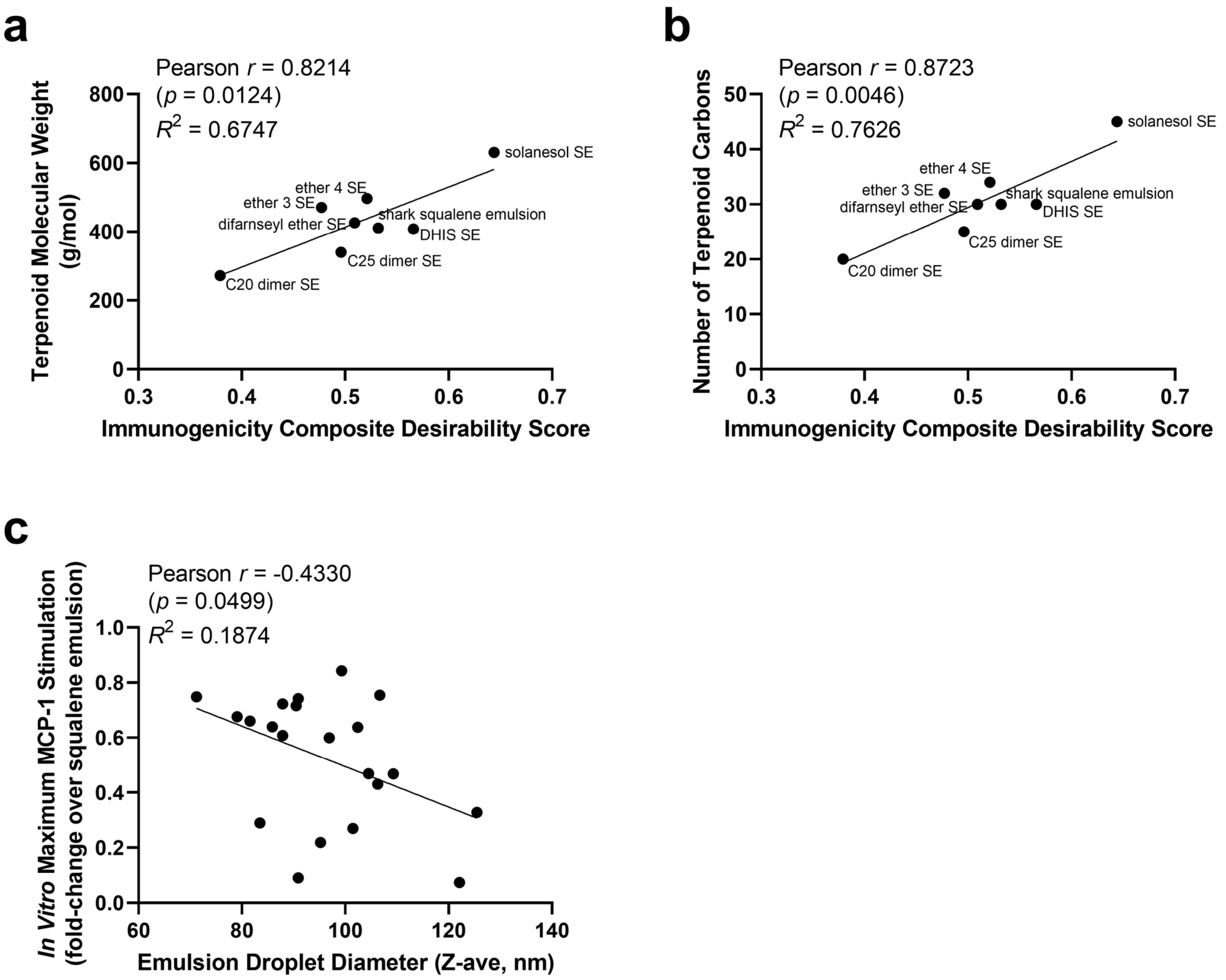

Supplementary Figure 10. Supplementary correlation plots. (**a**-**b**) Immunogenicity composite desirability score of linear unsaturated terpenoids correlates with terpenoid molecular weight (**a**) and number of carbons (**b**). (**c**) *In vitro* human whole blood MCP-1 stimulation activity of SE formulations correlates with emulsion droplet diameter. For all correlation plots, a single mean value was computed for structures that were included in more than one experiment (antigen alone, shark squalene SE/MF59-like, shark squalane/squalane SE, long-chain triglyceride SE, alcohols A SE, and solanesol SE) such that each terpenoid structure is represented only once.

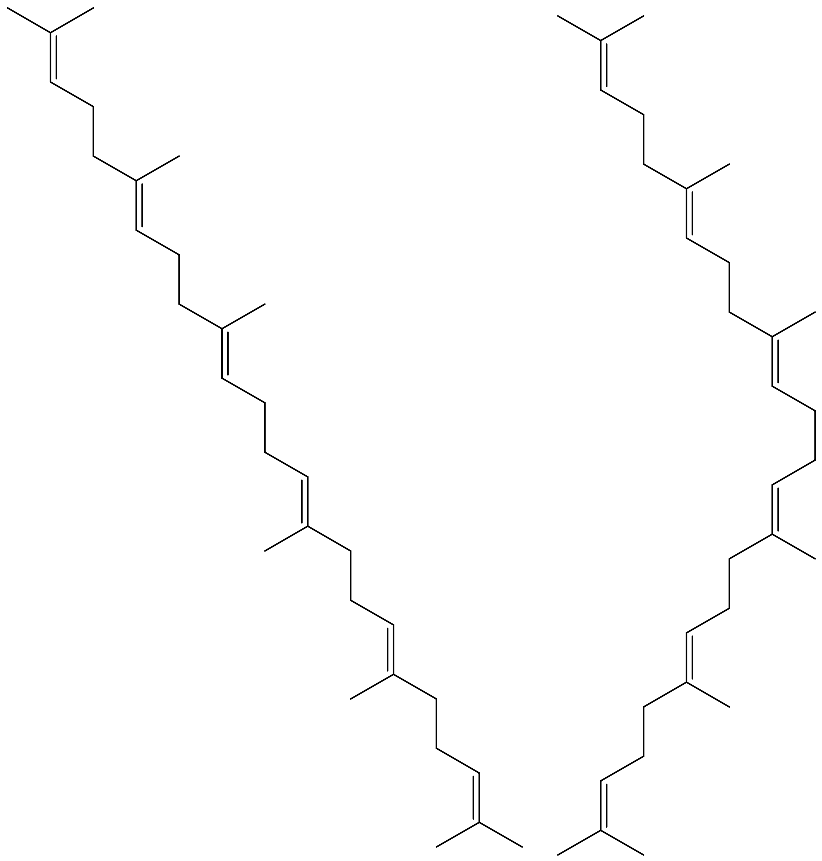

Supplementary Figure 11. Squalene conformations. (Left) Extended conformation of squalene. (Right) Folded squalene conformation at the C11-C12 position.

SUPPLEMENTARY TABLES

Supplementary Table 1. Compound IUPAC names, related to Table 1

Compound Name	Compound Number	IUPAC Name
squalene	1	(6*E*,10*E*,14*E*,18*E*)-2,6,10,15,19,23-hexamethyltetracosa-2,6,10,14,18,22-hexaene
DHIS	2	(6*E*,11*E*,18*E*)-2,6,19,23-tetramethyl-10,15-dimethylenetetracosa-2,6,11,18,22-pentaene
Farnesene thermal dimers	3, 4, 5, 6	3: 1-((*E*)-4,8-dimethylnona-3,7-dien-1-yl)-4-((*E*)-6,10-dimethylundeca-1,5,9-trien-2-yl)cyclohex-1-ene<br>4: 1-((*E*)-4,8-dimethylnona-3,7-dien-1-yl)-5-((*E*)-6,10-dimethylundeca-1,5,9-trien-2-yl)cyclohex-1-ene<br>5: 1,4-bis((E)-4,8,dimethylnona-3,7-dien-1-yl)-5-vinylcyclohex-1-ene<br>6: 1,4-bis((E)-4,8,dimethylnona-3,7-dien-1-yl)-4-vinylcyclohex-1-ene
C_{20} dimer	7	(*E*)-2,15-dimethyl-6,11-dimethylenehexadeca-2,7,14-triene
C_{25} dimer	8	(6*E*,13*E*)-2,6,19-trimethyl-10,15-dimethyleneicosa-2,6,13,18-tetraene
Solanesol	9	(2*E*,6*E*,10*E*,14*E*,18*E*,22*E*,26*E*,30*E*)-3,7,11,15,19,23,27,31,35-nonamethylhexatriaconta-2,6,10,14,18,22,26,30,34-nonaen-1-ol
Alcohols A	10	(5-((*E*)-7,11-dimethyl-3-methylenedodeca-6,10-dien-1-yl)-3-((*E*)-4,8-dimethylnona-3,7-dien-1-yl)cyclohex-3-en-1-yl)methanol
Squalane	11	2,6,10,15,19,23-hexamethyltetracosane
Difarnesyl ether	12	(2*E*,6*E*)-3,7,11-trimethyl-1-(((2*E*,6*E*)-3,7,11-trimethyldodeca-2,6,10-trien-1-yl)oxy)dodeca-2,6,10-triene
Alcohols C	13	(5-((*E*)-7,11-dimethyl-3-methylenedodeca-6,10-dien-1-yl)-3-((*E*)-4,8-dimethylnona-3,7-dien-1-yl)-1-methylcyclohex-3-en-1-yl)methanol
Aldehydes A	14	5-((*E*)-7,11-dimethyl-3-methylenedodeca-6,10-dien-1-yl)-3-((*E*)-4,8-dimethylnona-3,7-dien-1-yl)cyclohex-3-ene-1-carbaldehyde
Compounds 1	15	3-((*E*)-7,11-dimethyl-3-methylenedodeca-6,10-dien-1-yl)-5-((*E*)-4,8-dimethylnona-3,7-dien-1-yl)cyclohex-4-ene-1,2-dicarboxylic acid
Acids A	16	5-((*E*)-7,11-dimethyl-3-methylenedodeca-6,10-dien-1-yl)-3-((*E*)-4,8-dimethylnona-3,7-dien-1-yl)cyclohex-3-ene-1-carboxylic acid
Acids C	17	4-((5-((*E*)-7,11-dimethyl-3-methylenedodeca-6,10-dien-1-yl)-3-((*E*)-4,8-dimethylnona-3,7-dien-1-yl)cyclohex-3-en-1-yl)methoxy)-4-oxobutanoic acid
Esters A	18	butyl 5-((*E*)-7,11-dimethyl-3-methylenedodeca-6,10-dien-1-yl)-3-((*E*)-4,8-dimethylnona-3,7-dien-1-yl)cyclohex-3-ene-1-carboxylate
Diols B	19	(3-((*E*)-7,11-dimethyl-3-methylenedodeca-6,10-dien-1-yl)-5-((*E*)-4,8-dimethylnona-3,7-dien-1-yl)cyclohex-4-ene-1,2-diyl)dimethanol
Compounds 3	20	Mixture of compounds
Esters C	21	butyl 5-((*E*)-7,11-dimethyl-3-methylenedodeca-6,10-dien-1-yl)-3-((*E*)-4,8-dimethylnona-3,7-dien-1-yl)-1-methylcyclohex-3-ene-1-carboxylate
Diesters B	22	dimethyl 3-((*E*)-7,11-dimethyl-3-methylenedodeca-6,10-dien-1-yl)-5-((*E*)-4,8-dimethylnona-3,7-dien-1-yl)cyclohex-4-ene-1,2-dicarboxylate
Alcohols D	23	(4*E*,8*E*)-5,9,13-trimethyl-2-((2*E*,6*E*)-3,7,11-trimethyldodeca-2,6,10-trien-1-yl)tetradeca-4,8,12-trien-1-ol
Ether 2	24	1,3-bis(((2*E*,6*E*)-3,7,11-trimethyldodeca-2,6,10-trien-1-yl)oxy)benzene
Ether 3	25	1,2-bis(((2*E*,6*E*)-3,7,11-trimethyldodeca-2,6,10-trien-1-yl)oxy)ethane
Ether 4	26	(*E*)-1,4-bis(((2*E*,6*E*)-3,7,11-trimethyldodeca-2,6,10-trien-1-yl)oxy)but-2-ene
Ether 5	27	1,2-bis(((2*E*,6*E*)-3,7,11-trimethyldodeca-2,6,10-trien-1-yl)oxy)benzene
Ether 6	28	1,3,5-tris(((2*E*,6*E*)-3,7,11-trimethyldodeca-2,6,10-trien-1-yl)oxy)benzene

Supplementary Table 2. Experimental groups for each mouse immunogenicity study

**Mouse Experiment #1**	**Mouse Experiment #2**	**Mouse Experiment #3**
Antigen (Ag) alone	Antigen (Ag) alone	Antigen (Ag) alone
Ag + shark squalene SE	Ag + shark squalene SE	Ag + shark squalene SE
Ag + long-chain triglyceride SE	Ag + long-chain triglyceride SE	Ag + ether 2 SE
Ag + DHIS SE	Ag + difarnesyl ether SE	Ag + ether 3 SE
Ag + farnesene thermal dimers SE	Ag + alcohols C SE	Ag + ether 4 SE
Ag + C20 dimer SE	Ag + aldehydes A SE	Ag + ether 5 SE
Ag + C25 dimer SE	Ag + acids A SE	Ag + shark squalene MF59-like
Ag + solanesol SE	Ag + esters A SE	Ag + diols B MF59-like
Ag + alcohols A SE	Ag + alcohols A SE	
Ag + squalane SE		
Ag + shark squalane SE		

Supplementary Table 3. Effect of inserting chain-extending core substituents on composite desirability score

Compound Name	Number of connecting atoms between the two farnesyl groups	cLogP	Composite desirability score of emulsified compound with vaccine antigen
ether 4	6	11.4	0.521
difarnesyl ether	1	13.4	0.509
ether 5	4	13.3	0.504
ether 3	4	11.2	0.477
ether 2	5	13.7	0.401
