# Supplementary material for: Semi-synthetic terpenoids with differential adjuvant properties as sustainable replacements for shark squalene in vaccine emulsions
Source: NPJ Vaccines. 2023 Feb 16;8:14. doi: 10.1038/s41541-023-00608-y (PMC9935550; doi:10.1038/s41541-023-00608-y)
Supplement: Supplementary file 3 — REPORTING SUMMARY [file 41541_2023_608_MOESM3_ESM.pdf]

nature portfolio

Corresponding author(s): Christopher B. Fox

Last updated by author(s): 2022-Dec-22

Reporting Summary

Nature Portfolio wishes to improve the reproducibility of the work that we publish. This form provides structure for consistency and transparency in reporting. For further information on Nature Portfolio policies, see our Editorial Policies and the Editorial Policy Checklist.

Statistics

For all statistical analyses, confirm that the following items are present in the figure legend, table legend, main text, or Methods section.

n/a	Confirmed	
☐	☒	The exact sample size (*n*) for each experimental group/condition, given as a discrete number and unit of measurement
☐	☒	A statement on whether measurements were taken from distinct samples or whether the same sample was measured repeatedly
☐	☒	The statistical test(s) used AND whether they are one- or two-sided<br>*Only common tests should be described solely by name; describe more complex techniques in the Methods section.*
☐	☒	A description of all covariates tested
☐	☒	A description of any assumptions or corrections, such as tests of normality and adjustment for multiple comparisons
☐	☒	A full description of the statistical parameters including central tendency (e.g. means) or other basic estimates (e.g. regression coefficient) AND variation (e.g. standard deviation) or associated estimates of uncertainty (e.g. confidence intervals)
☐	☒	For null hypothesis testing, the test statistic (e.g. *F*, *t*, *r*) with confidence intervals, effect sizes, degrees of freedom and *P* value noted<br>*Give P values as exact values whenever suitable.*
☒	☐	For Bayesian analysis, information on the choice of priors and Markov chain Monte Carlo settings
☒	☐	For hierarchical and complex designs, identification of the appropriate level for tests and full reporting of outcomes
☐	☒	Estimates of effect sizes (e.g. Cohen's *d*, Pearson's *r*), indicating how they were calculated

Our web collection on statistics for biologists contains articles on many of the points above.

Software and code

Policy information about availability of computer code

Data collection	ELISA plates were read using Perkin Elmer VictorX4 2030 or Biotek Synergy 2. ELISpot data was collected using ImmunoSpot v7 Professional.
Data analysis	GraphPad Prism v9.3.1 was employed for statistical analysis of biological data.

For manuscripts utilizing custom algorithms or software that are central to the research but not yet described in published literature, software must be made available to editors and reviewers. We strongly encourage code deposition in a community repository (e.g. GitHub). See the Nature Portfolio guidelines for submitting code & software for further information.

Data

Policy information about availability of data

All manuscripts must include a data availability statement. This statement should provide the following information, where applicable:

- Accession codes, unique identifiers, or web links for publicly available datasets
- A description of any restrictions on data availability
- For clinical datasets or third party data, please ensure that the statement adheres to our policy

The datasets generated during and/or analysed during the current study are available from the corresponding author on reasonable request. Materials were transferred between institutions under a Material Transfer Agreement (MTA), and MTAs would be required for any sample requests.

Human research participants

Policy information about studies involving human research participants and Sex and Gender in Research.

Reporting on sex and gender	Whole blood was collected from equal numbers of male and female donors for in vitro cytokine stimulation assays. However, the study was not powered to detect significant differences in response based on sex. Sex-specific data is available upon request.
Population characteristics	Whole blood samples were not controlled for differences in population characteristics other than sex.
Recruitment	Whole blood was obtained from Bloodworks Northwest. All participants reviewed and signed informed consent forms.
Ethics oversight	Blood collections were approved by WCG IRB.

Note that full information on the approval of the study protocol must also be provided in the manuscript.

Field-specific reporting

Please select the one below that is the best fit for your research. If you are not sure, read the appropriate sections before making your selection.

☒ Life sciences ☐ Behavioural & social sciences ☐ Ecological, evolutionary & environmental sciences

For a reference copy of the document with all sections, see nature.com/documents/nr-reporting-summary-flat.pdf

Life sciences study design

All studies must disclose on these points even when the disclosure is negative.

Sample size	Sample sizes were determined based on previous experience in vaccine adjuvant studies regarding the minimum number of subjects necessary to identify statistically significant differences between experimental groups.
Data exclusions	Data were only excluded if there were obvious errors in sample preparation (e.g., low viability) or instrumentation.
Replication	The same negative and positive controls (antigen alone and shark squalene emulsion, respectively) were employed in each in vitro human whole blood study and each mouse immunogenicity study to establish consistency across experiments. Stability measurements consisted of multiple measurements at each time point.
Randomization	After controlling for sex, animals were randomly assigned to experimental groups. Blood from the same human subjects was used across all experimental groups for the in vitro assays.
Blinding	Blinding was not implemented due to the screening nature of the studies and the large number of compounds evaluated.

Reporting for specific materials, systems and methods

We require information from authors about some types of materials, experimental systems and methods used in many studies. Here, indicate whether each material, system or method listed is relevant to your study. If you are not sure if a list item applies to your research, read the appropriate section before selecting a response.

Materials & experimental systems

n/a	Involved in the study
☐	☒ Antibodies
☒	☐ Eukaryotic cell lines
☒	☐ Palaeontology and archaeology
☐	☒ Animals and other organisms
☒	☐ Clinical data
☒	☐ Dual use research of concern

Methods

n/a	Involved in the study
☒	☐ ChIP-seq
☒	☐ Flow cytometry
☒	☐ MRI-based neuroimaging

Antibodies

Antibodies used	Horseradish peroxidase (HRP)-conjugated IgG (catalog# 1031-05), IgG1 (catalog# 1070-05), and IgG2c (catalog# 1079-05) were purchased from Southern Biotech. Cytokine-specific antibodies for mouse study ELISAs were purchased as part of kits from

	Invitrogen (IL-5 ELISA kit catalog# 88-7054-88, IFN-g ELISA kit catalog# 88-7314-88). Cytokine-specific antibodies for mouse study ELISpot assays were purchased as part of kits from BD Biosciences (IFN-g catalog# 551881, IL-5 catalog# 551880) and eBioscience (IFN-g catalog# 88-7384, IL-5 catalog# 88-7825). Avidin-HRP was purchased from Invitrogen (catalog# 50-112-3249). Cytokine-specific antibodies for human whole blood ELISAs were purchased as part of kit components from Life Technologies (IL-8 catalog# 88-8086-77, MCP-1 catalog# 88-7399-77, IL-6 catalog# 88-7066-77) and R&D Systems (MIP-1b catalog# DY271).
Validation	Antibody reagents were acquired from well-established vendors as listed above. Certificates of analysis and/or technical information sheets are available from the vendors.

Animals and other research organisms

Policy information about studies involving animals; ARRIVE guidelines recommended for reporting animal research, and Sex and Gender in Research

Laboratory animals	C56BL/6 inbred mice, 6-8 wks old, equal numbers male and female, were purchased from Jackson Laboratories.
Wild animals	The study did not involve wild animals.
Reporting on sex	Equal numbers of male and female mice were used in the immunogenicity studies with the exception of the preliminary mouse dose finding study reported in the supplementary information which involved only female mice. Mouse studies were not powered to detect significant differences in response based on sex. Sex-specific data is available upon request.
Field-collected samples	The study did not involve samples collected from the field.
Ethics oversight	All animal experiments were performed in accordance with national and institutional guidelines for animal care of laboratory animals and approved by the AAHI Institutional Animal Care and Use Committee.

Note that full information on the approval of the study protocol must also be provided in the manuscript.
